# A dual-mode transparent device for 360° quasi-omnidirectional self-driven photodetection and efficient ultralow-power neuromorphic computing

**DOI:** 10.1038/s41377-025-01991-y

**Published:** 2025-08-12

**Authors:** Min Jiang, Yukun Zhao, Tong Liu, Yanyan Chang, Yuan Tang, Min Zhou, Yiping Shi, Jianya Zhang, Lifeng Bian, Shulong Lu

**Affiliations:** 1 School of Nano-Tech and Nano-Bionics, University of Science and Technology of China (USTC), Hefei, 230026 China; 2 Division of Nano-Devices Research, Suzhou Institute of Nano-Tech and Nano-Bionics (SINANO), Chinese Academy of Sciences (CAS), Suzhou, 215123 China; 3 Key Laboratory of Semiconductor Display Materials and Chips, SINANO, CAS, Suzhou, 215123 China; 4 Vacuum Interconnected Nanotech Workstation, SINANO, CAS, Suzhou, 215123 China; 5 Key Laboratory of Intelligent Optoelectronic Devices and Chips of Jiangsu Higher Education Institutions, School of Physical Science and Technology, Suzhou University of Science and Technology, Suzhou, 215009 China; 6 Frontier Institute of Chip and System, Fudan University, Shanghai, 200433 China

**Keywords:** Nanowires, Photonic devices, Optoelectronic devices and components

## Abstract

Due to the extremely high manufacturing standards, the integration of quasi-omnidirectional photodetectors and synaptic devices within a single device remains a long-standing challenge. In this work, we have designed a graphene/(Al,Ga)N nanowire heterojunction, demonstrating the monolithic integration of self-driven 360° photodetectors and artificial synapses in a dual-mode transparent device successfully. By manipulating the carrier transport dynamics through controlling the bias voltage, the degree of oxygen vacancy ionization can be precisely regulated, ultimately realizing the monolithic dual-mode device. At 0 V bias, the device functions as a fast-response self-driven photodetector with stable optical communication capabilities, achieving 360° quasi-omnidirectional photodetection. Upon applying a bias voltage, the operating mode switches to a synaptic device, which successfully simulates brain-like paired-pulse facilitation, short-/long-term plasticity processes, and learning/forgetting behaviors. The device demonstrates an exceptionally high UV/visible rejection ratio of 1.29 × 10^4^, coupled with an ultra-low dark current of less than 1 pA. Furthermore, this device has a low power consumption of 2.5 × 10^−14^ J per synaptic event, indicating an energy efficiency comparable to synaptic processes in the human brain. Moreover, nonlinear photoconductivity lets the device become a neuromorphic sensor for preprocessing images, enhancing recognition accuracy. Importantly, by leveraging the long-memory characteristic of the devices in open-circuit voltage mode, the devices have been successfully applied to guide humanoid robots in performing direction distinguishing and motion learning. This work provides new insights into the integrated manufacturing of multifunctional monolithic devices and foresees their immense potential in upcoming advanced, low-power neuromorphic computing systems.

## Abstract (web summary)

Development of a dual-mode device for driving humanoid robot.

## Graphical abstract

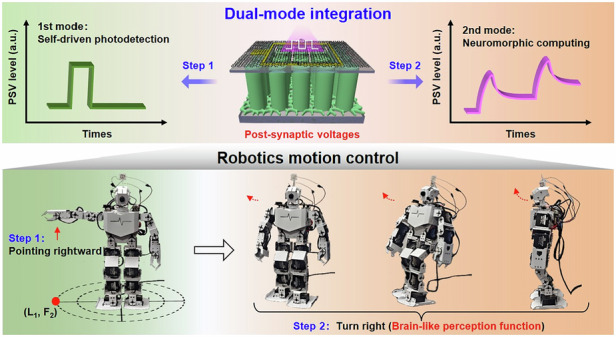

## Introduction

A photodetector can quickly and sensitively convert light signals into electrical signals, thereby achieving the collection, transmission, and analysis of information^1–6^. These detectors are crucial components for human perception of the external world and are widely used in various fields such as aerospace, non-line-of-sight communication, industrial manufacturing, fundamental science, and medical equipment research^7–10^. On the other hand, the human visual system not only captures light signals but also performs complex image processing and memory storage in the brain, achieving efficient information processing and response^11,12^. Inspired by the human visual system, a neural computing architecture has been proposed that can simultaneously store and process data to improve computational efficiency with the smallest operational unit being the synaptic device^13–15^. This type of neuromorphic visual device can not only sense light signals from the environment but also process these signals through internal synaptic devices, exhibiting adaptive image recognition and analysis capabilities. Self-driven photodetectors are indispensable devices for various applications due to their the advantage of ultralow energy consumption^16^. Therefore, once self-driven photodetectors and synaptic devices are integrated simultaneously on a single device, not only can the complexity of the supporting system be reduced, but the application scenarios can also be expanded greatly. However, photodetectors typically rely on fast light response capabilities, while synaptic devices require more time for signal processing and storage^17–19^. Photodetectors cannot remember images and process light signals as synaptic devices do. In other words, such a significant contradiction exists in their light response speeds, making it difficult to integrate them into a single unit for synchronized and efficient operation.

Compared to bulk and thin-film III-V compound semiconductor materials, GaN nanowire (NW) nanostructures possess a high surface-to-volume ratio, excellent crystalline quality, and anisotropic geometric shapes, which endow them with unique optical and chemical properties^20–23^. Importantly, several other critical factors make them suitable for the fabrication of dual-mode composite devices, including the persistent photoconductivity (PPC) effect for constructing synaptic devices and high physical/chemical stability for ultraviolet (UV) photodetectors^22,24,25^. In our previous work, we have successfully demonstrated optically stimulated synaptic devices based on as-grown GaN nanowires^26,27^. In addition, the detection range of traditional photodetectors is limited to only 180°, thus requiring the use of two or more devices for quasi-omnidirectional detection^28^. The ability to detect light from 360° angles is another need for next-generation applications, which can be realized in highly transparent devices^29^. However, integrating 360° self-driven sensing and processing capabilities within a single transparent device requires much higher manufacturing standards. For example, the non-transparency of Si epitaxial substrates in the UV and visible light bands results in non-transparent devices^30^. Thus, such dual-mode transparent devices based on GaN nanowires have not yet been reported.

In this work, we propose and demonstrate a dual-mode switchable transparent optoelectronic device based on lift-off (Al,Ga)N nanowires without an epitaxial substrate. Under 0 V bias, the device exhibits a fast photoresponse due to the built-in field at the graphene/(Al,Ga)N heterojunction interface, making it suitable for use as a photodetector. Under positive/negative bias, the device exhibits significant PPC effects, thus achieving synaptic response characteristics. This is attributed to the improvement of the separation efficiency of photogenerated carriers under the external electric field, which in turn promotes the ionization of oxygen vacancies. This phenomenon implies that our device can switch between a photodetector and a neuromorphic visual sensor by applying a bias voltage. Thus, as a photodetector, it can achieve 360° quasi-omnidirectional photodetection and ultra-low dark current, and we have demonstrated its wireless optical communication capability in both the positive 90° and negative 90° directions. As an artificial synapse, the device not only exhibits tunable synaptic properties but also ultra-low power consumption and a high UV–visible rejection ratio. Furthermore, the device has been applied to a neuromorphic image preprocessing system successfully, improving the accuracy and efficiency of the pre-processed images. Significantly, by connecting a dual-mode device to a humanoid robot and detecting the PSV signals from two orthogonally placed devices, we have achieved discrimination across eight directions. Additionally, based on the long-term plasticity (LTP) in response to the light signals, we implemented motion control of the robot for moving forward, turning right, and turning backward successfully. Despite the challenges, the combination of photodetectors and neuromorphic synaptic devices is expected to drive the development of more efficient and intelligent visual sensors.

## Results

### Design of a transparent dual-mode monolithic device

**Fig. 1:**
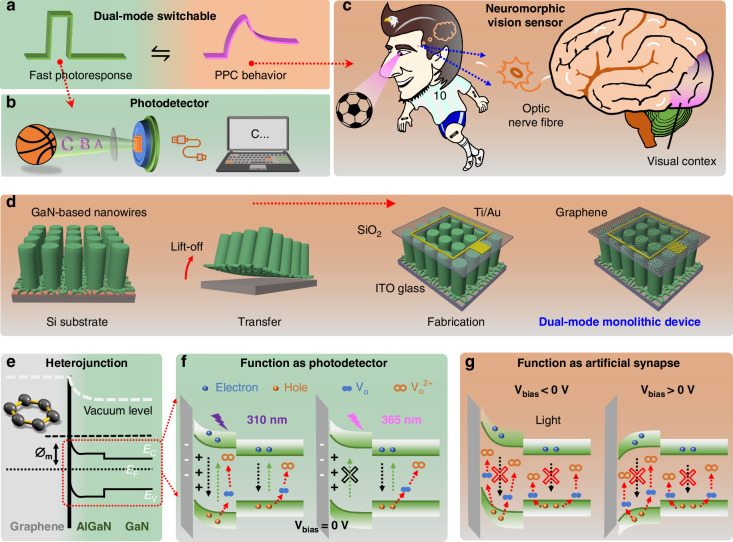
Design of dual-mode transparent monolithic device based on (Al,Ga)N/GaN nanowires. **a** The operation mode switchable concept of the device. The application scenarios of **b** photodetector and **c** neuromorphic vision sensor based on different photoresponse behaviors. **d** Schematic illustrations of the fabricated processes for the dual-mode monolithic device, including the lift-off and transfer of nanowires film, the SiO_2_ graphic passivation, the fabrication processes of the top Ti/Au electrode and graphene current collecting layer. **e** Formation of graphene/(Al,Ga)N heterojunction and the energy band diagrams of (Al,Ga)N/GaN nanowires under dark conditions. Under 310/365 nm illumination, the device functions as **f** a photodetector at 0 V bias and **g** a synaptic device at negative/positive bias. The bandgap widths of (AI,Ga)N and GaN are *E*_g1_ and *E*_g2_, respectively. The green arrows in (**f**) and (**g**) represent the generation of charge carriers, the black arrows represent the recombination of charge carriers, and the red arrows represent the ionization of V_O_. The “×” symbol indicates that the process does not occur or is severely inhibited

Physically, the photo-electric conversion occurs rapidly when the photodetector receives light signals, transmitting them to the external equipment, while this process of artificial synapse is usually accompanied by mnemonic behavior that relies on PPC behavior^17,31^. Based on the photoresponse behaviors in the photodetector and artificial synapse, the differences can be vividly described as “volatile/fast photoresponse” and “non-volatile/PPC behavior” photocurrents (Fig. 1a). For a dual-mode device, when switched to the photodetector mode, it can serve as an important optical sensing component in an optical signal detection system (Fig. 1b). When switched to the neuromorphic vision sensor (synaptic device) mode, it can act as a core component for applications such as brain-like computing and neuromorphic perception (Fig. 1c). Our transparent dual-mode monolithic device is composed of (Al,Ga)N/GaN nanowires that are grown on the Si(111) substrate by molecular beam epitaxy (“Materials and Methods”). Figure S1 shows that each nanowire is composed of a 280 nm-high (Al,Ga)N segment and a 1900 nm-high GaN (*E*_g2_) segment along the axial direction. The peak of the photoluminescent curve is centered at 330 nm (Fig. S2), indicating the bandgap of (Al,Ga)N is about 3.8 eV (*E*_g1_). Figure 1d describes the detailed fabrication process of the transparent device. Prior to conventional semiconductor device preparation, a convenient and efficient electrochemical detaching method is introduced to detach the (Al,Ga)N/GaN nanowires from original Si substrate and then transfer them to the external ITO/glass substrate. Graphic SiO_2_ passivation is coated close behind, and the Ti/Au top electrode is deposited above the passivation layer, which is slightly larger than the square window area. Thereafter, due to the gaps between each nanowire, a current collecting layer is prepared by graphene to coat the window area to complete the dual-mode monolithic device.

The construction of heterojunctions has resulted in the development of a diverse range of photodetectors exhibiting “volatile” current characteristics^32^. Conversely, the “non-volatile” current phenomena observed in artificial synapses can be attributed to the trapping and de-trapping of carriers in defects^33,34^. Figure 1e illustrates the heterojunction formed at the graphene/(Al,Ga)N interface, alongside the energy band diagrams of (Al,Ga)N/GaN nanowires. Under dark conditions, the barrier at the graphene/(Al,Ga)N interface helps maintain a low dark current of the device. Upon 310 nm light (*hv*_*1*_ ≈ 4.0 eV), all regions of the nanowires absorb photons (*hv*_*1*_ > *E*_g1_ > *E*_g2_). However, when exposed to 365 nm light, only the GaN segment effectively absorbs photons due to the relationship *E*_g1_ > *hv*_2_ ≈ *E*_g2_. Importantly, under a bias voltage of 0 V, whether illuminated by light at 310 nm or 365 nm, only the built-in electric field at the graphene/(Al,Ga)N heterojunction can effectively separate electron-hole pairs (e^−^-h^+^), while the photogenerated carriers in the rest of the nanowire will diffuse along the concentration gradient, providing an opportunity to regulate the participation of carriers in the ionization of oxygen vacancy (V_O_) through bias voltage. V_O_ is a common type of lattice defect that can induce PPC behavior in GaN-based devices^25,27,35^, existing in a neutral state. However, ionized Vo carries with a positive charge can capture electrons, influencing the response characteristics of the device after turning off the light, manifesting as a persistent photoconductive (PPC) effect. When light irradiates the surface of the device, (Al,Ga)N/GaN nanowires absorb photons, resulting in the generated holes subsequently engaging in the ionization of Vo. Specifically, photogenerated holes possess high oxidizing power, and they oxidize Vo into the ionized state (Vo^2+^) when holes encounter it. The reaction equation is expressed as: Vo + 2 h^+^ → Vo^2+^^33^. In this process, Vo^2+^ becomes positively charged due to the loss of two electrons, and its state density and energy level position change accordingly, acting as a negative charge trapping center. Under continuous illumination, more Vo are ionized into Vo^2+^. The number of free electrons in the conduction band increases, exhibiting an increasingly enhanced photocurrent. Since Vo^2+^ accumulates a large number of electrons during illumination, it gradually releases electrons upon turning off the light, allowing the device to maintain a high carrier concentration within a period time and resulting in a long relaxation time.

This implies that a greater number of holes in the nanowire participating in the ionization of V_O_ will lead to the positively charged centers (V_O_^2+^) capturing a substantial number of free electrons, ultimately resulting in a notable PPC effect associated with a prolonged carrier decay time. In other words, if we can effectively control the degree of hole participation in V_O_ ionization within the nanowire under illumination, there is a high likelihood of achieving monolithic integration of photodetectors and synaptic devices. As depicted in Fig. 1f, when the device is exposed to 310 nm or 365 nm light at a bias voltage of 0 V, charge carriers will diffuse based on the concentration gradient due to the absence of an external electric field, ultimately leading to substantial carrier recombination. Therefore, only a limited number of holes are able to participate in the ionization of V_O_ and the PPC effect is not prominent, allowing the device to function effectively as a photodetector with a relatively rapid response speed. By contrast, as illustrated in Fig. 1g, the application of positive/negative bias voltage results in a reduction of carrier recombination due to the external electric field. At this stage, carriers can be efficiently separated, and a significant number of holes within the nanowire engage in the ionization of V_O_ during transportation, ultimately producing the PPC effect upon which synaptic devices depend. Figure S3 depicts the detailed process of synaptic device characteristics manifested by the device when subjected to continuous periodic light signal stimulation. Figure S4 and Supplementary Note 1 provide detailed explanations of the physical mechanism by which carriers can be effectively separated under the action of an external bias voltage. In short, we can control the generation of the PPC effect by applying a bias voltage to adjust the number of holes involved in the dynamic process of V_O_ ionization, thereby achieving a monolithic dual-mode device that can switch between a photodetector and a synaptic device. A dual-mode device integrating a self-driven quasi-omnidirectional photodetector and artificial synaptic device offers unique features and advantages: optical signal detection and neuromorphic signal processing based on detected signals, reducing energy consumption, and enabling novel intelligent system integration^18,36^. Supplementary Note 2 discusses the merits and collaborative applications of these devices.

### Structural and performance characterization

**Fig. 2:**
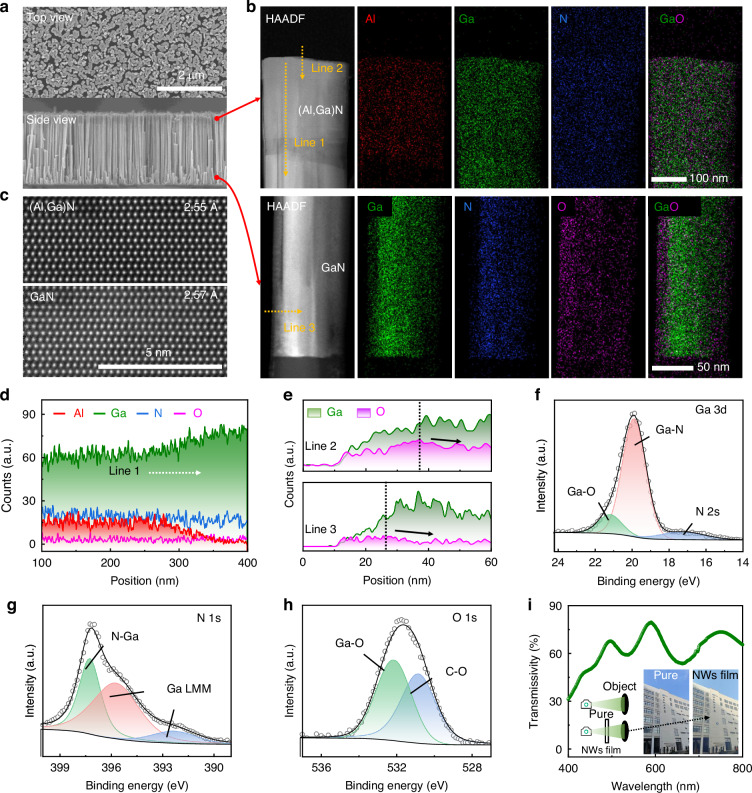
Structure and characterization of the (Al,Ga)N/GaN nanowires (NWs). **a** SEM images showing the top and side views of (Al,Ga)N/GaN nanowires. **b** HAADF-STEM images of the upper segment of (Al,Ga)N/GaN nanowires, accompanied by EDS mapping of Al, Ga, N, and O elements. **c** HAADF-STEM images of the (Al,Ga)N and GaN sections. Element proportion scanning lines **d** labeled line 1, **e** lines 2 and 3 on the surface of the (Al,Ga)N and GaN sections. XPS spectra of **f** Ga 3 d, **g** N 1 s, and **h** O 1 s in (Al,Ga)N nanowires. **i** Demonstration of the transparency of the lift-off (Al,Ga)N/GaN nanowires. Left inset: a photograph of the object without the nanowires film. Right inset: a photograph captured through the nanowires film

In Fig. 2a, the scanning electron microscope (SEM) images highlight the uniformity of the nanowire dimensions. According to the high-resolution high-angle annular dark-field scanning transmission electron microscopy (HAADF-STEM) image illustrated in Fig. 2b, a distinct contrast is evident, clearly differentiating the upper (Al,Ga)N segment from the underlying GaN segment. Figure 2c presents the atomic arrangement diagrams for both (Al,Ga)N and GaN in the m-plane, where the respective lattice constants are indicated as 2.55 Å and 2.57 Å. The energy dispersive spectrum (EDS) analysis images provide an intuitive confirmation of the distribution patterns of Al, Ga, N, and O elements. To gain a more detailed understanding of the elemental variations within the (Al,Ga)N/GaN nanowires, we performed elemental line scan analyses across three distinct regions on the nanowire surface (lines 1–3). In Fig. 2d, along line 1 traversing from the upper (Al,Ga)N layer to GaN, a pronounced decrease in the Al element and a corresponding rise in the Ga element are observable at the interface. Furthermore, lines 2 and 3 in Fig. 2e depict the variations in oxygen content across the surfaces of both the (Al,Ga)N and GaN segments in detail. It is evident that the oxygen content initially augments and subsequently diminishes from the exterior towards the interior, agreeing with the distribution pattern illustrated in the EDS image (Fig. 2b). The overlapping Ga/O signals in the analysis suggest the presence of a notable oxide layer (GaO_X_) on the surface of nanowires, indicating a high likelihood of V_O_ existing within it^37^.

The introduction of V_O_ primarily relies on the natural oxidation of (Al,Ga)N/GaN nanowires in the air and the synergistic effect of using nitric acid during the lift-off process^24,38^. X-ray photoelectron spectroscopy (XPS) is employed for further investigation into the presence of V_O_. Figure 2f presents the Ga 3 d core-level spectra of the (Al,Ga)N/GaN nanowires, wherein the Ga 3 d peak has been decomposed into three distinct components: Ga-N (ranging from 19.6 eV to 19.8 eV), Ga-O (spanning 20.6 eV to 20.8 eV), and N 2 s (located at 16.2 eV)^39^. Figure 2g distinctly shows three peaks precisely located at 397.1 eV (attributed to N-Ga bonding), 399.17 eV and 397.1 eV, stemming from Ga LMM Auger transitions^27,40^. The analyses of the O 1 s core level spectra in Fig. 2h reveal two peaks in the distinct bands, which is typically attributed to O^2−^ ions (~ 531.3 eV) and oxygen defects (~ 532.7 eV)^18,41^. In short, the results of EDS and XPS provide strong evidence for the existence of V_O_. Further detailed discussions on the introduction of V_O_ and the regulation of V_O_ concentration can be found in Fig. S5 and Supplementary Note 3.

To fabricate a transparent device, the (Al,Ga)N/GaN nanowires have been removed by the electrochemical detaching method from the as-grown Si substrate^42^. In essence, upon exposure to 365 nm light, the nanowires allow photons to traverse the upper (Al,Ga)N layer to directly reach the underlying GaN segment. This indicates that the (Al,Ga)N/GaN nanowires exhibit transparency to light wavelengths exceeding 365 nm, rendering them highly transparent within the visible wavelength spectrum (Fig. 2i). The inset in Fig. 2i presents a comparison between an object photographed without (left) and with (right) the nanowires film. While only a subtle difference in transparency is discernible, with the right image appearing slightly less sharp than the left one, the overall impact is not profound. It indicates that the dual-mode device based on the lift-off (Al,Ga)N nanowires film holds considerable promise for applications requiring high transparency. Figure S6 and Supplementary Note 4 discuss the transmittance loss in the visible wavelength range.

### Switchable photoresponse speed via bias voltage

**Fig. 3:**
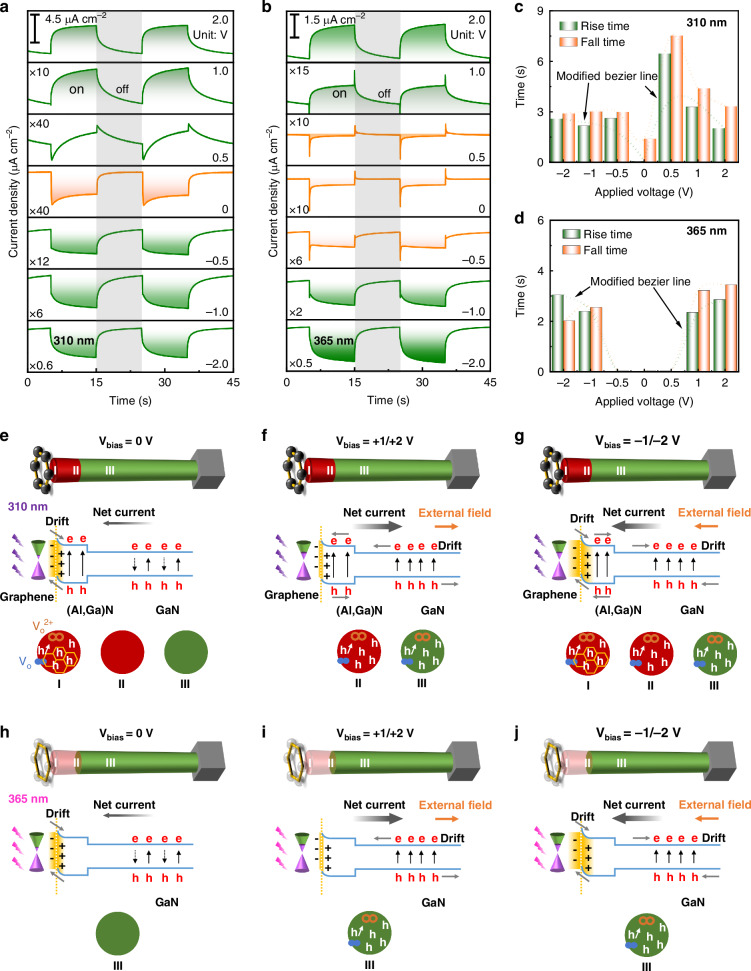
Operation mode switchable photoresponse characterization. The wavelength-dependent characteristics of the Graphene/(Al,Ga)N/GaN heterojunction nanowires at different biases under **a** 310 nm and **b** 365 nm illumination, respectively. ×10 means the current intensity of the *Y*-axis is enhanced 9 times for better comparison. The response time (rise/fall time) as a function of the applied biases extracted from the photocurrents under **c** 310 nm and **d** 365 nm illumination. Some response times are obtained through double exponential fitting. Hole distribution in nanowires and their involvement in the ionization of Vo under bias voltages of **e** 0 V, **f** +1/+2 V, and **g** −1/−2 V under 310 nm illumination. I, II, and III represent three regions of hole distribution within the nanowire, while the yellow regions of different widths depict the variation of the space charge region in the graphene/(AI,Ga)N heterojunction under different bias voltages. Hole distribution in nanowires and their involvement in the ionization of Vo under bias voltages of **h** 0 V, **i** +1/+2 V, and **j** −1/−2 V under 365 nm illumination. Transparency represents that this region in the nanowire does not play a dominant role

Thanks to the principles of epitaxial control, energy band structure design, and heterojunction interface engineering, we have employed lift-off (Al,Ga)N/GaN nanowires to successfully construct a dual-mode transparent device. In order to gain a deeper understanding of the switchable operating mode controlled by the bias voltage, Fig. 3a, b present the current-time response results of the dual-mode device under different bias conditions. Upon exposure to periodically alternating light, the dual-mode device consistently demonstrates reliable on-off switching capabilities, independent of whether it operates autonomously or under negative/positive bias conditions. As summarized in Fig. 3c, d, the response characteristics of the device under different bias voltages for these two illumination conditions are distinctly different. At 0 V bias, it exhibits a significantly faster response speed, while the synaptic response characteristics are prominent under high bias voltage. For GaN materials, although the carrier lifetime is related to material quality and defect density, it typically ranges from a few nanoseconds to tens of nanoseconds^43^. However, even though the ionization process of holes-inducing Vo is very rapid at room temperature, this reaction may occur on a timescale of microseconds to milliseconds, far exceeding the nanosecond range^44^. In our dual-mode device, under a 0 V bias (Fig. S4), the non-heterojunction region lacks the driving force to separate electron-hole pairs, leading to significant recombination. Consequently, carriers are unable to ionize Vo within their lifetime. Therefore, the PPC is insignificant under a 0 V bias, exhibiting rapid photoresponse characteristics, which is consistent with the experimental results shown in Fig. 3a, b. Conversely, a high external bias electric field can accelerate the separation of carriers, enabling holes to continuously contact the V_O_ on the nanowire surface during transport, thereby participating in the ionization of Vo and exhibiting a gradually enhanced photocurrent. From the schematic diagram of the device structure (Fig. 3e–g), the device can be considered in three parts from top to bottom: the graphene/(Al,Ga)N heterojunction region (I), (Al,Ga)N region (II), and the GaN region (III). The width of the depletion zone (region I, *W*) can be calculated according to the following formula^45^:

1 $$W=\sqrt{\frac{{2\varepsilon }_{{GaN}}\Delta \phi }{{{qN}}_{{GaN}}}}$$

*ε*_GaN_ = 9.5 *ε*_0_ (*ε*_0_ is vacuum dielectric constant). ∆∅ = ∅_Graphene_-∅_GaN_ ≈ 0.5 eV (the electron affinity energies of graphene and GaN are 4.6 eV and 4.1 eV, respectively). *q* = 1.6 × 10^−19^ C. *N*_GaN_ ≈ 10^16^ cm^−^^3^ (unintentional or light doping). The calculated *W* is 120 nm, while the non-heterojunction region is approximately 2000 nm. Therefore, we can approximately consider the proportion of Vo as the ratio of the surface area of the graphene/(Al,Ga)N heterojunction region to that of other non-heterojunction regions (*S*_top_/*S*_side_ < 1/10), clearly indicating that Vo dominates in the non-heterojunction regions. When only a small number of holes are transported in the nanowires (under 0 V bias), although some of the holes in the heterojunction region participate in the Vo ionization process, the insufficient number of vacancies results in insignificant ionization of Vo, and the PPC effect is also not pronounced after turning off the light. However, when a large number of holes are transported in the device (under high bias), a significant amount of Vo on the sidewalls of the nanowires will participate in the ionization process, leading to a strong PPC effect.

In the heterojunction region (region I, Fig. 3e), due to the presence of a built-in electric field, the photogenerated carriers can be effectively separated. As shown in the photoresponse characteristics in Fig. 3a and e, a negative photocurrent is generated, which mainly originates from the separation and transport of carriers in the graphene/(Al,Ga)N heterojunction region. However, the diffusion of photogenerated carriers in the non-heterojunction region (regions II and III) mainly depends on concentration gradients. The generated carriers will spontaneously recombine, resulting in only a low-level photocurrent being detected in the external system. At this time, only a small number of holes participate in the ionization process of V_O_, so there is no gradually increased photocurrent at 0 V bias voltage. At a 0.5 V bias voltage, the photoresponse characteristic curve exhibits a transition from negative to positive (Fig. 3a). When 310 nm light illuminates the device surface, the built-in electric field in the graphene/(Al,Ga)N heterojunction region quickly generates a negative photocurrent (consistent with the behavior at 0 V). However, under the influence of the external bias electric field (which is opposite to the direction of the built-in electric field), carriers in the non-heterojunction region gradually take the dominant role, ultimately leading to a positive photocurrent. At this point, as some holes participate in the ionization process of V_O_ in the non-heterojunction region during transport, the PPC effect gradually emerges, manifesting as a slow increase in the photocurrent. At +1 V or +2 V bias voltages (Fig. 3f), the separation efficiency of photogenerated carriers in the non-heterojunction region further increases with positive photocurrents. Comparing the photoresponse current levels at +1 V and +2 V bias voltages in Fig. 3a, it can be seen that the carrier separation efficiency is higher at +2 V bias voltage, resulting in a larger photoresponse current. From Fig. 2b–h, a large number of V_O_ are located on the surface of the nanowires, and a large number of holes participate in the ionization of vacancies in the sidewall region during transport. Under negative bias voltage (Fig. 3g), the direction of the electric field is the same as the direction of the built-in electric field. Therefore, the photoresponse currents at −0.5, −1, and −2 V bias voltages are all slightly larger than those under positive bias voltages.

Under 365 nm illumination (Fig. 3b), neither the top graphene/(Al,Ga)N heterojunction region nor the (Al,Ga)N nanowire region absorbs photons due to the relationship between photon energy and the semiconductor bandgap. The bottom GaN region absorbs the major 365 nm photons (region III). At 0 V bias voltage, photogenerated carriers diffuse according to concentration gradients, leading to rapid recombination. As shown in Fig. 3h, without sufficient holes participating in the ionization of V_O_ in the sidewall region, no significant PPC effect is observed. Additionally, when some carriers extend into the heterojunction region, they are separated by the built-in electric field, resulting in self-driven characteristics at 0 V bias. Under a bias voltage of 0.5 V, where the external bias does not dominate, and the internal and external electric fields weaken each other, carrier recombination becomes more pronounced without a significant PPC effect. At 1 V bias voltage, the external bias voltage dominates, causing a reversal in current polarity (Fig. 3b). At this point, some holes from the bottom GaN region participate in the ionization of V_O_ in the sidewall during transport, manifesting as a gradually increasing photocurrent (Fig. 3i). At 2 V bias voltage, photogenerated carriers are more effectively separated, and more holes participate in the ionization of V_O_ in the sidewall during transport, producing a more pronounced PPC effect. At −0.5 V bias voltage, the external bias electric field is in the same direction as the built-in electric field. However, there are still not enough holes participating in the ionization of V_O_ in the sidewall, so no significant PPC effect is observed. At −1 V and −2 V bias voltages (Fig. 3j), the external bias voltage dominates, and more holes participate in the ionization of V_O_ in the sidewall during transport, leading to a more pronounced PPC effect (Fig. 3b). The photocurrent at −2 V is significantly greater than that at −1 V, further proving that carriers are more effectively separated at larger bias voltages, allowing more holes to participate in the ionization of V_O_ during transport. In summary, the applied bias is a key factor influencing the PPC in the (Al,Ga)N/GaN nanowires, enabling a switchable operating mode in the device. Specifically, at 0 V bias, the PPC effect is feeble, resulting in a rapid device response speed. Conversely, the higher bias voltages induce a robust PPC effect, causing a slower response speed. To further study the impact of bias voltage on the number of holes involved in V_O_ ionization, the I-V hysteresis curves have been measured and shown in Fig. S7. Detailed discussions are available in Supplementary Note 5. Therefore, the dual-mode device can function as a self-driven photodetector at 0 V bias, while it can be seamlessly transitioned to an artificial synapse under relatively high positive or negative bias.

### Function as quasi-omnidirectional photodetector

**Fig. 4:**
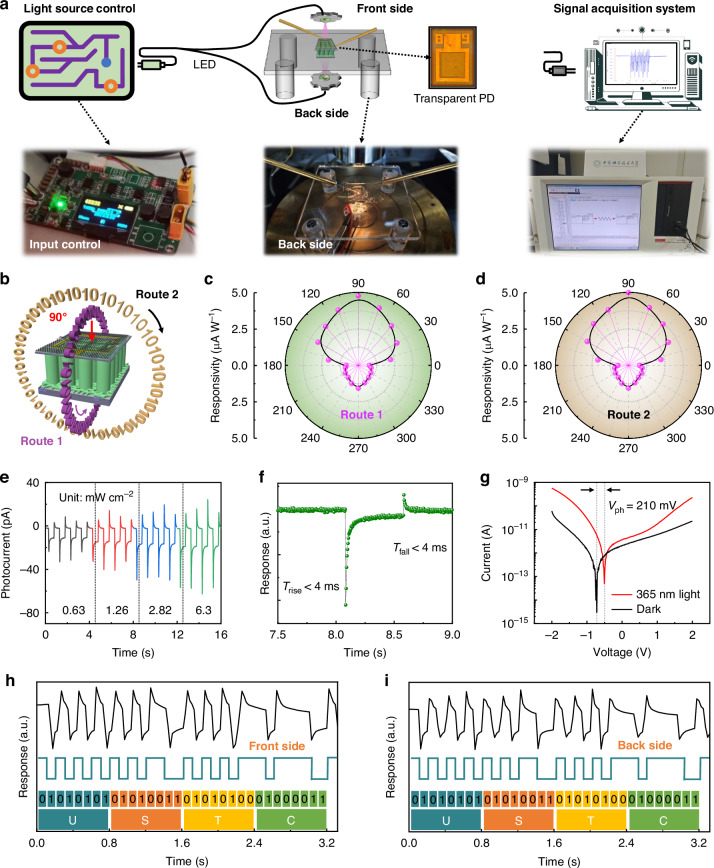
Performance demonstration of the dual-mode transparent device in 360° quasi-omnidirectional photodetection and wireless communication at 365 nm and 0 V bias. **a** Schematic diagram of building performance testing system for a transparent device. **b** The schematic diagram illustrates the quasi-omnidirectional photodetection process under routes 1 and 2 along two perpendicular directions. The responsivity of the device as a function of the incident light angle along with **c** route 1 and **d** route 2. **e** Photocurrent of the graphene/(Al,Ga)N heterojunction-based dual-mode device under increased power intensity. **f** The response time of the dual-mode device. **g** The current-voltage curve of the device under dark and 365 nm light. In light conditions, the device generates a photovoltage of approximately 210 mV, which can be easily detectable by an external circuit. The performance of the device in wireless optical communication systems at **h** front side (90°) and **i** back side (270°)

As outlined in Fig. 4a, to evaluate the 360° quasi-omnidirectional photodetection capabilities of the device under a 0 V bias voltage, we set a transparent frame and a system to demonstrate it. Figure 4b depicts a schematic diagram outlining the 360° photodetection mechanism, wherein the planes traversed by test line 1 and line 2 are positioned at right angles to each other. The quasi-omnidirectional photodetection capabilities of the device can be thoroughly exhibited along these two perpendicular directions, with the top part of the device defined as 90° incident light (Fig. 4b). The photoresponse behaviors and the corresponding photocurrent of the device are thoroughly illustrated in Figs. S8 and S9a, b. The responsivity can be calculated by applying the formula *R* = *I*_ph_/*P*_inc_, where *I*_ph_ is the photocurrent density and *P*_inc_ represents the light power density (Table S1). Figure 4c, d show the responsivity of the device across varying incident light angles. Notably, the peak responsivity is observed at 90° for both routes within the front 180° range, whereas the maximum responsivity on the backside is achieved at 270°. When the light is incident vertically, its distribution area is minimized, resulting in the maximum energy per unit area. In contrast, obliquely incident light is distributed over a larger area, causing a decrease in energy per unit area. Ultimately, this leads to the responsivity of the photodetector exhibiting an approximately cosine-like relationship with the incident angle of light. Furthermore, as the incident 365 nm light is absorbed while penetrating the ITO substrate, the responsivity of the vertical backside is lower compared to that of the vertical frontside (Fig. S6). Despite the lower responsivity in the 270° direction, the device maintains effective photoresponse output, indicating quasi-omnidirectional photodetection. Therefore, the directional differences are related to the variations in light energy density at different angles, differences in transmittance between the front and back surfaces, and interference effects arising from the refractive indices of (Al,Ga)N/GaN nanowires and the substrate^46,47^. The device exhibits a weaker response at 0/180° compared to those at other angles, resulting in photodetection blind spots (Fig. 4c, d). More details of the blind spot performance of the device are evaluated in Fig. S9c, d. Additionally, Fig. S10 and Supplementary Note 6 discuss the anisotropic response of the nanowire material.

To further enhance the responsivity of the backside, a substrate with high transparency and good conductivity is indispensable. Decreasing the thickness of ITO film or using Si-doped Ga_2_O_3_ substrate maybe a promising choice^48^, which could be studied in future work. With a dark current of <1 pA and a light-to-dark current ratio exceeding 20 (as illustrated in Figs. S11a and 4e), our results are comparable to, and in some cases superior to, other significant work featuring low dark or photocurrent^49^, demonstrating substantial potential for practical applications. Figures 4f and S11b, c demonstrate that our device possesses a rapid response speed of < 4 ms, which is comparable to the notable achievements reported in the literature^50^. To obtain more specific information on the response speed, we have conducted transient photoresponse tests (Fig. S11d). The rise time is 1.6 μs and the fall time is 21 μs. Moreover, the device has a photovoltaic voltage (*V*_ph_) of 210 mV (Fig. 4g) under 365 nm, facilitating signal amplification and processing^51,52^. The change in barrier height at the graphene/(Al,Ga)N heterojunction interface, from qV_Bi_ in darkness to *q*(*V*_Bi_-*V*_ph_) under illumination (Fig. S12 and Supplementary Note 7), causes the observed rightward shift in the current-voltage curve (Fig. 4g). For a more in-depth exploration of the advantages of utilizing *V*_ph_ in practical applications, refer to Supplementary Note 8. As a result, the integration of a transparent quasi-omnidirectional photodetector into corresponding external circuit systems offers significant convenience and efficiency.

As the (Al,Ga)N/GaN nanowires and new ITO/glass substrate do not absorb visible light, our device exhibits a high UV/visible light rejection ratio. As depicted in Fig. S13, our device boasts a high UV/visible light rejection ratio, enabling it to effortlessly shield against interference from visible light in natural environments during operation, thereby eliminating the need for supplementary filters and simplifying the overall system complexity. Table S2 provides a summary of the most recently reported self-powered photodetectors utilizing GaN-based nanowires, highlighting that our device outperforms most in terms of the spatial resolution, response speed, and UV/visible light rejection ratio. To illustrate the applicability of the dual-mode transparent device under 0 V bias in a more tangible manner, we set a wireless communication system in Fig. 4a. This system is comprised of three key components: a signal encoding module, our 360° quasi-omnidirectional detector, and a signal reading module. The information is encoded according to ASCII code rules and transmitted by controlling the 365 nm LED. The dual-mode transparent device serves as a signal receiver, which is eventually read and analyzed by a data acquisition system. Figure S14 illustrates the varying photoresponse characteristics of the device across different frequencies. Under a 0 V bias, Fig. 4h, i show the combined transmission of the English letters “USTC” at 5 Hz. It should be noted that the large sampling time interval is the reason for the roughness of the curve. Due to the high transparency of our dual-mode device, the stable wireless transmission can be achieved both in the front (90°) and back (270°), demonstrating the potential of our device in lightweight wireless communication equipment. Moreover, the “on/off” cycle measurement in Fig. S15 has proven the stability of the device. In brief, this exceptional quasi-omnidirectional photodetection and self-driven capability render it promising for next-generation low-energy consumption applications.

### Function as a synaptic device

**Fig. 5:**
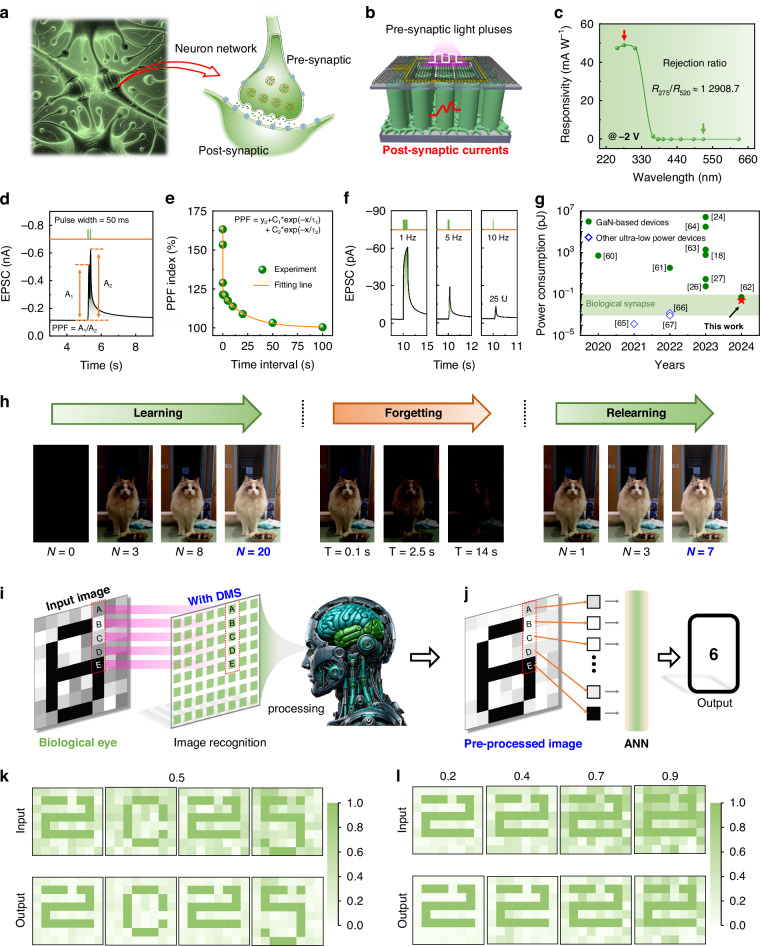
The performance demonstration of the dual-mode transparent device functioning as a synaptic device at 310 nm. **a** Schematic illustration of the neuron cell. **b** Schematic diagram showing the components of the dual-mode transparent device that align with the biological synaptic architecture. **c** The UV/visible light rejection ratio of the device under −2 V bias voltage. **d** EPSC is triggered by two light pulses with a wavelength of 310 nm. **e** *PPF*-time interval curves of the device induced by paired light pulses at −2 V bias voltage. **f** Power consumption of the device under 1, 5, and 10 Hz and 9.2 μW cm^−^^2^ at −0.1 V bias. **g** A summary of the power consumption among various GaN-based synaptic devices. **h** Learning, forgetting, and relearning experience behaviors of the device. **i** Schematic diagram of the structure and function of a human-like visual system, utilizing DMS for noise reduction on input images. **j** Schematic diagram of an image being input into the classic ANN after pre-processing. **k** Comparison of contrast enhancement and noise reduction effects of DMS on the digital image “2025”. **l** The analyses are conducted on the numerical image “2”, both in their raw and pre-processed states under varying degrees of background noise. “Input” denotes the images that have yet to undergo preprocessing, whereas “Output” signifies the images that have undergone noise reduction

After confirming that the dual-mode device can be used as a photodetector, we comprehensively evaluated the synaptic performance of the dual-mode device under negative bias voltage. In the human brain (Fig. 5a), the transmission of visual signals is carried out by the release of neurotransmitters from neuronal cells at the interface between pre-synaptic and post-synaptic terminals^53^. In our simulation system of neuromorphic vision sensing (Fig. 5b), periodic light pulses and dual-mode monolithic devices serve as the analogs of neurotransmitters and postsynaptic receptors, respectively. The wavelength independence of the device is presented in Figs. 5c and S13. UV imaging generally remains unaffected by visible light interference, rendering it exceptionally well-suited for high-brightness environments or low-contrast scenarios, such as target detection against bright backgrounds^54–57^. Supplementary Note 9 offers additional discussions on the utilization of the dual-mode device in the UV range, as well as its broader applications. When a pulsed beam of light strikes the surface of the device (310 nm), light-induced electrons are captured by the positive charge center (V_O_^2+^), thereby triggering the excitatory postsynaptic current (EPSC). Figure 5d illustrates the distinctive EPSC behavior of the device in response to two sequential light pulse stimuli with a 50 ms interval (∆t) under −2 V bias. Upon the initial light pulse, the EPSC attains its peak value (A_1_) and then gradually decreases during the light-off period. Following the second light pulse, a more pronounced peak value (A_2_) is achieved, which can be attributed to the heightened accumulation of photogenerated carriers within the transmission channel during the subsequent light pulse. It mainly results from the sluggish relaxation of carriers trapped by V_O_^2+^ during the light-off phase. The phenomenon of paired-pulse facilitation (*PPF*) is routinely utilized to gain deeper insights into the enhancement potential (or weight) of synaptic transmission between two consecutive light stimuli. This enhancement can be mathematically quantified by employing the following formula^27^:

2 $$P{PF}={{\rm{A}}}_{2}/{{\rm{A}}}_{1}\times 100 \%$$

A double exponential function is utilized to predict the synaptic response dynamics exhibited by the device^31^:

3 $${PPF}\,{\rm{index}}={C}_{0}+{C}_{1}{{\rm{e}}}^{-\frac{\Delta t}{{\tau }_{1}}}+{C}_{2}{{\rm{e}}}^{-\frac{\Delta t}{{\tau }_{2}}}$$

*C*_0_, *C*_1_, and *C*_2_ are the initial facilitation magnitudes, and *τ*_1_/*τ*_2_ are related to the characteristic relaxation times of rapid/slow phases, respectively. As illustrated in Fig. 5e, it becomes apparent that as the time interval ∆t increases from 0.05 to 100 s, the *PPF* index decreases. That means the amplifying effect of the second stimulus diminishes, indicating a decrease in synaptic weight. This decreasing trend aligns with biological synaptic learning patterns, accurately mirroring the strength of synaptic connections between neurons^58^. Inspired by the bipolar photoresponse^20^, the creation of a double heterojunction (e.g., a bidirectional built-in electric field in Fig. S16) in nanowires through band engineering and doping techniques offers the potential to simulate both excitatory and inhibitory synaptic functions in dual-terminal devices.

Power consumption (*E*_UI_) serves as another crucial parameter for synaptic devices, which can be calculated using the equation^59^:

4 $${E}_{{UI}}={I}_{{\rm{peak}}}\times V\times T={\int }_{{t}_{0}}^{{t}_{1}}V\cdot I\left(t\right){dt}$$

Here, *I*_peak_ represents the maximum photocurrent, *V* stands for the applied bias voltage, and *T* denotes the duration of a single event. Figure S17 shows the calculation details. Owing to its switchable operation mode design, the *E* of the device can be as low as 25 fJ (Fig. 5f, g), which is already comparable to that of the human brain (ranging from 1 to 100 fJ). Operation at the lower voltages may further reduce *E*, making it an ideal neuromorphic device. As summarized in Table S3, the *E* attained by our device stands as one of the lowest values among GaN-based synaptic devices and is comparable to those of ultra-low power devices based on other materials^18,24,26,27,39,60–67^. Since the power of the light source is crucial for practical applications, Supplementary Note 10 and Table S3 offer further discussions and comparisons.

Biologically, the human brain typically requires less time to relearn forgotten information than it does to acquire it initially. Figures 5h and S18 illustrates the learning, forgetting, and relearning visualization processes of light-simulated synapses. The initial learning phase takes 20 s, while the subsequent relearning phase requires just 7 s, revealing the classical behavior of learning and experience. The EPSC behavior of the dual-mode device can be adjusted by varying the number of incident light pulses and the intensities of the light. As can be seen from Fig. S19a, an increase in the number of light pulses (Num.) elicits stronger EPSCs, demonstrating a transition from short-term plasticity (STP) to LTP. Additionally, an increase in optical power density from 45.6 to 84.2 μW cm^−^^2^ also results in enhanced EPSCs and a shift from STP to LTP (Fig. S19b). The transition from STP to LTP is discussed in Fig. S19c–e and Supplementary Note 11. Since the neuromorphic vision system (NVS) operates in an event-driven manner, it triggers signal transmission only when changes in photons are detected^68^. It is different from traditional imagers that require periodic scanning of the entire scene, significantly reducing redundant data processing and enhancing information processing efficiency. Because NVS only transmits and processes data when required, it consumes significantly less energy compared to traditional imagers such as complementary metal oxide semiconductor and charge-coupled devices^69^. The advantage and necessity of synaptic device arrays stem from their exceptional flexibility and adaptability, rendering them ideal for intricate and varied noise reduction tasks as well as applications in intelligent devices^18,69^. As outlined in Supplementary Note 12, the utilization of synaptic devices facilitates image preprocessing directly on the hardware level, enabling the parallel computation of extensive pixel information. This is similar to the visual cortex of the human brain, which saves energy by selectively processing important visual information (Fig. 5i). Therefore, by combining bionics and computational neuroscience technologies, it is possible to mimic the structures and functions of the visual cortex in the human brain, such as noise processing and contrast enhancement of images. In Fig. 5i, a hypothetical arrangement of 8 × 8 dual-mode synaptic devices (DMS) array is employed to demonstrate the concept of simulating the sensing and preprocessing functions executed by biological vision systems, with the aim of achieving image denoising and contrast enhancement. Figure S20a presents the structure of the device array. Figure S20b illustrates the response uniformity of the devices under identical illumination conditions, and Fig. S20c presents the variations among devices from different batches. The random background noise of the images is generated by light signals of different intensities, which are normalized in the range of 0 to 1. The correlation between the EPSC of the DMS and the intensity of the input light is illustrated through a fitted curve and then encoded by machine language (Fig. S21). The nonlinearity effect of EPSC is intimately tied to the dynamic equilibrium among carrier generation, surface defect filling, and V_O_ ionization within the device^18,69^. Supplementary Note 13 and Fig. S22 provides detailed information about the nonlinear response and its characteristics.

As a proof-of-concept experiment, we have encoded the grayscale values of these pixel blocks based on five levels of light intensity to demonstrate the contrast enhancement function of the image (Figs. 5i, j and S23). During the visualization phase aimed at noise reduction and contrast enhancement, 5-pixel blocks are extracted from a designated area within the digital image. The regions are labeled as A, B, C, D, and E. As shown in Fig. S21 and Table S4, the smallest P_1_ normalized to 0 and the largest P_5_ normalized to 1. As depicted in Figs. 5i, S19, and S23 and Table S5, the light intensities for regions A, B, C, D, and E correspond to 62.4 μW cm^−^^2^, 49.8 μW cm^−^^2^, 55 μW cm^−^^2^, 63.6 μW cm^−2^ and 84.2 μW cm^−2^, respectively. Consequently, the normalized values for the five pixels A, B, C, D, and E are 0.434, 0.108, 0.243, 0.465 and 1, respectively. Following pre-processing by the DMS, the output EPSC values for pixels A, B, C, D, and E are recorded as 0.177, 0.034, 0.079, 0.198, and 1, respectively. When compared to the normalized light intensity in the input image, a notable amplification in the normalized current difference is observed between output pixels A, B, C, D, and E, resulting in an enhancement of the image contrast. Figure 5k depicts the noisy input images displaying the digits “2025” according to this rule, where the noise is applied using random noise values ranging from 0 to 0.5. Upon preprocessing via the DMS, the prominent characteristics of the handwritten digit images are emphasized, while the background noise signals undergo smoothing. A more in-depth discussion on the dependability of the simulation outcomes refers to Supplementary Note 14.

To more clearly contrast the role of DMS in the process of image denoising, the processed and unprocessed images are subsequently fed into the tailored artificial neural network (ANN) for the execution of image recognition tasks (Figs. S23 and 5j). The construction of an ANN involves multiple steps, including data preparation, model design, training and optimization. The details of the construction are described in the paragraphs preceding Fig. S23a. Supplementary Notes 13 and 15 describe how to use equipment to control the display of unprocessed images in practical operations. Figure S23b illustrates the principle of image preprocessing using a DMS array. Furthermore, in practical applications, we can utilize a trans-impedance amplifier (TIA) with high input impedance and low noise to convert low-level current signals into measurable voltage signals as discussed in Supplementary Note 16. In the established ANN simulation system, both pre-processed and unprocessed image data serve as inputs for the image training and recognition procedures. Figure S23c shows the recognition accuracy of visual systems with and without the application of DMS, highlighting substantial improvements in both accuracy and efficiency attributable to the image preprocessing step. The confusion matrix statistics presented in Fig. S23d, e demonstrate the recognition accuracy before and after preprocessing over 7500 epochs. The predicted output digits in each row align well with the expected output digits in each column, indicating correct inference for each digit from 0 to 9. Furthermore, the DMS demonstrates remarkable noise reduction and feature enhancement capabilities across various noise levels (Figs. 5l and S24). The image recognition accuracy is notably higher after preprocessing. Table S6 compares the power consumption of the dual-mode device during preprocessing with those of other classic devices, revealing that the DMS exhibits relatively low consumption. In summary, by mimicking the working mechanism of the visual cortex in the human brain through bionic principles, the DMS provides an efficient, low-power and fast-response solution for visual information processing. It holds promising future applications in various fields such as autonomous driving, robotic vision, and intelligent surveillance.

### Transparent dual-mode device for humanoid robot learning

**Fig. 6:**
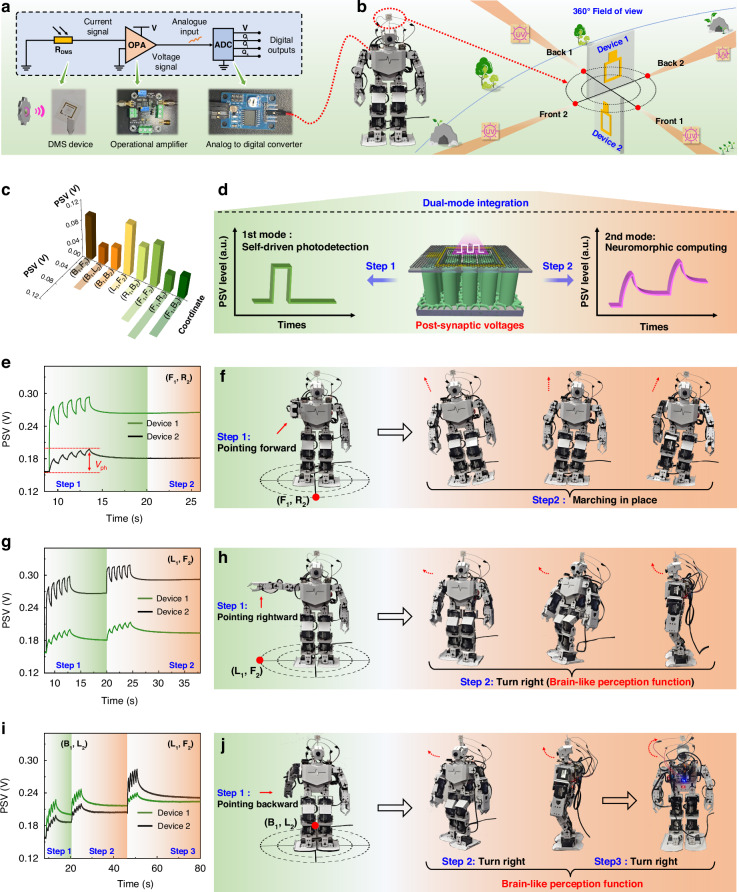
Motion learning of humanoid intelligent robots under optical stimulation at 0 A bias (voltage mode). **a** Schematic representation of the control system for a humanoid robot, along with physical depictions of an operational amplifier (OPA) and an analog-to-digital converter (ADC). **b** Schematic diagram of the wide field of view for detection in the UV range using two transparent dual-mode devices, which are placed orthogonally. Define the front, back, left, and right of Device 1 as F_1_, B_1_, L_1_, and R_1_, respectively, and similarly define Device 2 (F_2_, B_2_, L_2_, and R_2_). **c** PSV values at eight coordinate positions. Optical information (1 Hz, 6.4 mW cm^−2^) controls two dual-mode devices to distinguish incident light direction and steer robotic movements, simulating learning and memory capabilities. **d** Schematic diagram of dual-mode integration, where step 1 represents the function of self-driven photodetection and step 2 represents the function of neuromorphic computing. **e** The light source is placed on the front side of the robot (F_1_, R_2_). **f** The mechanical arm of the robot points forward (step 1) and then performs a marching-in-place motion (step 2) due to no second light stimulus being received after more than 20 s. **g** The light source is placed on the right side of the robot (L_1_, F_2_). **h** The mechanical arm of the robot points to the right (step 1), and then performs a right turn motion (step 2) under second light simulate. **i** The light source is placed on the back side of the robot (B_1_, L_2_). **j** The mechanical arm of the robot points backward (step 1), then performs a right turn motion upon the second light stimulus (step 2), and performs another right turn motion upon the third light stimulus (step 3)

In practical applications, signal reading relies on precisely designed equipment. *V*_ph_ refers to the voltage across the device when the circuit is open, reflecting the potential difference caused by the accumulated charges at both ends after the separation of photogenerated carriers^70,71^. Under illumination, the redistribution of carrier density leads to the separation of quasi-Fermi levels in the n-region and p-region. The *V*_ph_ is actually the difference between the quasi-Fermi levels, which can be significant even when the photocurrent is small. Therefore, by detecting the *V*_ph_ and combining it with the transparent characteristics of the device, we have ultra-wide field-of-view detection capability for UV light (Fig. 6a, b). Figure S25 provides a detailed illustration of the differences between the current mode and voltage mode. In voltage mode, the synaptic plasticity mechanism involves the accumulation of photogenerated carriers facilitated by *V*_ph_ and the dynamic relaxation of V_O_^2+^ regulated by the internal field. Figure S26a illustrates the capability for long-term memory retention. Figure S26b, c depict the memory characteristics under varying light intensities and frequencies, respectively. The stable response observed at 48 Hz indicates the potential of this device in high-speed neuromorphic computing applications. The results of timing switching experiments in Fig. S27 demonstrate that the device can rapidly switch from the synaptic state to the angle detection state. Typically, the *V*_ph_ signal is also known as postsynaptic voltage (PSV) in artificial synaptic devices^71^. In brief, the dual-mode device operating under open-circuit voltage test conditions (0 A bias, voltage mode) possesses the capability to perceive and process light stimuli, as well as to attain gradual learning ability.

In Fig. 6a, b, the device constructed from graphene/(Al,Ga)N/GaN heterojunction nanowires is interconnected with the voltage monitoring unit as the light detection part of the learning system. The humanoid robot performs feedback-based mechanical movements in response to signals detected by the module. To demonstrate the motor learning and direction recognition capabilities, two dual-mode devices are orthogonally placed and mounted with the monitoring units on the head of the humanoid robot in Fig. 6b. The connection details of each component of the humanoid robot system are illustrated in Figs. 6a and S28a, and the “Materials and Methods” section. In the first stage, the two dual-mode devices placed orthogonally serve to detect light stimuli. Meanwhile, the PSV signal emitted by these devices is measured and processed by the operational amplifier and analog-to-digital converter module, which then forwards the signals to the computer for further analysis and processing. Python programming is used to distinguish directions or control the movements of the humanoid robot, aiming at executing and adjusting various operational parameters of the robot based on control commands from the synaptic devices. As shown in Fig. 6b, the orthogonal devices can be divided into 8 directions on a plane, where we define the front, back, left, and right of the Device 1 as F_1_, B_1_, L_1_, and R_1_, respectively. The front, back, left, and right of the Device 2 are defined as F_2_, B_2_, L_2_, and R_2_, respectively. Therefore, as shown in Fig. S29, we can obtain coordinates for 8 directions: (B_1_, F_2_), (B_1_, L_2_), (B_1_, B_2_), (L_1_, F_2_), (R_1_, B_2_), (F_1_, F_2_), (F_1_, R_2_), and (F_1_, B_2_). It is worth noting that this is a preliminary exploration of distinguishing direction based on transparent devices. Tests on the PSV of the orthogonal devices can be conducted in these 8 directions, with the test results shown in Figs. 6c and S29. Obviously, we can distinguish the incident direction of light by calculating the PSV values of Devices 1 and 2, as well as their ratios.

To further achieve motor learning, we present a feedback-based motor response leveraging optical modulation informed by neuromorphic behavior, encompassing both learning and memory (Fig. 6d). In our demonstrations, we placed light sources in front of, to the right of, and behind the humanoid robot (Fig. S28b–d and Videos S1). We define the light signal as consisting of a sequence of 1 Hz light pulses, with five pulses comprising one stimulation signal. As shown in Fig. 6e, when the incident light is directly in front of the robot (F_1_, R_2_), Device 1 exhibits a *V*_ph_ of 138 mV, while Device 2 exhibits 43 mV. Figure 6f illustrates the feedback action of the robot, where the robotic arm points forward (step 1, detection stage). When no new light stimulation is received after 20 s, the robot triggers a step-in-place action (step 2, learning stage). When the incident light is to the right of the robot (L_1_, F_2_), Device 1 exhibits 42 mV, and Device 2 exhibits 139 mV (Fig. 6g). In this scenario, as shown in Fig. 6h, the robotic arm points to the right (step 1, detection stage). Upon receiving a second light stimulation signal after 20 s, the robot triggers an action of turning right (step 2, learning stage). When the incident light is behind the robot (B_1_, L_2_), as illustrated in Fig. 6i, j, Device 1 exhibits 71 mV, and Device 2 exhibits 42 mV. At this point, the feedback action of robot is for the robotic arm to point backward (step 1, detection stage). Upon receiving a second light stimulation signal after 20 s, the robot triggers an action of turning right (step 2, learning stage). After completing this action, the orientation of the orthogonally placed devices also changes accordingly. At this time, the front of Device 2 faces the direction of light source F_2_, while the left side of Device 1 faces the direction of light source L_1_, so the coordinates are (L_1_, F_2_). After receiving a third light stimulation signal, the *V*_ph_ increment value exhibited by Device 2 is larger than that of device 1, prompting the robot to trigger another action of turning right, i.e., a turn backward (step 3).

The aforementioned results demonstrate the practical application potential of dual-mode devices in the field of motor learning, especially considering that the devices themselves require no applied bias voltage, which can reduce the overall energy consumption of the system. Figure S30a displays a 3 × 3 DMS array that exhibits a distinct “T”-shaped image after stimulation with five 1 Hz pulses of 365 nm light. When we recorded the PSV values at the 5 s and 15 s after turning off the light, they remained at a high level (Fig. S30b–e), demonstrating a prolonged “non-volatile” response characteristic. In the future, with continuous optimization of system integration and algorithms, DMS devices will play a pivotal role in realizing true brain-like intelligence, autonomous learning and imaging systems.

## Discussion

In summary, we have presented a dual-mode switchable device that seamlessly integrates a self-driven photodetector with an artificial synapse. At a bias voltage of 0 V, the device exhibits rapid response time with a minimal number of carriers engaged in V_O_ ionization. Given the high transmittance of the (Al,Ga)N nanowire film, it effectively functions as a 360° quasi-omnidirectional photodetector. Furthermore, the dual-mode device demonstrates stable optical communication capabilities in both front and back directions. In contrast, the application of bias voltage effectively suppresses carrier recombination, enabling a larger number of charges within the nanowires to facilitate the ionization of V_O_ into V_O_^2+^, ultimately leading to the observed PPC behavior. Due to the elimination of the influence of the Si substrate, our device exhibits a high UV/visible light rejection ratio of up to 1.29 × 10^4^. The dual-mode device exhibited characteristics akin to an artificial synapse, including the properties like *PPF*, dynamic transition from STP to LTP, and learning and forgetting behaviors. Importantly, via bias voltage and frequency regulation, the power consumption of a synaptic event can be reduced to only 2.5 × 10^−^^14^ J. Specifically, the contrast of handwritten digit images is significantly enhanced after DMS preprocessing through nonlinear programmable logic encoding of input light intensity. The improvement in recognition accuracy also demonstrates the promising application prospects of our device in image recognition. Notably, by integrating a dual-mode device with a humanoid robot and detecting PSV signals from two orthogonally positioned devices, the system can distinguish movement in eight different directions. Furthermore, leveraging the LTP in response to light signals, we effectively implemented motion control for the humanoid robot, enabling it to perform different tasks.

## Materials and methods

### MBE growth of nanowires

The (Al,Ga)N nanowires were prepared by plasma-assisted molecular beam epitaxy (MBE, Vecco G20) based on n-type Si (111) substrates. First, we heated the Si substrate at 900 °C for 15 min to remove native oxides. After that, a 3 nm thick AlN sacrificial layer was grown at 830 °C. Subsequently, the bottom GaN segment was grown for 300 min with a Ga flux of ~4.0 × 10^−8^ Torr. Then (Al,Ga)N segment was grown for around 60 min in total with a nominal Al/Ga flux ratio of ~ 0.75, in which the Al and Ga atoms were generated by the Al and Ga effusion cells, respectively. The N atoms were supplied by the N plasma cell. The nitrogen flow rate and plasma power were kept at 4.8 sccm and 450 W, respectively.

### Preparation of transparent photodetector/artificial synapse switchable device

First, the as-grown Si-based (Al,Ga)N nanowires sample was divided into small pieces for electrochemical detaching (Fig. 1d). The detailed lift-off and transfer process can be found in our previous work^8,42^. After this, a 400 nm SiO_2_ insulating layer was coated on the surface of the whole sample. Thereafter, the lithography and reactive ion etching processes leave a square hole in the SiO_2_ layer to expose the nanowire array (Fig. 1d). The effective area of the device is 4 × 10^−4^ cm^2^. Subsequently, the top Ti/Au (20/200 nm) electrode was prepared by the second lithography process and electron beam evaporation. Lastly, the graphene was transferred onto the top segment of the nanowires array to prepare the current-collecting layer after the corrosion of primary copper substrates.

### Connection between the device and humanoid robot

Two dual-mode devices are orthogonally placed, connected using epoxy resin, and then fixed onto the head of the robot with hot glue. The positive and negative terminals of both devices are connected to a voltage signal detection circuit board integrated at the back of the robot via wires. To demonstrate the neuromorphic functionality of the devices, we designed a simple experiment where the humanoid robot recognizes the direction of incident light and provides corresponding “action” feedback for verification. In the entire system, our devices are used to detect light signals, analyze the detected photovoltaic voltage signals, and perform action demonstrations based on the analysis results. The actions of the robot are controlled through Python programming. During the demonstration, the light source (365 nm LED) is placed in front of, to the right of, and behind the robot through a stand. We set the position of the humanoid robot as (0,0), with the front, back, left, and right directions of Device 1 designated as F_1_, B_1_, L_1_, R_1_, respectively, and those of Device 2 as F_2_, B_2_, L_2_, R_2_, respectively. Based on the orthogonal relationship between the two devices, the front of the robot/Device 1 is coordinated as (F_1_, R_2_), the back as (B_1_, L_2_), the right side of the robot/front of Device 2 as (L_1_, F_2_), and its back as (R_1_, B_2_).

### Characterization and measurements

The vertical arrangement and random distribution of nanowires were observed at 3 kV by scanning electron microscopy (SEM, S-4800, HITACHI). Detailed morphologies, element distribution, and atomic arrangement of the nanowires were examined via spherical-aberration-corrected scanning transmission electron microscopy (AC-STEM, Themis Z, FEI) at 200 kV. Focused ion beam (Scios, FEI) was used to prepare the sample for STEM characterization. The chemical states and atomic binding were characterized by X-ray photoelectron spectroscopy (XPS, PHI 5000 Versa probe III, ULVAC-PHI). Room-temperature photoluminescence test was performed using 325 nm lasers (LABRAM HR). Spectrophotometer (LAMBDA 750) was utilized to measure the transmittance of the device. Light sources of 310 and 365 nm LEDs were controlled by a function generator (FY6900-20M). Current-voltage/time measurements were performed by a semiconductor device analyzer (B1500A).

## Supplementary information

Video S1

Supplementary material

## Data Availability

The data that support the findings of this study are available from the corresponding authors upon reasonable request.
