# Supplementary material for: A dual-mode transparent device for 360° quasi-omnidirectional self-driven photodetection and efficient ultralow-power neuromorphic computing
Source: Light Sci Appl. 2025 Aug 12;14:273. doi: 10.1038/s41377-025-01991-y (PMC12343816; doi:10.1038/s41377-025-01991-y)
Supplement: Supplementary file 2 — Supplementary material [file 41377_2025_1991_MOESM2_ESM.docx]

Supplementary Information for

A dual-mode transparent device for 360° quasi-omnidirectional self-driven photodetection and efficient ultralow-power neuromorphic computing

Min Jiang,^1,2^ Yukun Zhao,^1-3*^ Tong Liu,^4^ Yanyan Chang,^2^ Yuan Tang,^5^ Min Zhou,^2^ Yiping Shi,^5^ Jianya Zhang,^5*^ Lifeng Bian,^6*^ Shulong Lu^1,2*^

^1^ School of Nano-Tech and Nano-Bionics, University of Science and Technology of China (USTC), 230026 Hefei, China.

^2^ Division of Nano-Devices Research, Suzhou Institute of Nano-Tech and Nano-Bionics (SINANO), Chinese Academy of Sciences (CAS), 215123 Suzhou, China.

^3^ Key Laboratory of Semiconductor Display Materials and Chips, SINANO, CAS, 215123 Suzhou, China.

^4^ Vacuum Interconnected Nanotech Workstation, SINANO, CAS, 215123 Suzhou, China.

^5^ Key Laboratory of Intelligent Optoelectronic Devices and Chips of Jiangsu Higher Education Institutions, School of Physical Science and Technology, Suzhou University of Science and Technology, 215009 Suzhou, China.

^6^ Frontier Institute of Chip and System, Fudan University, 200433 Shanghai, China.

**Email:** ykzhao2017@sinano.ac.cn; [jyzhang2022@usts.edu.cn](mailto:jyzhang2022@usts.edu.cn); [lfbian@fudan.edu.cn](mailto:lfbian@fudan.edu.cn); [sllu2008@sinano.ac.cn](mailto:sllu2008@sinano.ac.cn)

**The PDF file includes:**

Figure S1 to S30

Table S1 to S6

Supplementary Text


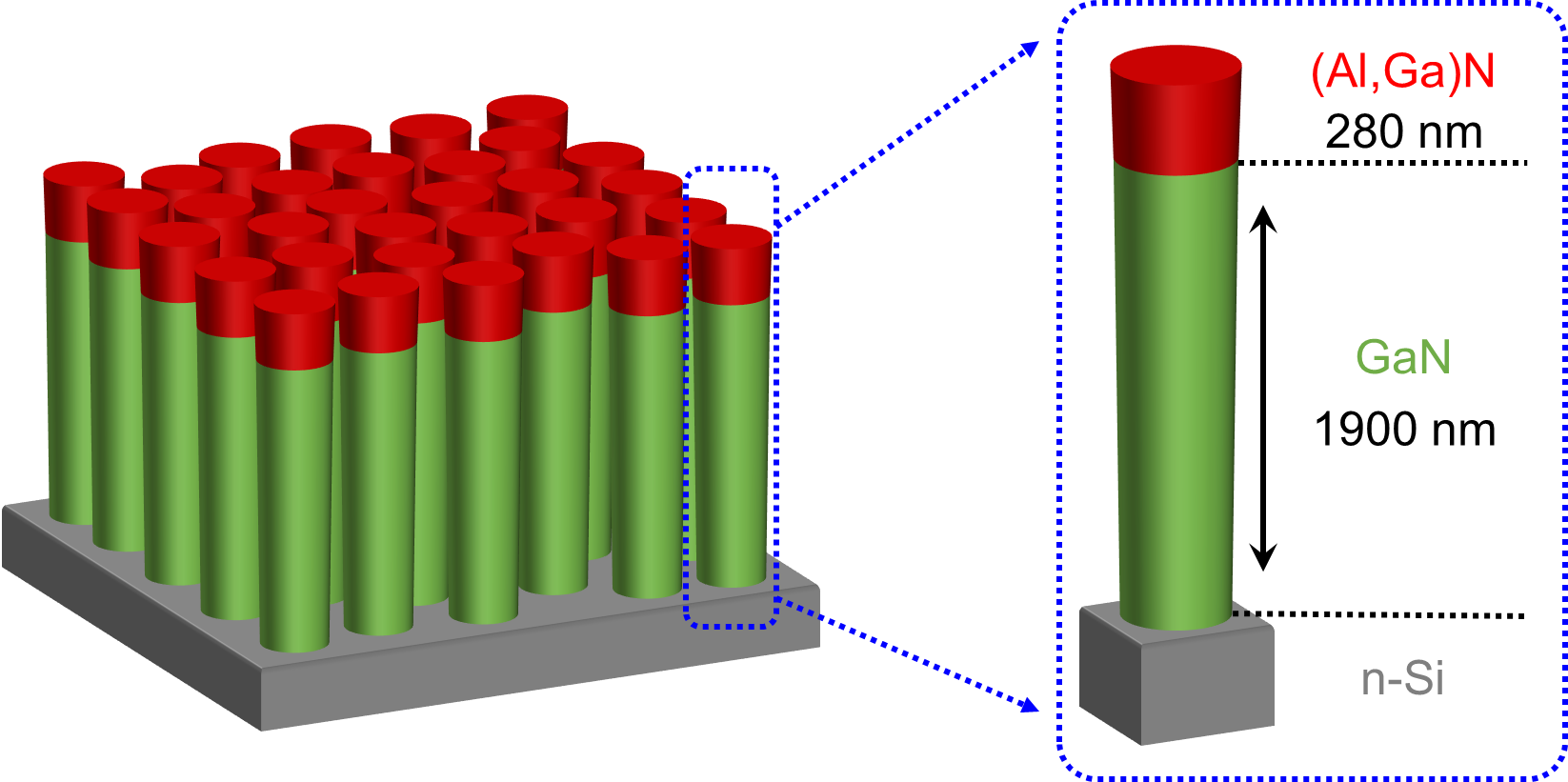


**Figure S1.** A schematic illustration of vertical (Al,Ga)N nanowires (NWs) grown on a planar n-type Si substrate. Each nanowire comprises a top (Al,Ga)N segment and a bottom GaN segment.


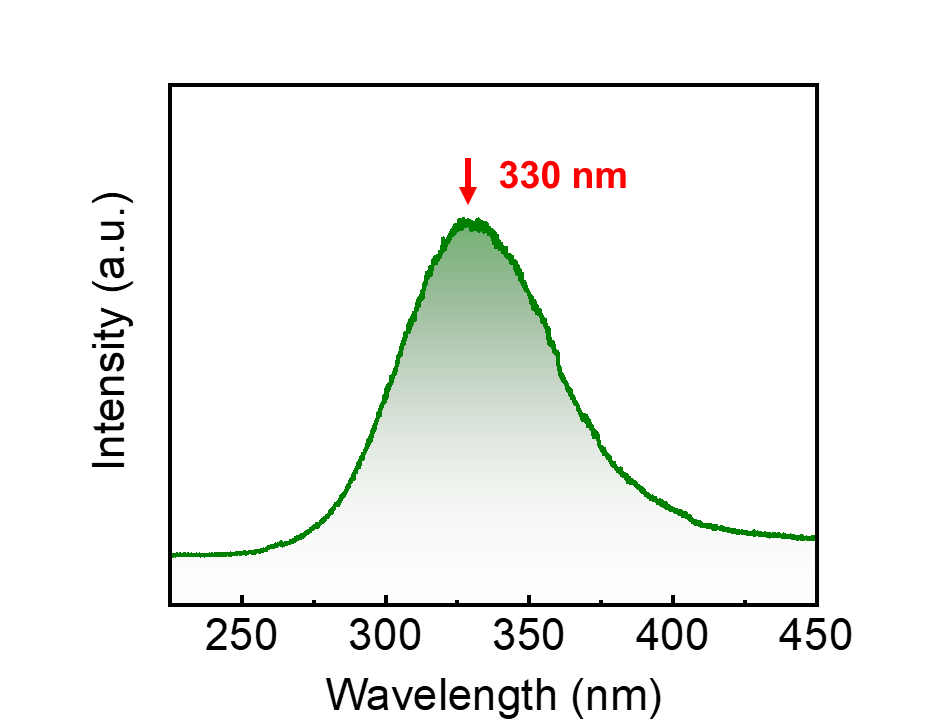


**Figure S2.** Room-temperature photoluminescence (PL) curve of the as-grown (Al,Ga)N/GaN nanowires.


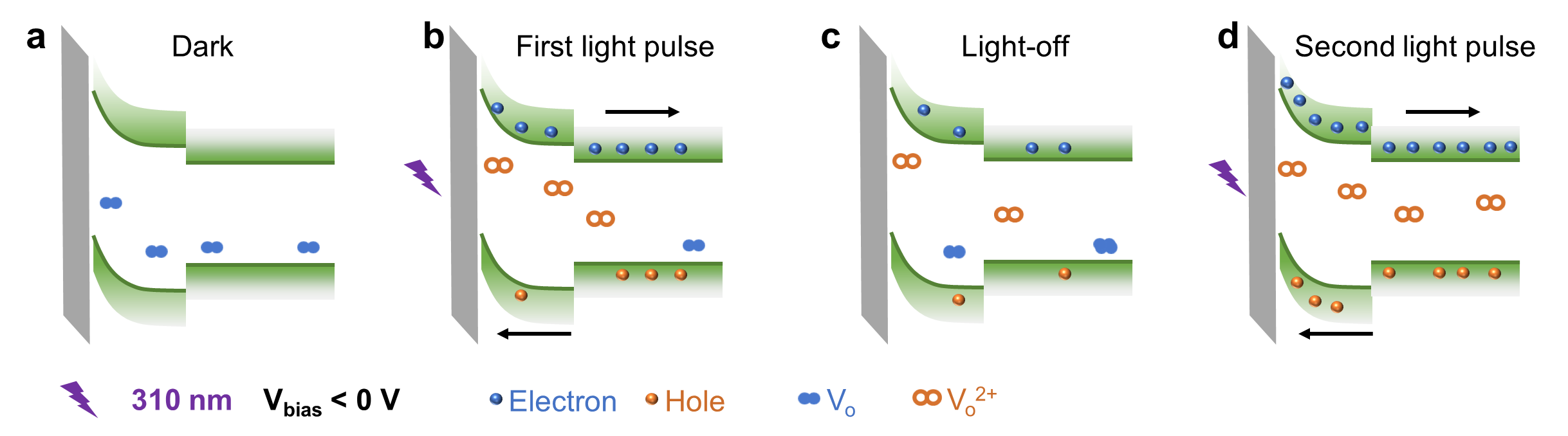


**Figure S3.** Schematic diagram of the process of the persistent photoconductive (PPC) effect in (Al,Ga)N/GaN nanowires under bias voltage (*e.g.*, V_bias_ < 0). (a) In the dark state, there are no carriers, and the oxygen vacancies (V_O_) in the nanowires are not ionized. As an example, there are four V_O_ here. (b) Under illumination of the first light pulse, some V_O_ are ionized to V_O_^2+^. As an example, 7 electrons-hole pairs are generated, and 4 holes are left over after V_O_ ionization. At this point, the number of V_O_^2+^ is 3. (c) After turning off the light, some of the V_O_ remain ionized, allowing the carriers in the nanowires to transport or recombine slowly. As an example, 3 electrons recombine with holes, and one V_O_^2+^ is deionized. There are still 4 electrons and 2 holes left. At this point, the number of V_O_^2+^ is 2. (d) Under illumination of the second light pulse, more V_O_ are ionized to V_O_^2+^, resulting in the higher excitatory postsynaptic current (EPSC). As an example, 7 additional electrons are introduced, with two holes participating in the ionization of V_O_, thereby creating 5 new holes and resulting in a final state of 11 electrons and 7 holes. At this point, the number of V_O_^2+^ is 4.


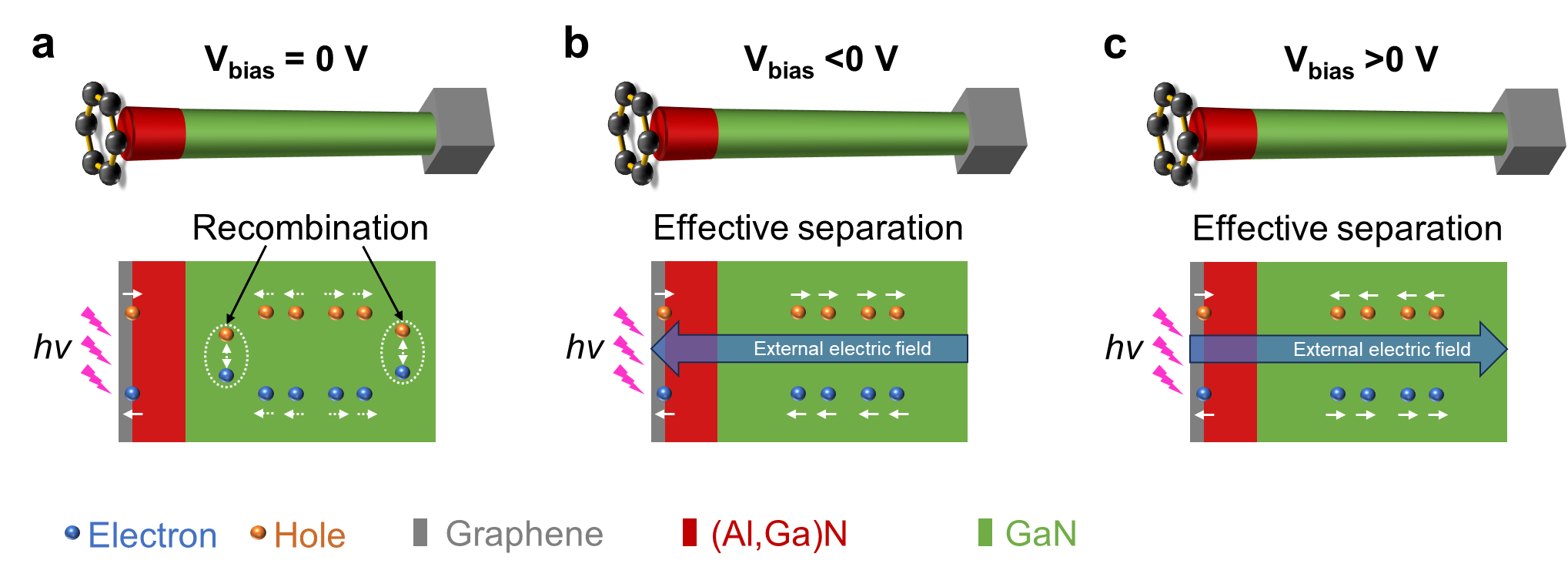


**Figure S4.** The dynamic changes in carrier transport under bias voltages of (a) 0 V, (b) <0 V, and (c) >0 V. It is worth mentioning that, to make the process clearer, we have simplified the carrier transport region inside the nanowire into graphene/(Al,Ga)N heterojunction regions and non-heterojunction regions. Here, we consider the scenario where the external electric field induced by the bias voltage (whether less than or greater than 0 V) plays a dominant role.


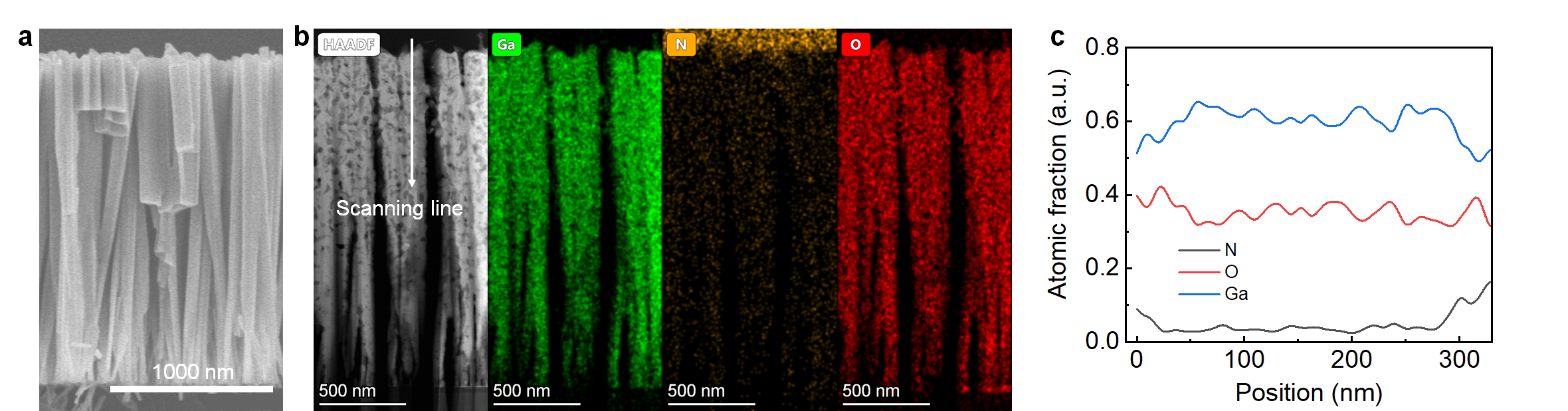


**Figure S5.** (a) SEM image of GaN nanowires without high-temperature annealing. (b) Scanning transmission electron microscope (STEM) images of GaN nanowires subjected to high-temperature oxidation (Environment: O_2_ atmosphere, 1000°C, 10 minutes) and their corresponding energy dispersive spectroscopy (EDS) mapping of gallium (Ga), nitrogen (N) and oxygen (O) elements, along with the corresponding (c) atomic proportion distribution maps. It is noteworthy that under extreme high-temperature conditions, the proportion of N elements drops sharply, and the GaN nanowires are almost entirely oxidized (GaOx), demonstrating the tunability of oxygen concentration.


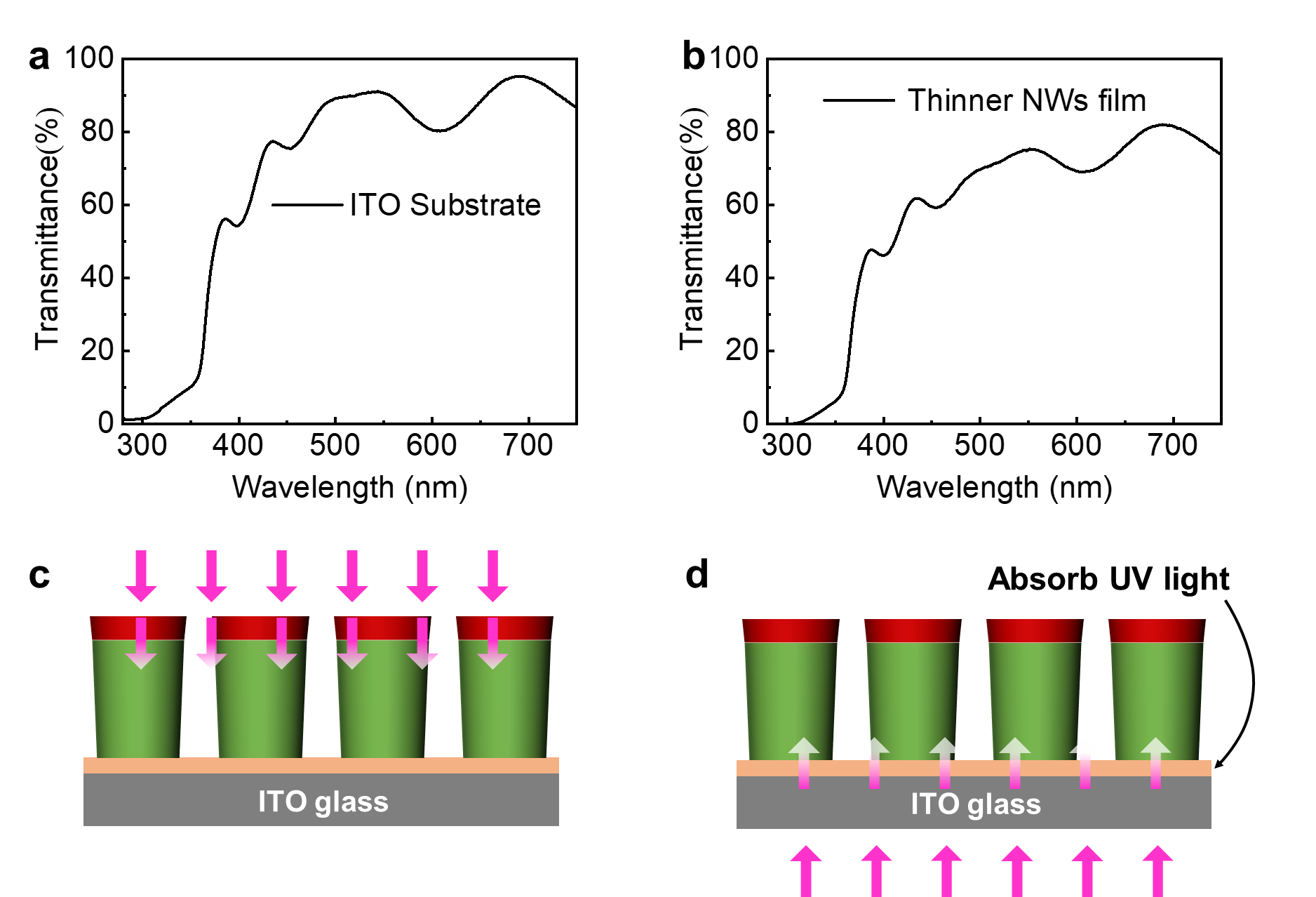


**Figure S6.** Transmittance of (a) ITO substrate and (b) other thinner (AI,Ga)N nanowire materials. The ITO substrate exhibits strong light absorption in the ultraviolet (UV) wavelength range. Schematic diagrams illustrate the absorption characteristics of the detector for light incident from (c) the front (90°) and (d) the back side (270°). When light is incident from the front, the nanowire array can absorb it, whereas when light is incident from the back, a considerable portion of the UV light is absorbed by the underlying ITO substrate, leading to a reduction in responsivity. Furthermore, there exists a refractive index difference between the (AI,Ga)N/GaN nanowires and the ITO substrate, and the interference effect arising from this multilayer structure cannot be ignored.


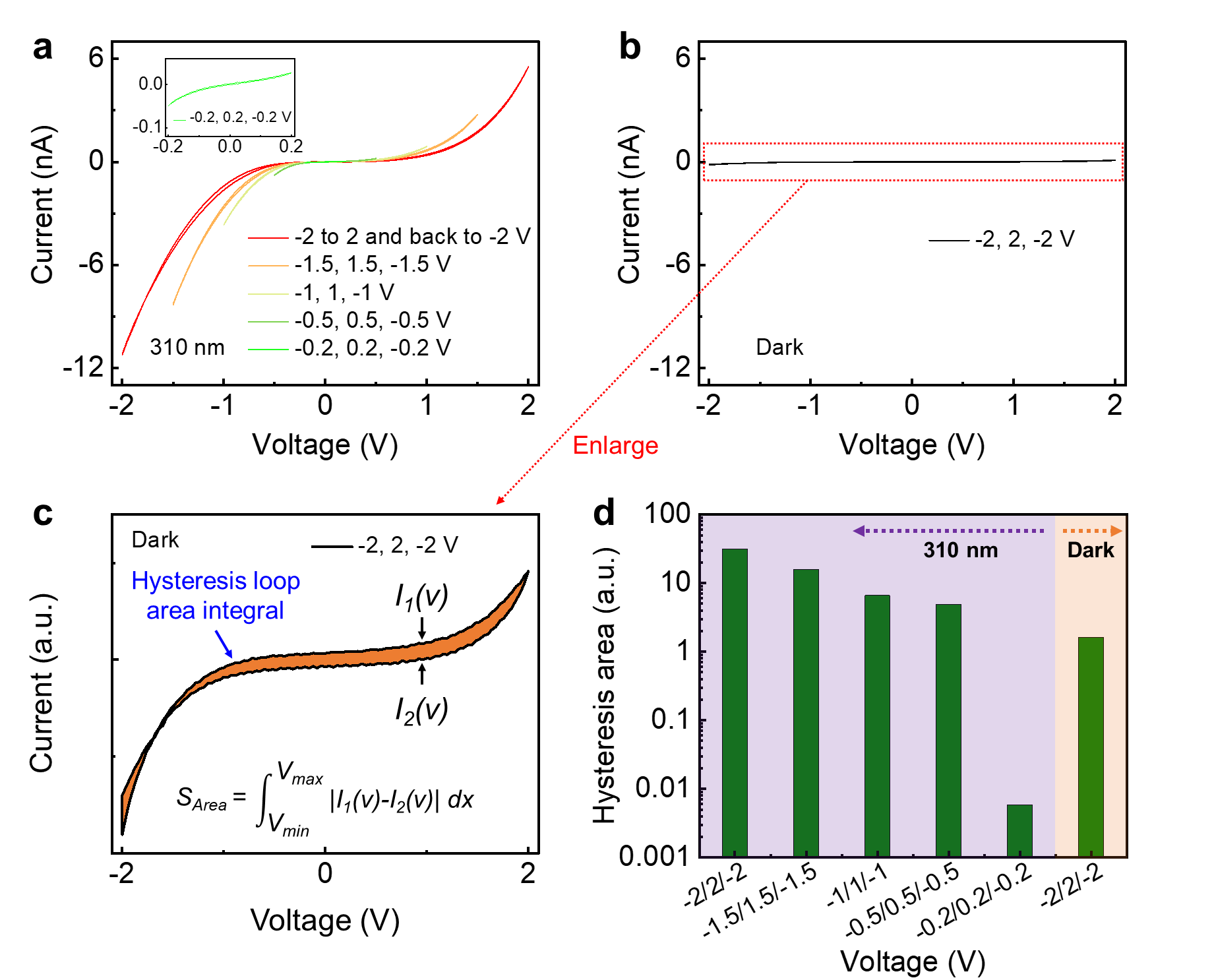


**Figure S7.** I-V hysteresis curves under different bias ranges under (a) 310 nm illumination, and (b) dark conditions. (c) Diagram illustrating the calculation of hysteresis area integral (The enlarged view of Figure S7b). (d) Comparison of hysteresis areas.


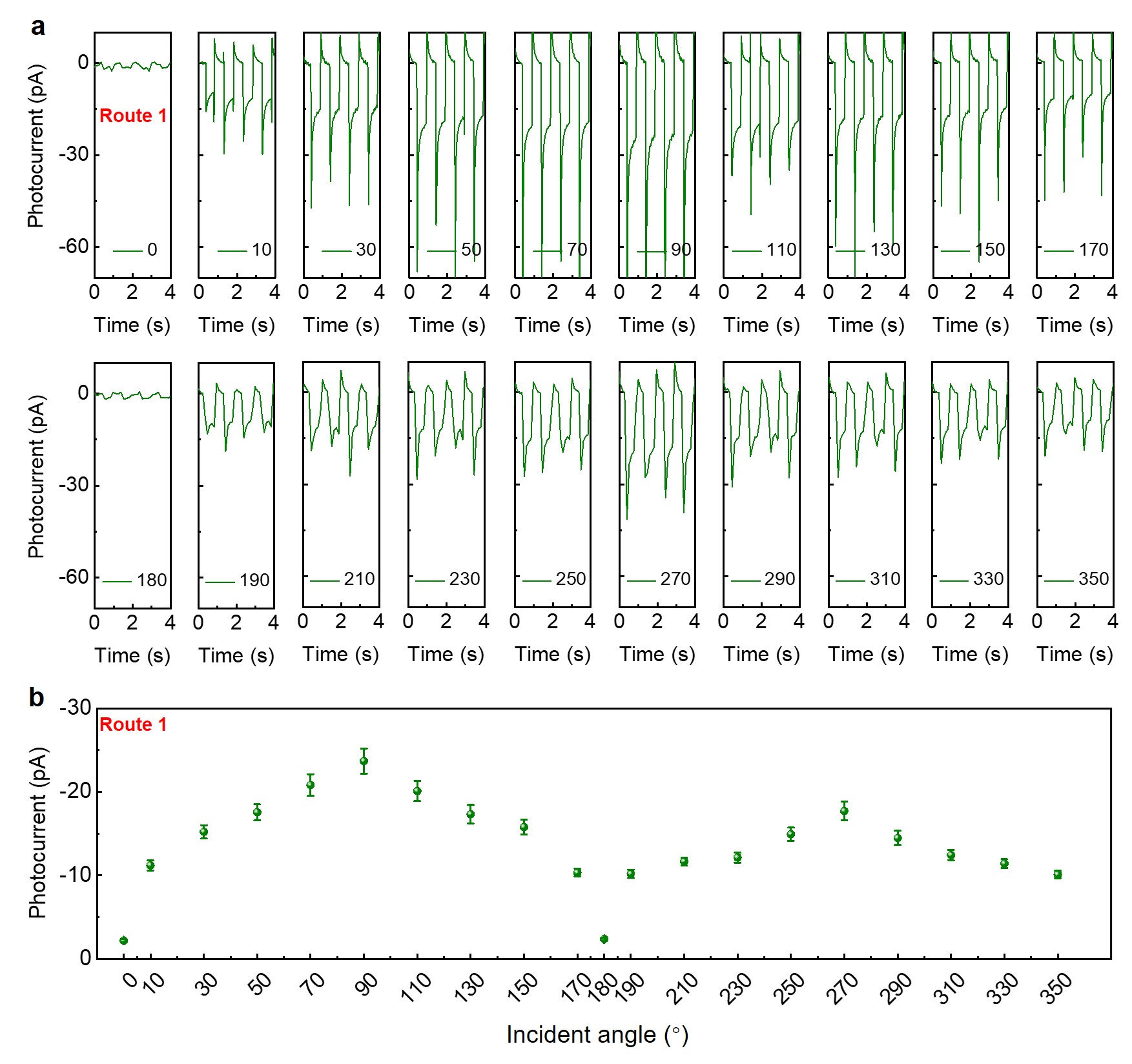


**Figure S8.** (a) The photoresponse and (b) the summarized photocurrent (*I*_ph_) of the device as a function of the angle of incident light along with route 1.


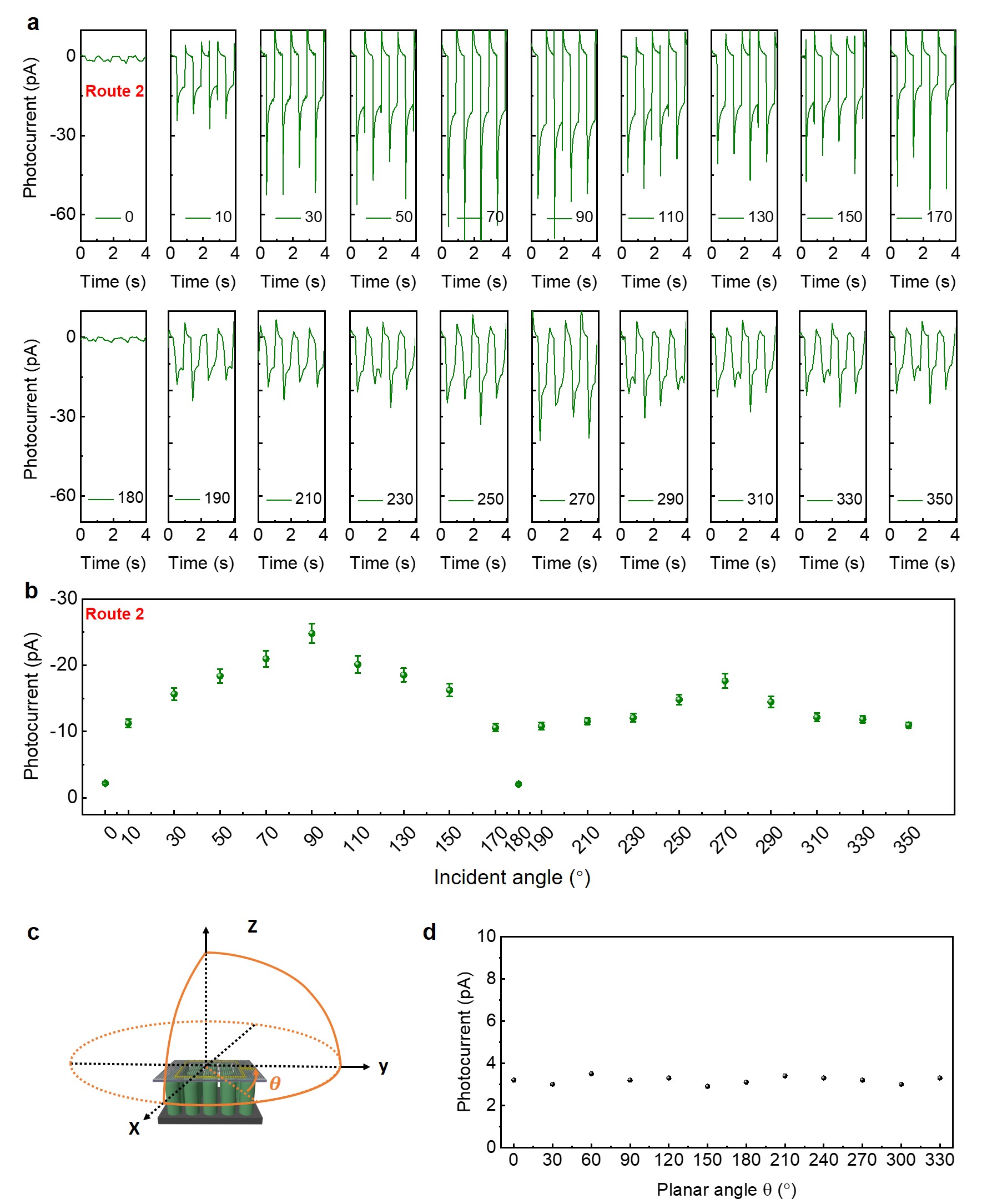


**Figure S9.** (a) The photoresponse and (b) the summarized photocurrent of the device as a function of the angle of incident light along with route 2. (c) Schematic diagram for blind spot analysis in quasi-omnidirectional detection (x-y plane). (d) The photocurrent along *θ*, in which the angle interval is 30°.


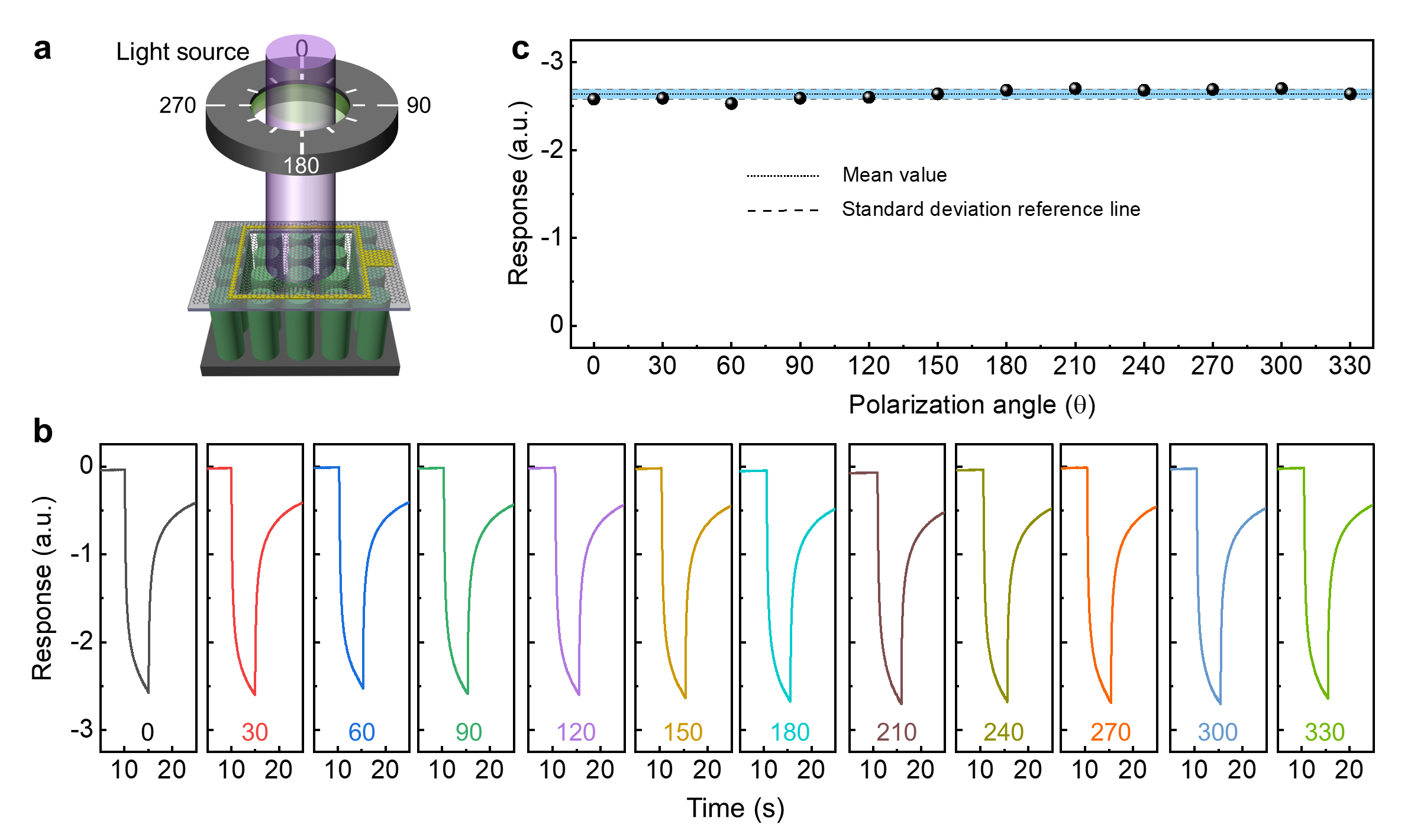


**Figure S10.** (a) Diagram of polarization information testing. (b) Light response intensity at different polarization angles. (c) Summary of light response intensity, with average and standard deviation reference lines showing minor fluctuations in light response intensity across different polarization angles.


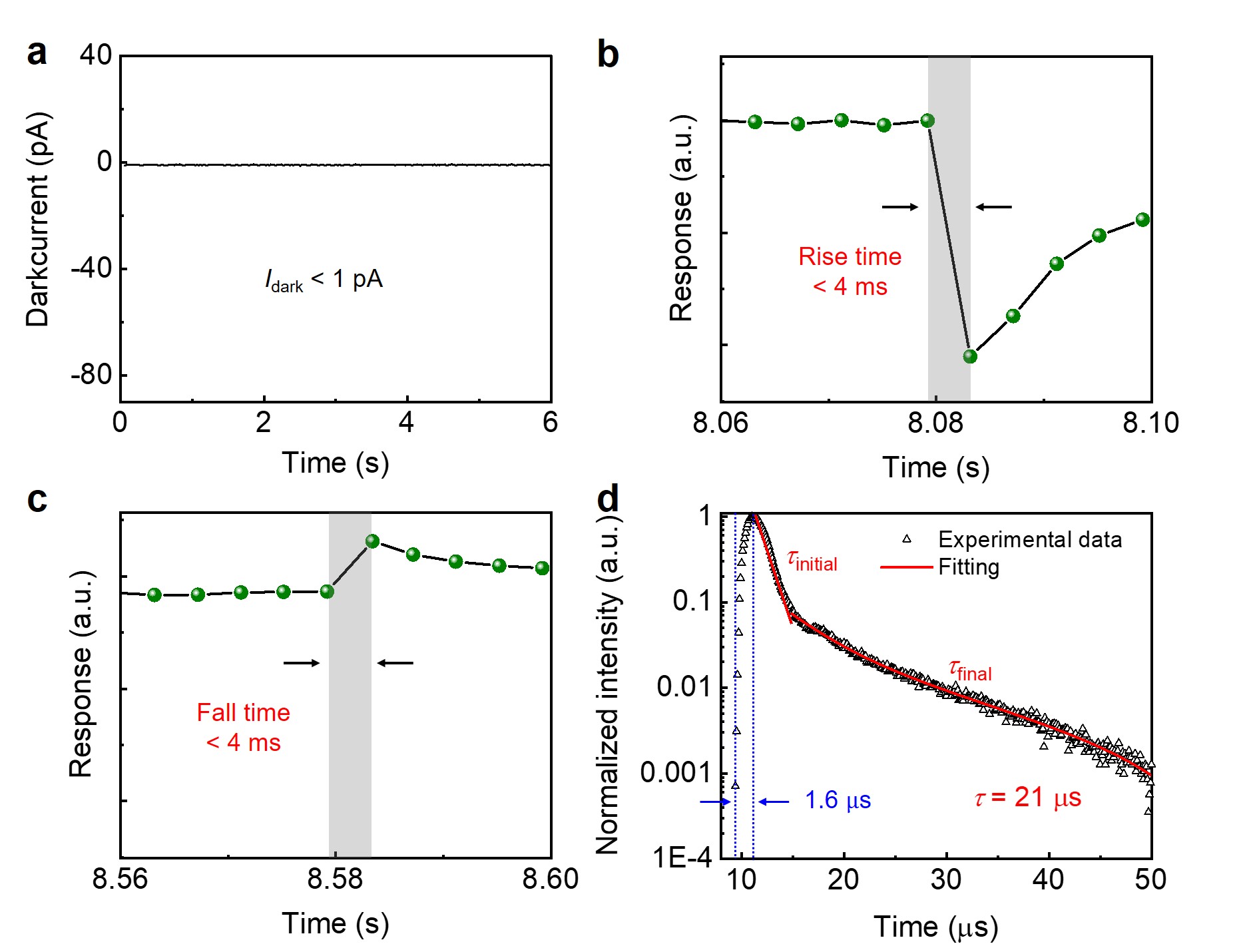


**Figure S11.** Performance under 0 V bias voltage. (a) Dark-current of the dual-mode device. (b) Rise time and (c) fall time enlarged from Figure 4f. The response speed of our device is significantly less than 4 ms. The minimum sampling interval of the test equipment is 4 ms. (d) Transient response time test, with the decay time (*τ*) fitted using a double exponential function.


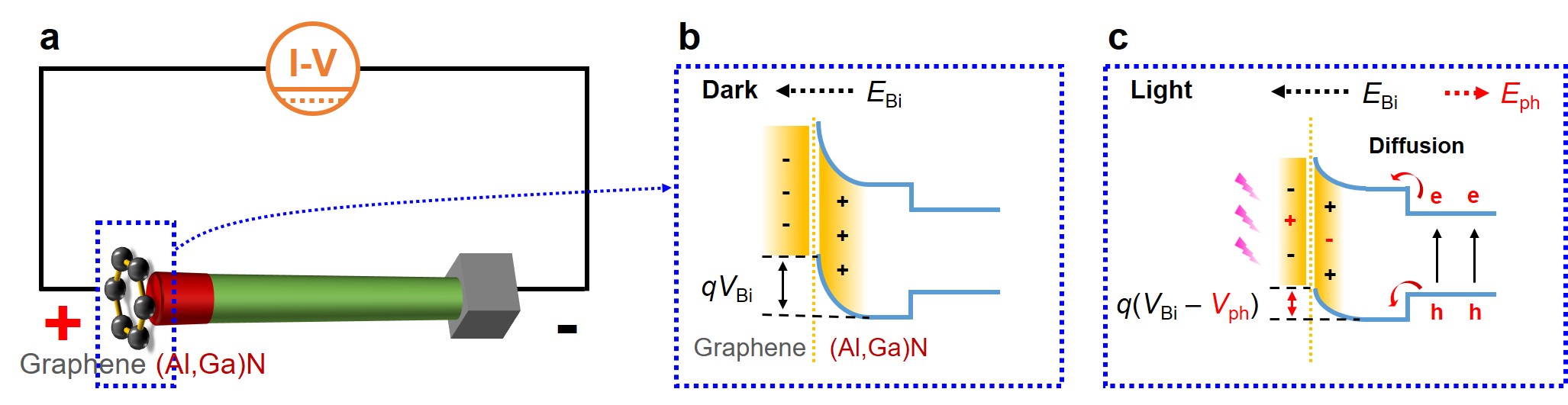


**Figure S12.** (a) The positive and negative terminals of the device. The barrier height in the (b) dark state and (c) light condition.


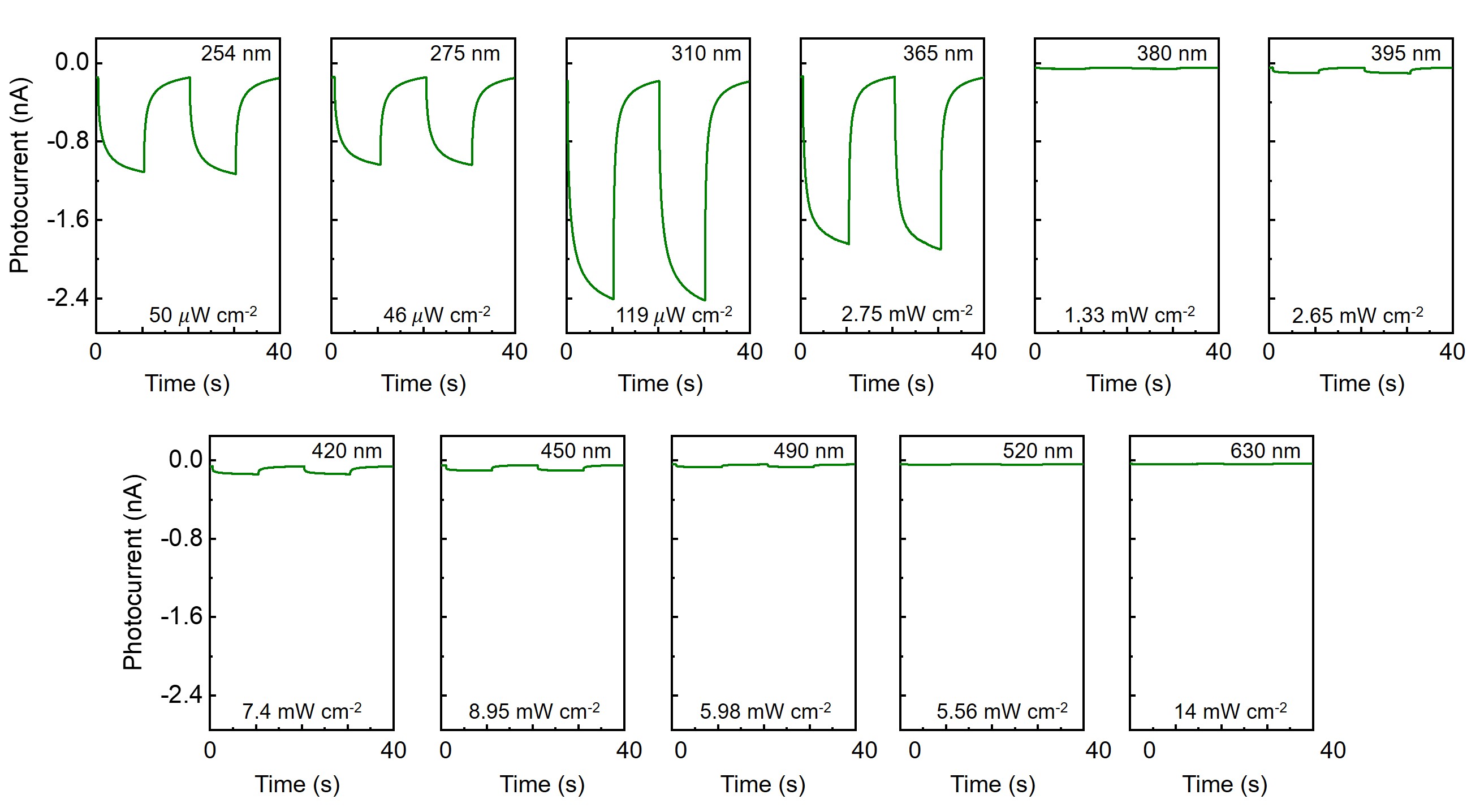


**Figure S13.** The photocurrent of the dual-mode device under -2 V bias voltage at different wavelength.


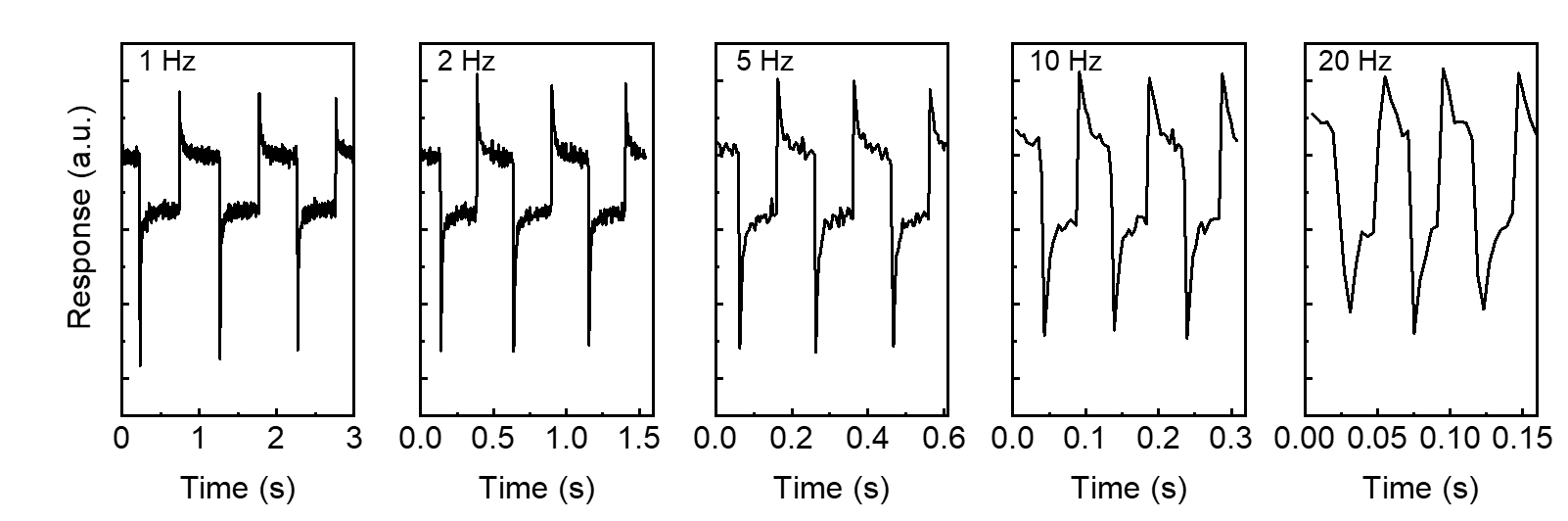


**Figure S14.** Photoresponse characteristics of the device at different frequencies at 0 V bias. The frequency ranges from 1 Hz to 20 Hz. During the testing process, the sampling interval of the equipment is 4 ms.


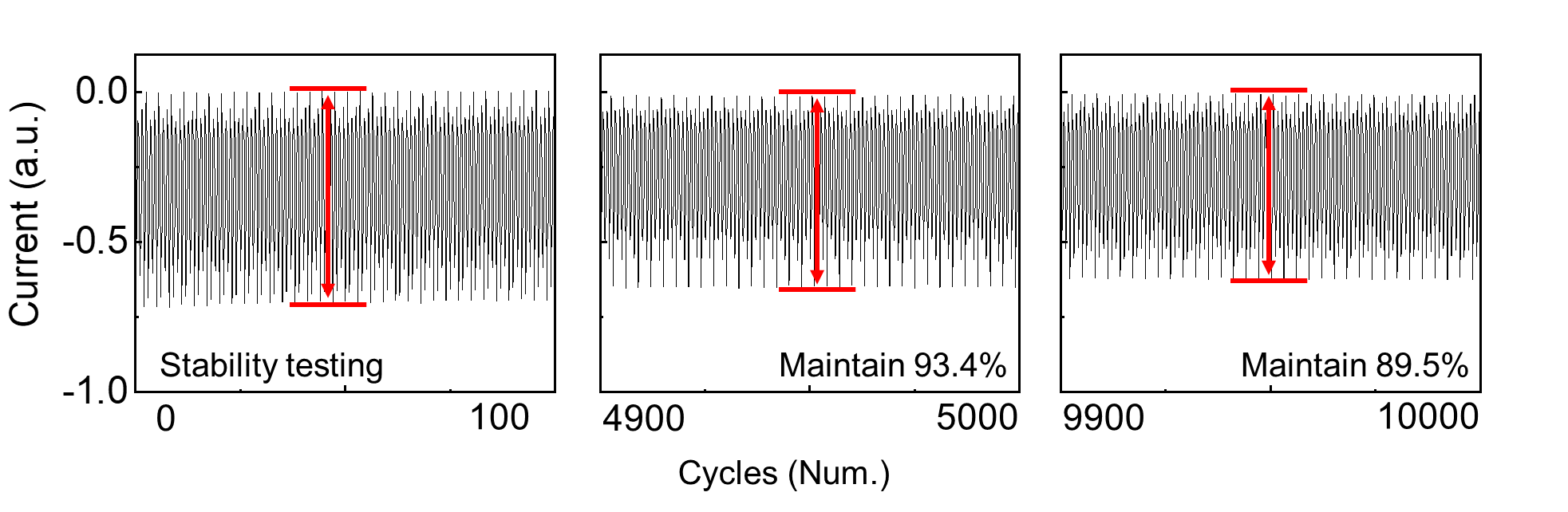


**Figure S15.** The device stability is demonstrated by 10,000 switching cycles at 5 Hz. The device maintains 89.5% of its initial photocurrent value after 10,000 cycles.


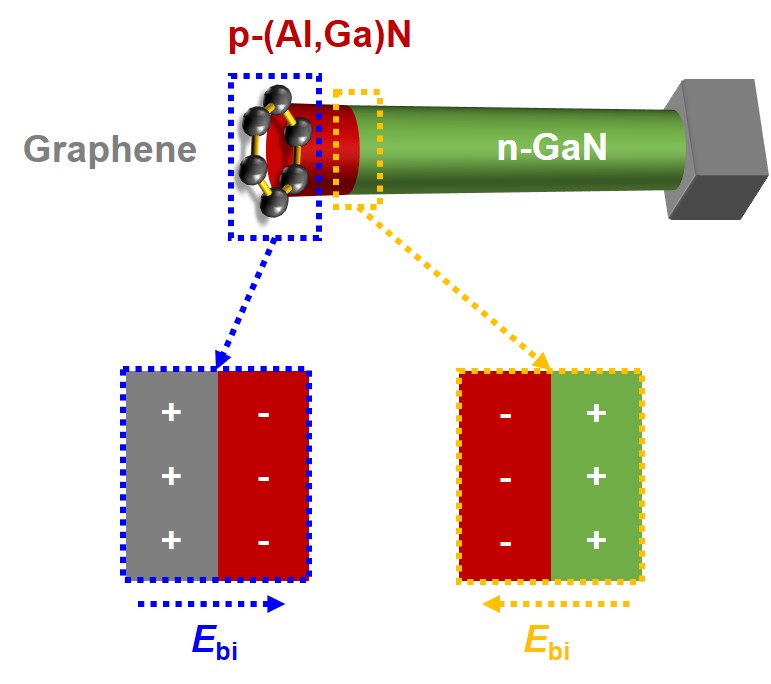


**Figure S16.** A design scheme for achieving both excitatory and inhibitory synaptic functions in dual-terminal devices. In this design, the graphene/p-(Al,Ga)N and p-(Al,Ga)N/GaN double heterojunctions generate built-in electric fields with opposite directions, respectively.


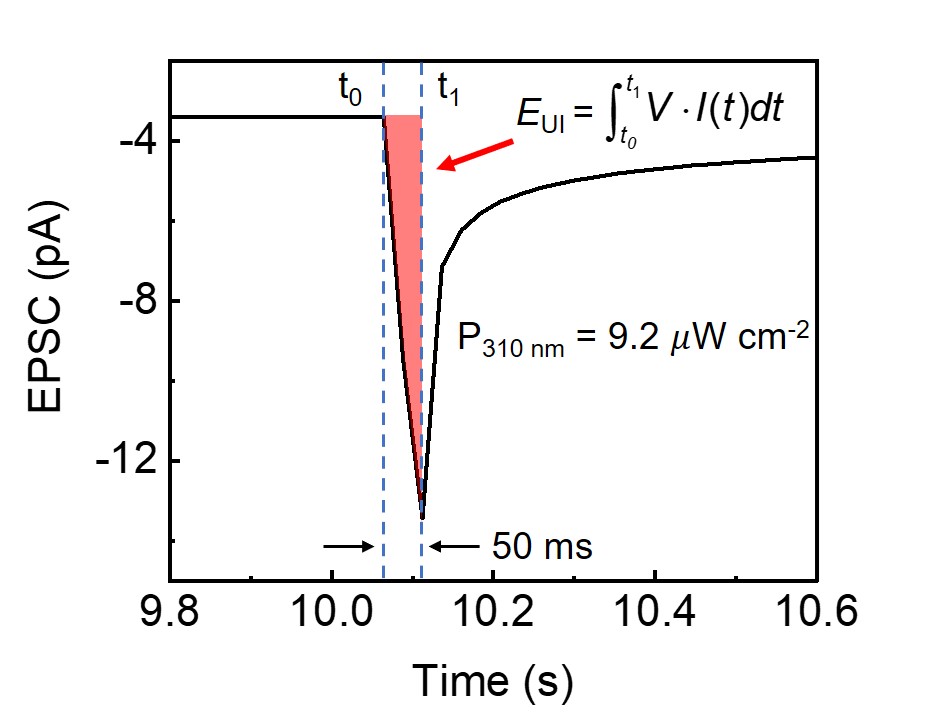


**Figure S17.** Enlarged image from Figure 5f (10 Hz). The area of the red region represents the power consumption of the device itself under a single optical stimulation. By using the approximate triangular area for calculation, the *E*_UI_ is 25 fJ.


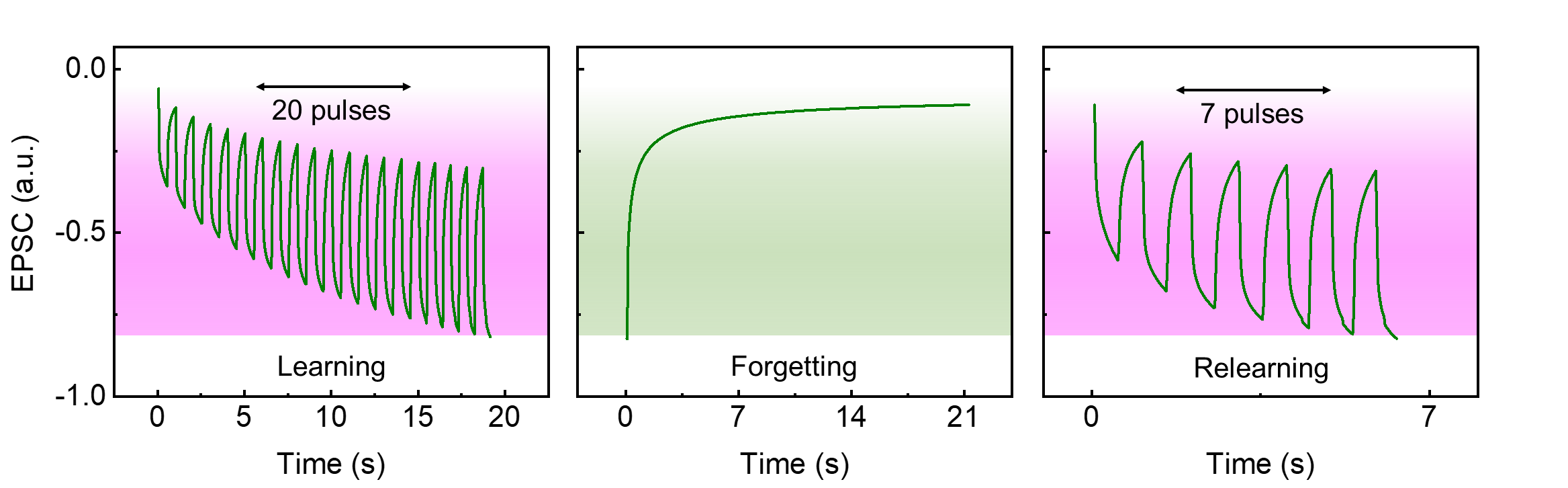


**Figure S18.** The device exhibits learning-experience behavior, including processes of learning, forgetting and subsequent relearning.


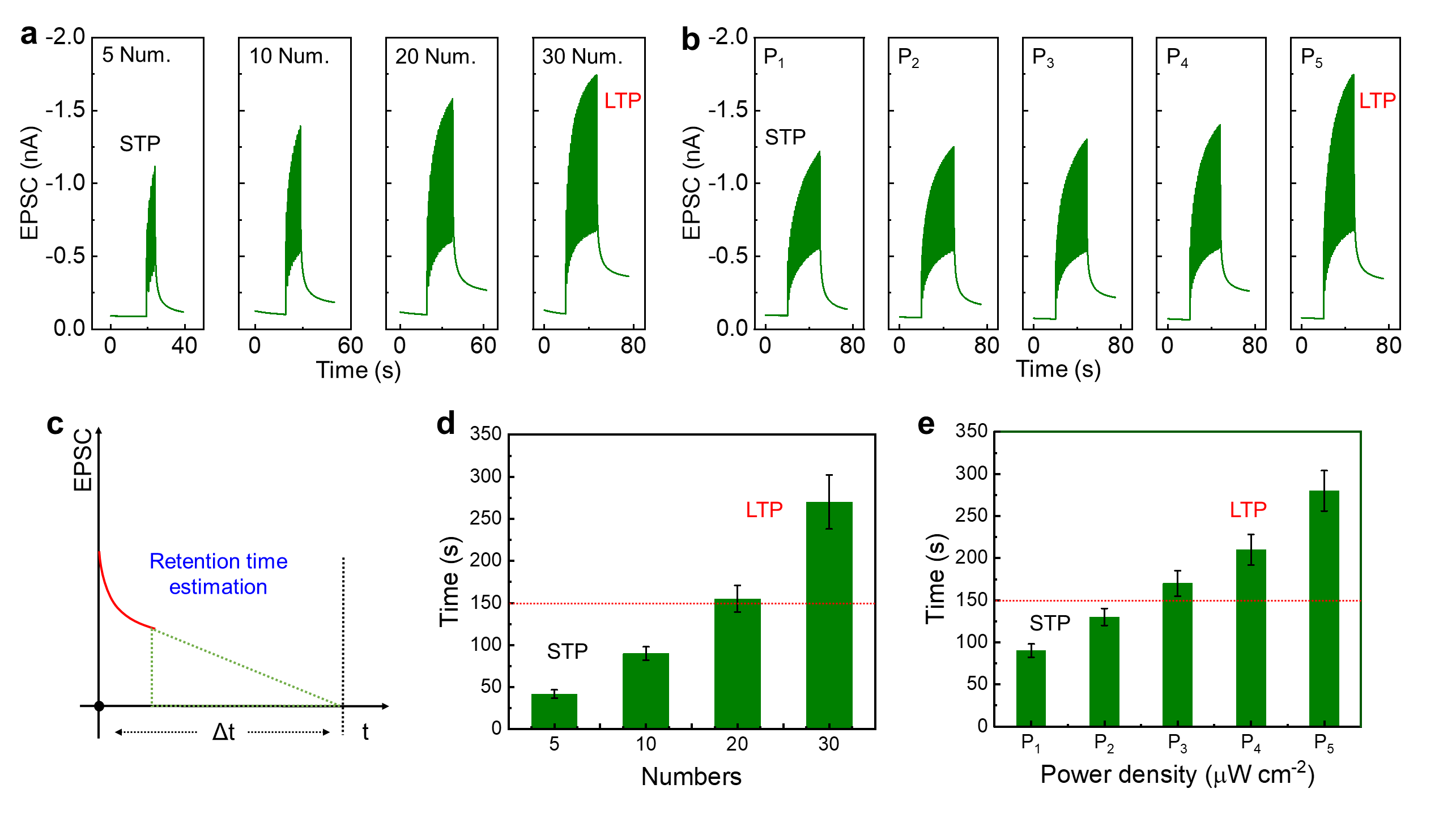


**Figure S19.** The EPSC of the device with different (a) light pulse numbers and (b) light intensities at -2 V bias voltage. The frequency of light pulse is 1 Hz. The light power density in (a) is 84.2 μW cm^-2^ at 310 nm. The number of light pulses in (b) is 30. The light power density in (b) range from 45.6 to 84.2 μW cm^-2^. STP/LTP classification based on retention time. (c) Schematic diagram of the estimation of retention time. Statistics of retention time (d) at different pulse numbers and (e) at different light intensities.

**
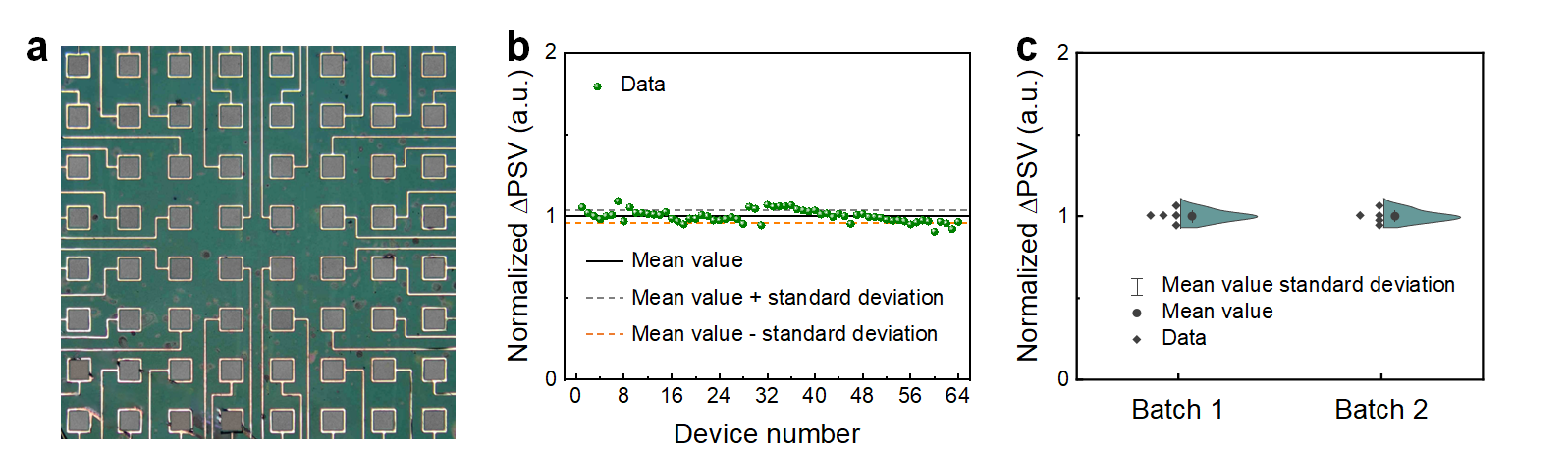
**

**Figure S20.** (a) Optical image of the 8 × 8 device array. (b) Uniformity of 64 devices. PSV fluctuation within the standard deviation range is within 4%. (c) The photoresponse intensity varies among devices from different batches. Five devices are randomly selected from each of two batches.


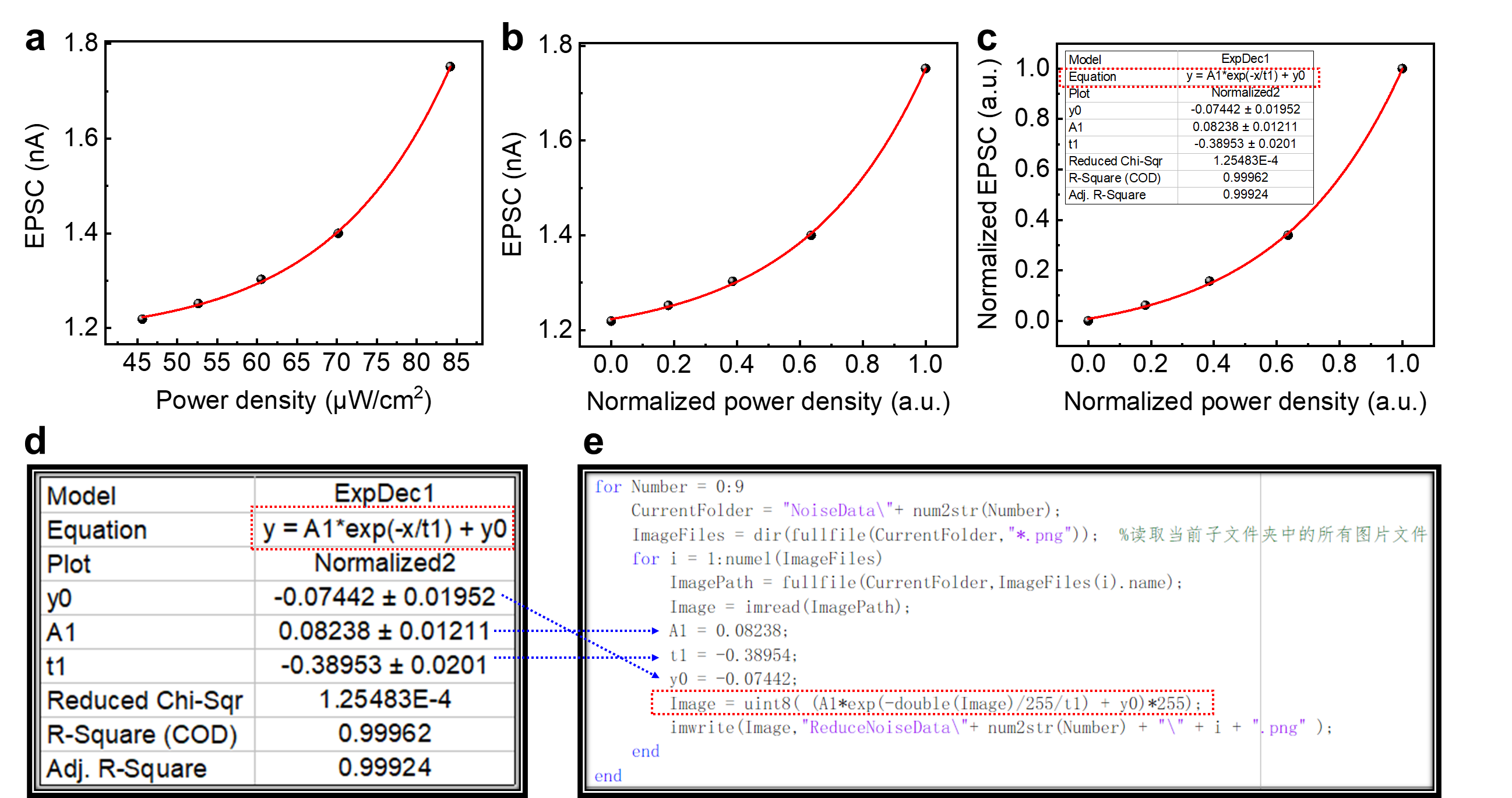


**Figure S21.** (a) A fitting curve of the relationship between current and light intensity, deriving from the EPSC in Figure S19b. (b) Fitting curve of current-normalized light intensity. (c) Fitting curve of normalized current-normalized light intensity. Fitting formula is y = A1*exp(-x/t1) + y0. (d) The specific parameters of the fitting formula. (e) The image denoising program is written based on a formula fitted from experiments. It is noteworthy that the basis for denoising is the fitted formula. In Figure S21c-e, the red boxes highlight the formulas used as the basis for the simulated denoising process, while the green arrows indicate the relevant parameters substituted into these formulas.


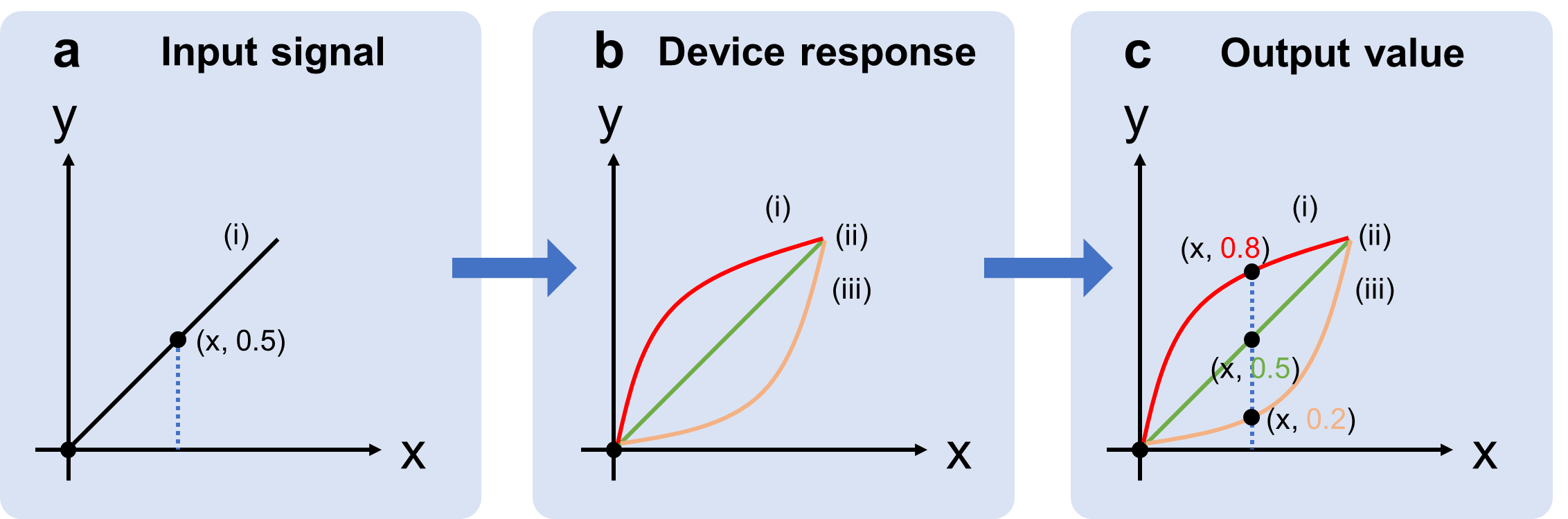


**Figure S22.** Diagram for three types of nonlinear responses. (a) Input signal, (x, 0.5) represent a noise point. (b) Device response characteristics, including (i), (ii) and (iii) types. (c) Output value of the noise point. The nonlinear responses of our device belong to type (iii).

As shown in Figure S23a, a classic artificial neural network (ANN) is composed of multiple layers of neurons, including convolutional layers, pooling layers and fully connected layers, which recognize images by extracting features layer by layer (64 input layers, 20 hidden layers and 10 output layers). The convolutional layers are responsible for extracting low-level features such as edges and textures. The pooling layers reduce the dimensions and computational complexity, and the fully connected layers combine the extracted features for final classification. In the traditional recognition processes, pixel values are scaled to a certain range (*e.g.*, 0 to 1) to facilitate neural network processing. Using the modified national institute of standards and technology (MNIST) dataset comprising 60,000 handwritten digit images, handwritten Arabic numerals ranging from 0 to 9 are selected to train an artificial neuromorphic visual system, with the training set and test set accounting for 70% and 30%, respectively.


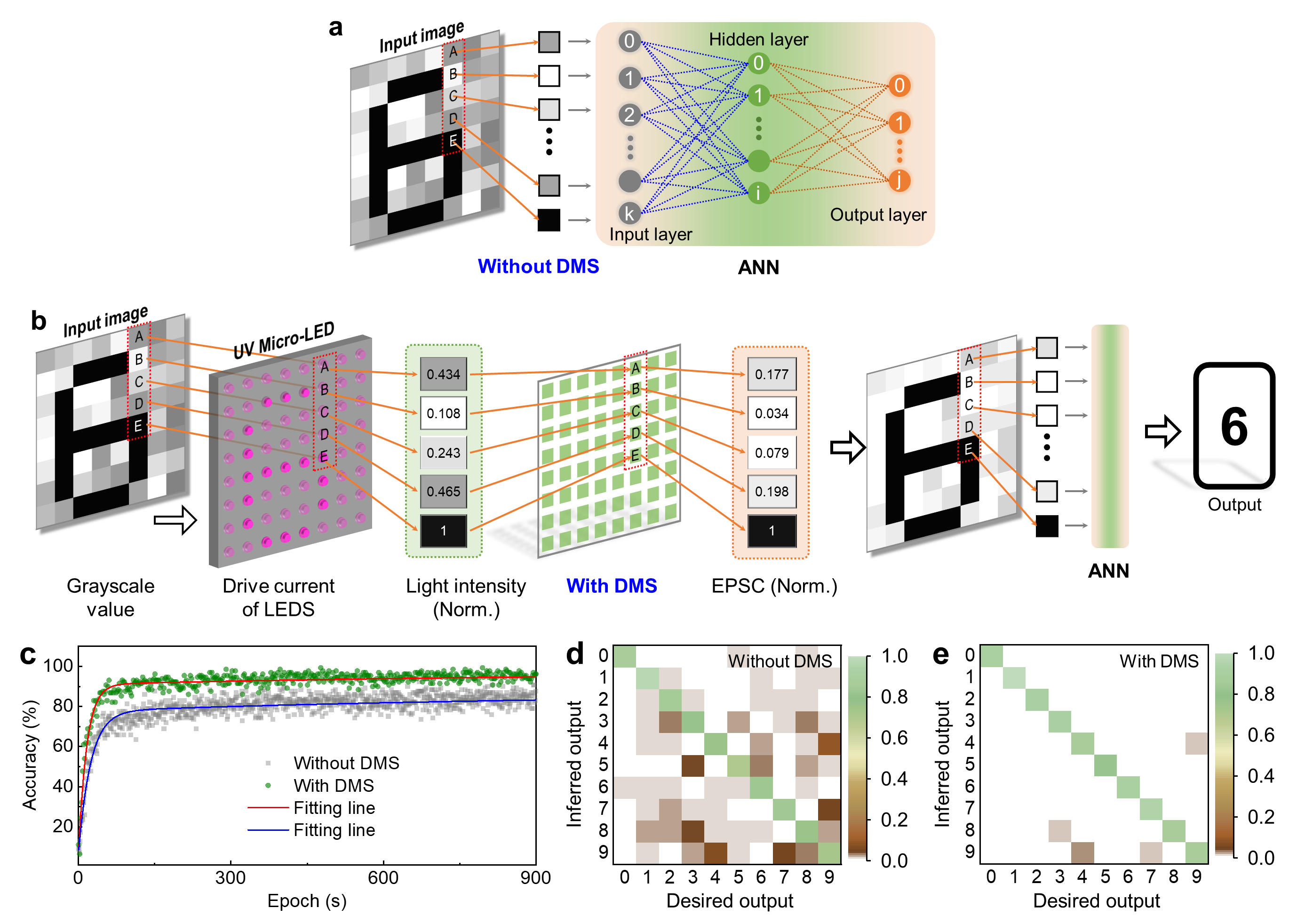


**Figure S23.** (a) The architecture of a classical artificial neural network (ANN) without the dual-mode synaptic devices (DMS). (b) Schematic diagram of the principle for using DMS to reduce noise in images. By converting grayscale values into driving currents for individual LEDs, a UV Micro-LED array can be utilized to encode inputs into multiple pulses. Here, we validate the feasibility of this approach using a single device and a single LED input light source, laying a theoretical foundation for future integration work based on ultra-high-density LED arrays and synaptic devices. (c) Comparison of recognition accuracy for training images before and after image preprocessing under 0.5 noise level. Confusion matrix training results for image recognition (d) without and (e) with preprocessing after 7500 epochs under 0.5 noise level.


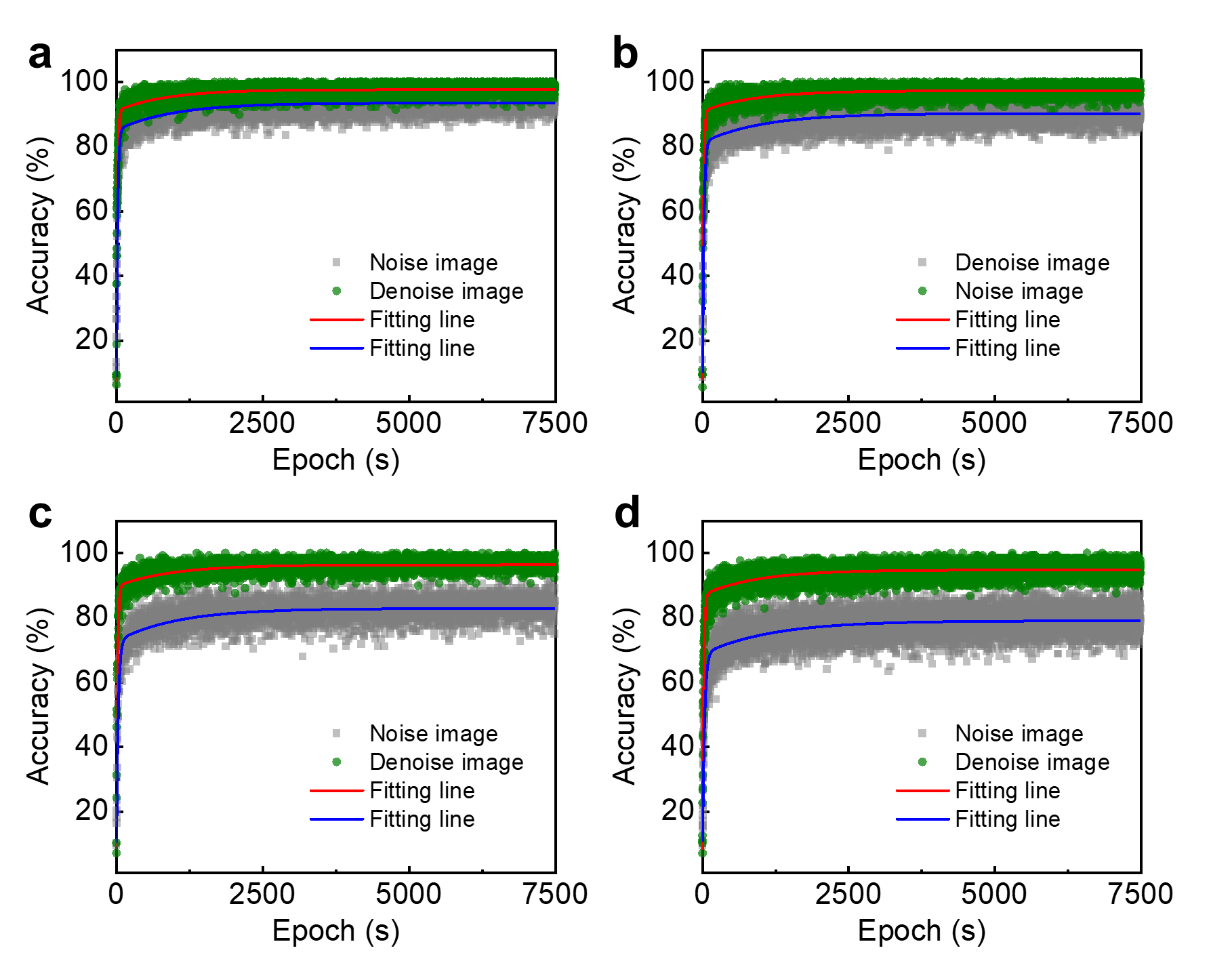


**Figure S24.** Evaluation of the recognition precision of the training images, both in their original states and after undergoing image preprocessing by the dual-mode transparent device, across varying levels of background noise: (a) 0.2, (b) 0.4, (c) 0.7 and (d) 0.9.


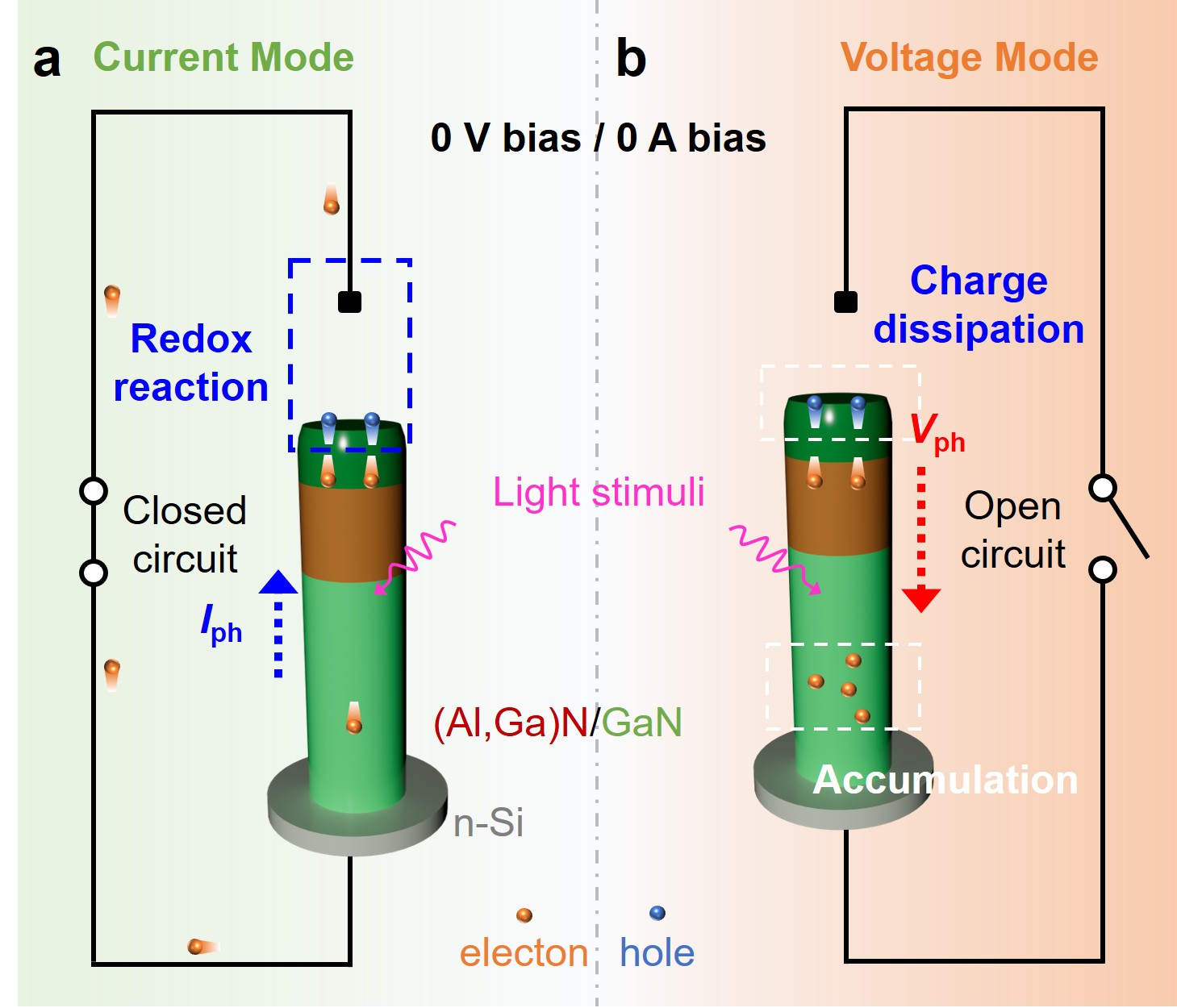


**Figure S25.** Schematic diagram illustrating the difference between (a) current mode and (b) voltage mode. The response in current mode is the photocurrent value (*I*_ph_), and that in voltage mode is the photovoltaic value (*V*_ph_).


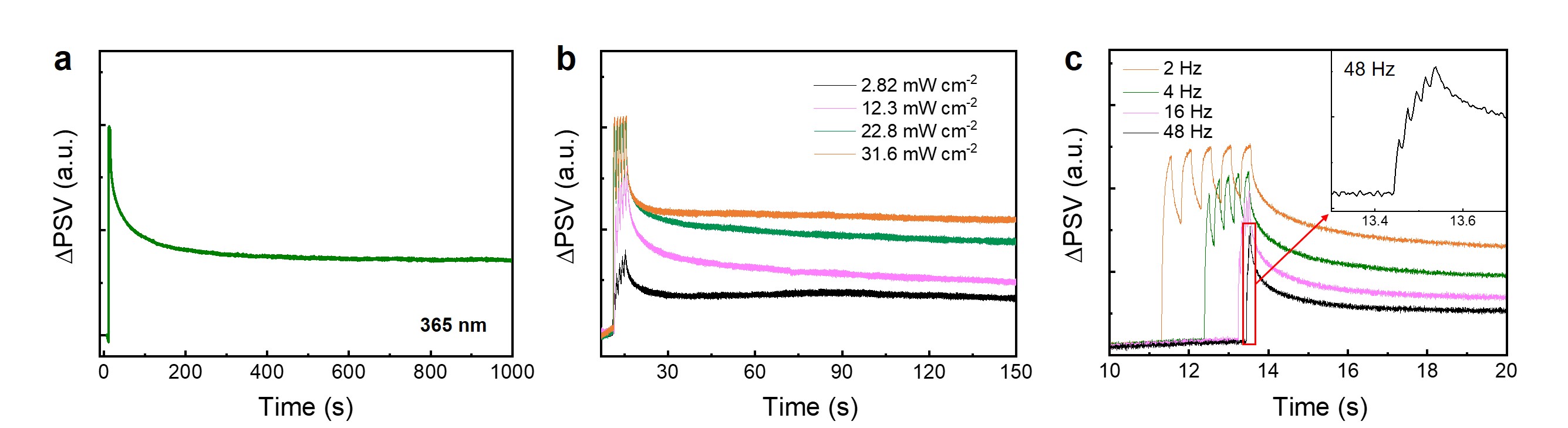


**Figure S26.** (a) Measurement of long-term memory retention. (b) Variation trend of PSV under different light intensities. (c) Response characteristics at different frequencies.


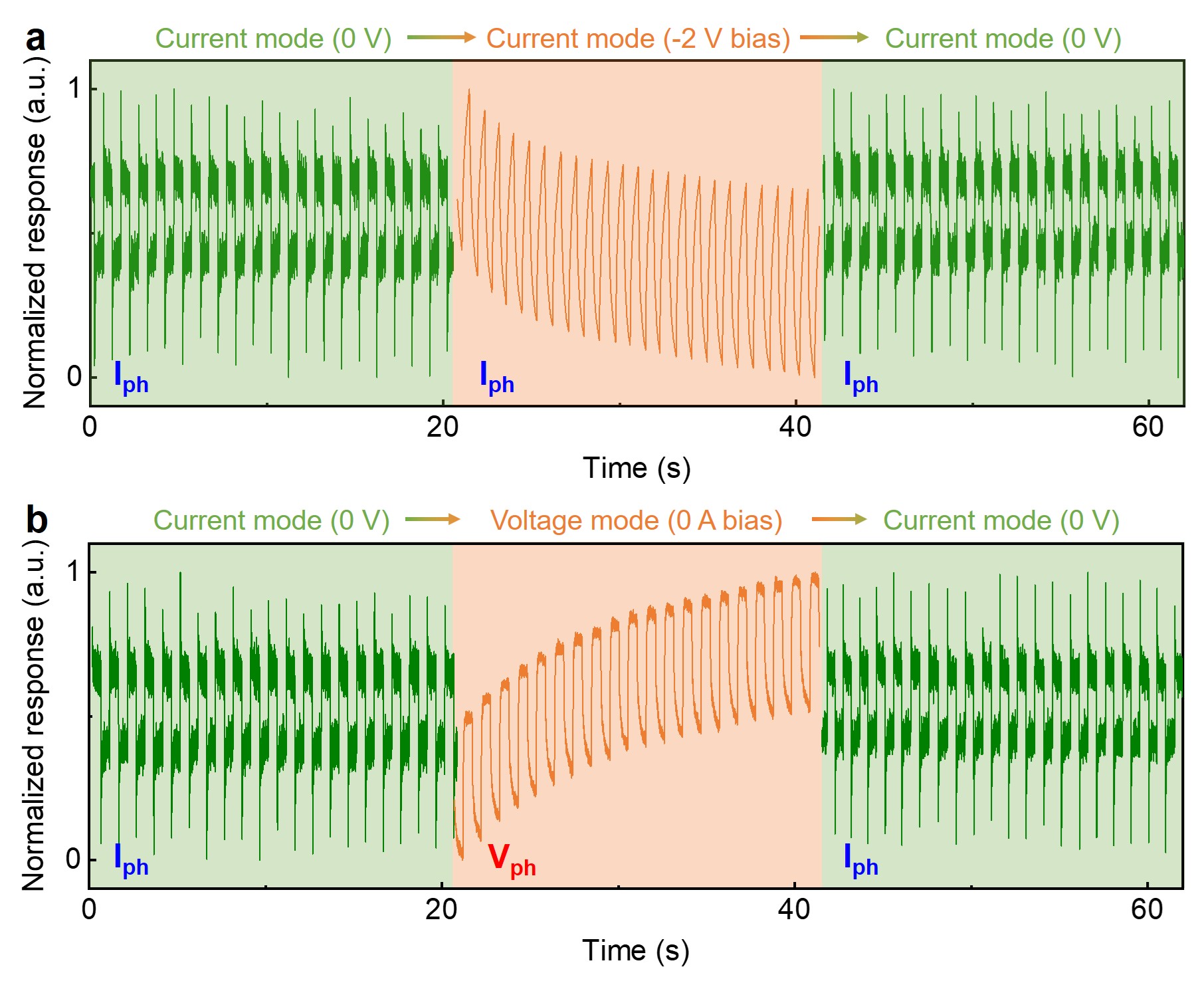


**Figure S27.** Evaluation of the impact of (a) switching from 0 V to -2 V and back to 0 V, as well as (b) switching from 0 V to 0 A and back to 0 V.


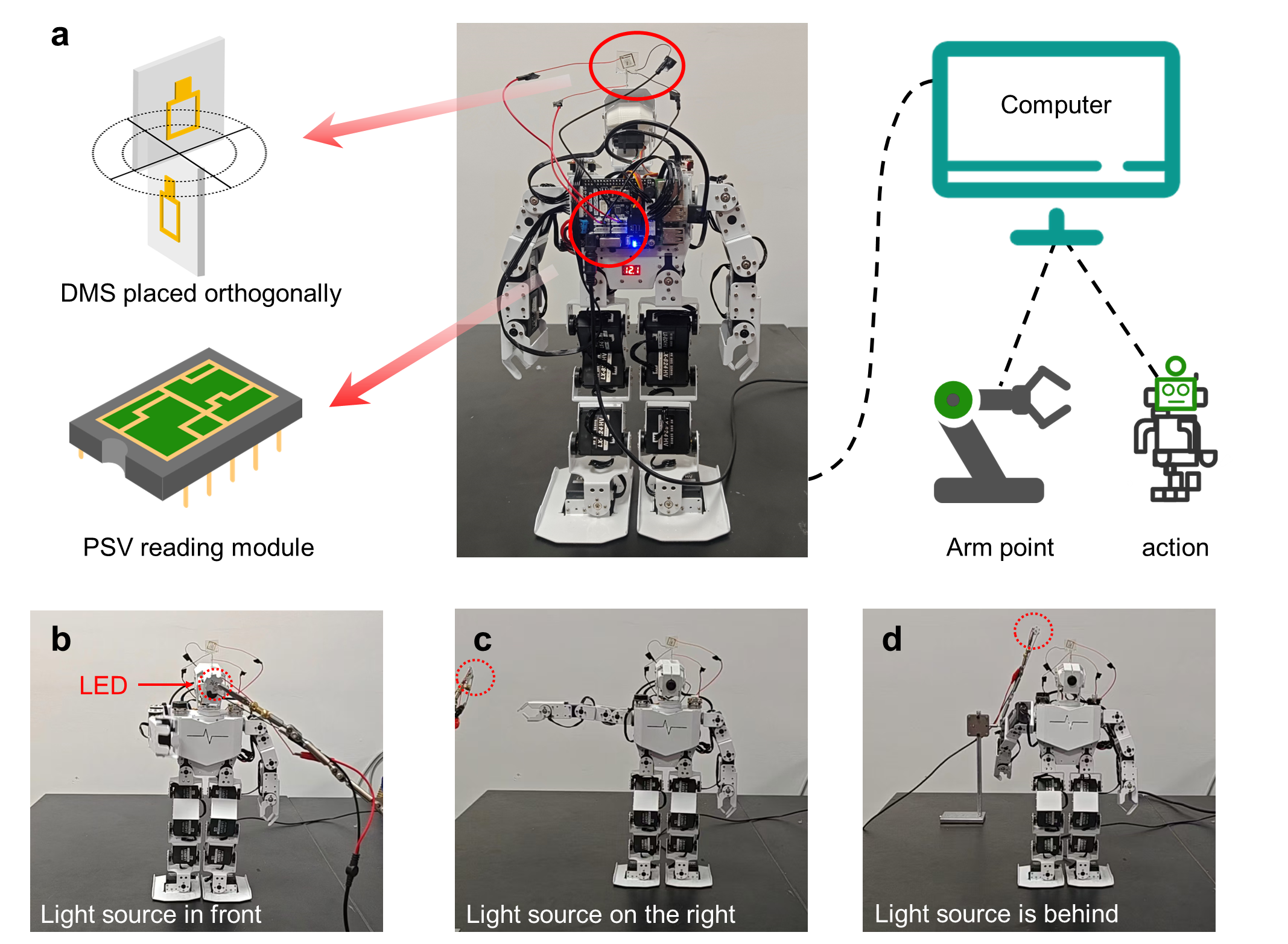


**Figure S28.** (a) Connection of dual-mode devices on the humanoid robot (in the head-top area). The relative positions of the LED light source (365 nm) on the humanoid robot are (b) directly in front, (c) on the right-hand side, and (d) directly behind.


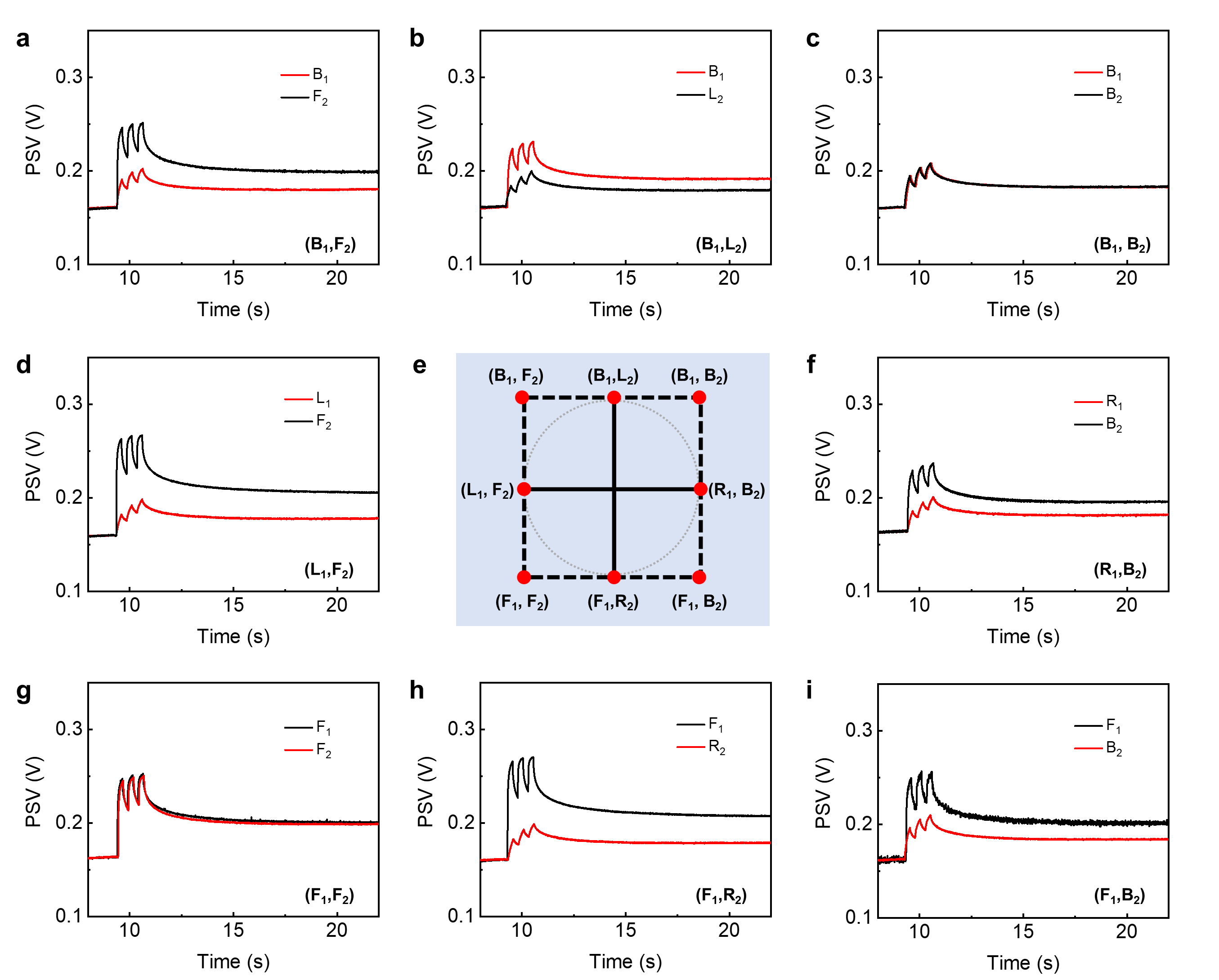


**Figure S29.** The PSV of two dual-mode devices placed orthogonally, under the eight coordinates (a) (B_1_, F_2_), (b) (B_1_, L_2_), (c) (B_1_, B_2_), (d) (L_1_, F_2_), (e) Eight coordinate positions on the same horizontal plane corresponding to these two devices, (f) (R_1_, B_2_), (g) (F_1_, F_2_), (h) (F_1_, R_2_), and (i) (F_1_, B_2_).


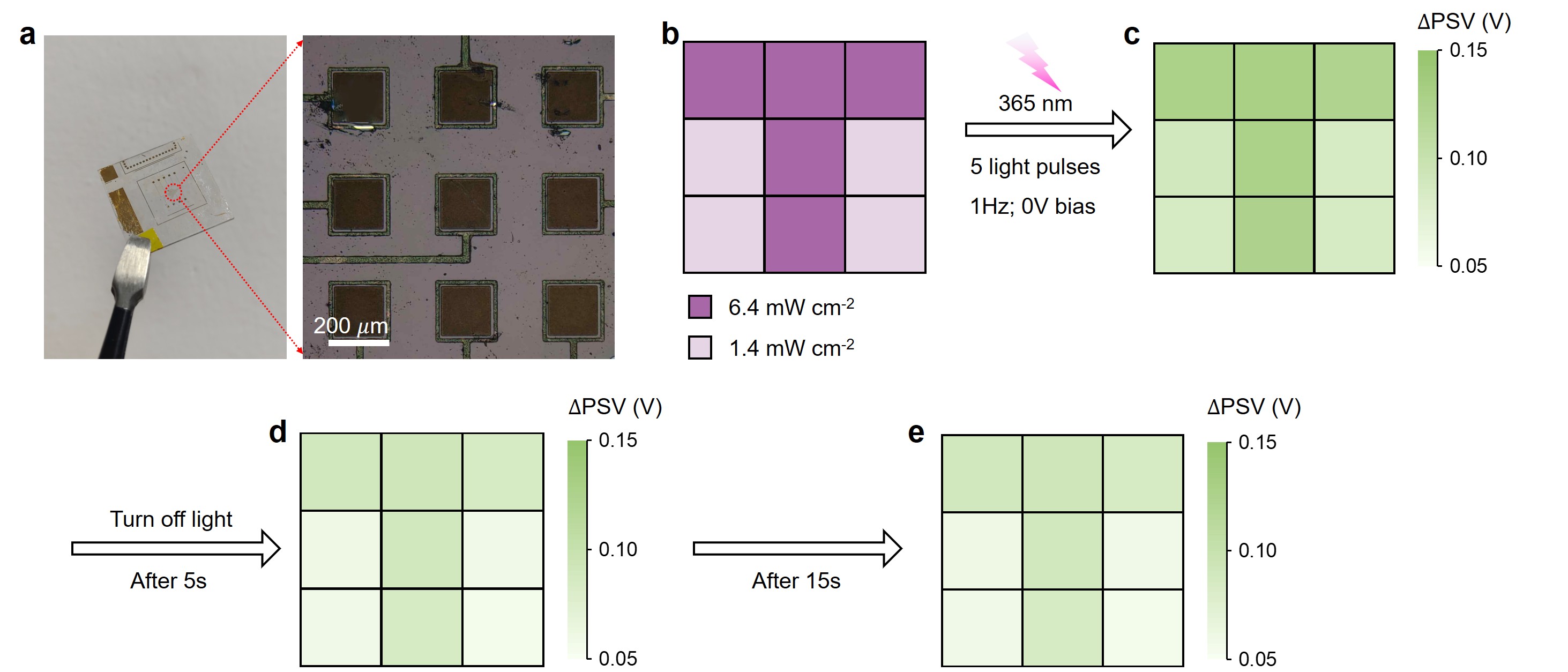


**Figure S30.** (a) Photograph of the 3 × 3 DMS device array. (b) The simulation of visual perception and memory using the DMS device. Light pulses of 365 nm with 6.4 mW cm^-2^ and 1.4 mW cm^-2^ are used. (c) The ∆PSV of the device array under 5 light pulses with 1Hz. After turning off the light, observe the ∆PSV value of the devices at (d) 5 seconds and (e) 15 seconds. All devices function under 0 V bias.

**Table S1.** Light power density of the incident light in Figure 4c, 4d, S8 and S9.

| Light | 365 nm | | | | | | | | | |
| --- | --- | --- | --- | --- | --- | --- | --- | --- | --- | --- |
| Degree (°) | 0 | 10 | 20 | 30 | 50 | 70 | 90 | 110 | 130 | 150 |
| *P*_inc_ (mW cm^-2^) | 7.1 | 12.4 | 12.4 | 12.4 | 12.4 | 12.4 | 12.4 | 12.4 | 12.4 | 12.4 |
| Degree (°) | 170 | 180 | 210 | 230 | 250 | 270 | 290 | 310 | 330 | 350 |
| *P*_inc_ (mW cm^-2^) | 12.4 | 7.1 | 28.3 | 28.3 | 28.3 | 28.3 | 28.3 | 28.3 | 28.3 | 28.3 |

**Table S2.** Comparison of the performance of this work with some recently reported self-powered photodetectors based on nano-materials.

| Materials | Dual-function integration | Detectivity /Sensitivity  (10^10^ Jones)  (Wavelength) | Angular  /Spatial resolution | Response  speed (ms) | UV/Visible  rejection ratio | Ref. |
| --- | --- | --- | --- | --- | --- | --- |
| Graphene/  (Al,Ga)N NWs | Yes | 0.32 (310)  0.0018 (365) | 20° /200 μm | N/A 0.0016/0.021 | 12908.7 | This work |
| Pt/GaN NWs | No | N/A (365) | N/A /5000 μm | N/A | 181.4 | [1] |
| Pt/(Al,Ga)N NWs | No | N/A (254) | N/A /5000 μm | 47/20 | 144.4 | [2] |
| (Al,Ga)N NWs | No | 4.71 (310) | 30° /1000 μm | 6200/6200 | 485 | [3] |
| (Al,Ga)N NWs | No | 2.51 (310) | N/A /1000μm | 2100/2200 | 977 | [4] |
| p-(Al,Ga)N/n-GaN | No | N/A (365) | N/A /1000μm | 1/1.3 | N/A | [5] |
| (In,Ga)N NWs | No | N/A (490) | N/A /5000 μm | 50/20 | N/A | [6] |
| (Al,Ga)N &  (In,Ga)N NWs | No | 1.86 (310)  0.001 (420) | N/A /1000 μm | 48/47  1600/2300 | N/A | [7] |
| Pt/p-(Al,Ga)N | No | N/A (255) | N/A /N/A | 200/100 | N/A | [8] |
| Cu/GaN | No | N/A (458) | N/A /10000 μm | 0.68/1.43 | N/A | [9] |
| n-(In,Ga)N/p-GaN NWs | No | 600 (275) | N/A /2190 μm | 0.014/0.007 | N/A | [10] |
| GaN/Cu_2_O NWs | No | 0.54 (365) | N/A /1612 μm | 42/65 | N/A | [11] |
| GaN/Ga_2_O_3_ | No | 11.5 (255) | N/A /7071 μm | 25/5 | N/A | [12] |
| GaN/CsPbBr_3_ NWs | No | N/A (310) | N/A /8000 μm | 740/7200 | N/A | [13] |
| p-(Al,Ga)N/n-GaN | No | N/A (255) | N/A /N/A | 50/100 | N/A | [14] |
| SnO_2_ Nanosheets | No | N/A (254) | N/A /10000 μm | 25/25 | 3395 | [15] |
| α-Ga_2_O_3_ Nanorods | No | N/A (260) | N/A /N/A | 76/56 | 33.7 | [16] |
| In_2_O_3_ Nanosheets | No | 44.3 (254) | N/A /10000 μm | 800/2200 | 1567 | [17] |

**Table S3.** Power consumption among this work and other recently reported GaN-based synaptic devices and other materials with ultra-low power consumption.

| Materials | *PPF* index  (%) | Applied bias  (|V|) | *E*_UIt_ (pJ) | *E*_SPt_ (pJ)  Consider device area | *E*_Pt_ (μJ)  Normalize to unit area | Ref. |
| --- | --- | --- | --- | --- | --- | --- |
| Graphene/(Al,Ga)N/  GaN NWs | 163e | 0.1 | 0.025 | 1820 | 0.455 | This work |
| GaN/Ga_2_O_3_ | 163 | 0.001 | 0.05 | N/A | 11.8 | [18] |
| GaN/Ga_2_O_3_/GaN | 144 | 5 | 584 | 623.5 | 0.279 | [19] |
| (Al,Ga)N NWs | 210 | 3 | 2600000 | 6.4 | 0.064 | [20] |
| (Al,Ga)N NWs | 166 | 5 | 0.558 | 0.046 | 115 | [21] |
| GaN NWs | 150 | 5 | 2.72 | 0.122 | 61 | [22] |
| GaN MWs | 108 | 3 | 500 | N/A | N/A | [23] |
| Si doped GaN film | 160 | 0.01 | 33.4 | N/A | 1.14 | [24] |
| (Al,Ga)N/GaN film | 110 | 0.001 | 1850 | 0.128 | 0.04 | [25] |
| GaN NWs | 130 | 4.5 | 300000 | N/A | N/A | [26] |
| NaAc/PVA | 190 | 0.001 | 0.000132 | N/A | N/A | [27] |
| P3HT-b-PPI | 185 | 0.001 | 0.00144 | N/A | 1700 | [28] |
| P(VDF-TrFE) | 117 | 5 | 0.00084 | N/A | N/A | [29] |

As shown in Figure S19b, P_1_-P_5_ represent five gradually increasing optical power densities (Table S4), with the smallest P_1_ (45.6 μW cm^-2^) normalized to 0 and the largest P_5_ (84.2 μW cm^-2^) normalized to 1. The EPSC is the maximum value extracted from Figure S19b (30th pulse). For an input digital image (such as the "6" in Figure 5i), the light intensity corresponding to the minimum grayscale value is P_1_, normalized to 0, and the light intensity corresponding to the maximum grayscale value is P_5_, normalized to 1. In other words, grayscale values between 0 and 1 correspond linearly to optical power densities ranging from 45.6 to 84.2 μW cm^-2^. Obviously, according to this mapping, the optical power densities represented by A, B, C, D and E are 62.4 μW cm^-2^, 49.8 μW cm^-2^, 55 μW cm^-2^, 63.6 μW cm^-2^ and 84.2 μW cm^-2^, respectively.

**Table S4.** Light power density and the corresponding EPSC values, as well as their normalized fitting relationship

| P_Num_. | Power density  (μW cm^-2^) | EPSC (nA) | Norm. P_1-5_ (a.u.) | Norm. EPSC (a.u.) |
| --- | --- | --- | --- | --- |
| P_1_ | 45.6 | 1.219 | 0 | 0 |
| P_2_ | 52.6 | 1.252 | 0.182 | 0.06191 |
| P_3_ | 60.5 | 1.303 | 0.386 | 0.1576 |
| P_4_ | 70.2 | 1.4 | 0.636 | 0.33959 |
| P_5_ | 84.2 | 1.752 | 1 | 1 |

**Table S5.** The mapping relationship between the power density of five representative pixels and EPSC

| Related  pixel points | *P*_inc_  (μW cm^-2^) | EPSC (nA) | Norm. *P*_inc_ (a.u.) | Norm. EPSC (a.u.) |
| --- | --- | --- | --- | --- |
| A | 62.4 | 1.313 | 0.434 | 0.177 |
| B | 49.8 | 1.237 | 0.108 | 0.034 |
| C | 55 | 1.261 | 0.243 | 0.079 |
| D | 63.6 | 1.324 | 0.465 | 0.198 |
| E | 84.2 | 1.752 | 1 | 1 |

**Table S6.** The power consumption of this dual-mode device versus other devices during the preprocessing process.

| Number of pulses | *V* | *I* | *t* | Power consumption | Ref. |
| --- | --- | --- | --- | --- | --- |
| 30 | -2 V | ~0.726 nA | 0.5s | 2.18 × 10^-8^ J | **This work** |
| 100 | 5 V | ~40 nA | 0.5s | 1 × 10^-5^ J | [19] |
| 100 | 0.1 V | ~12 nA | 0.2s | 2.4 × 10^-8^ J | [30] |

As illustrated in Figure S19b, we utilize the EPSC value obtained at the 30th pulse as the initial data for fitting the nonlinear response, during which a voltage of -2 V is applied to the device. Subjected to 30 pulses, subsequent calculations reveal that the energy consumption of a single device is approximately 2.18 × 10^-8^ J, which is determined using the function . In this equation, *V_i_* represents -2 V, *I_i_* signifies the peak value of the 30 pulses observed under P_5_ (averaging 0.726 nA per pulse), and t is 0.5 s.

**Supplementary Note 1. Applying a bias voltage can effectively promote the separation of carriers.**

In our dual-mode device, the applied bias voltage introduces an additional electric field to the device. Changes in this electric field affect the movement and distribution of carriers, thereby altering their recombination behavior. Under bias, the external electric field facilitates the separation of electrons and holes, pulling them apart and propelling them along the direction of the electric field, which reduces their chances of collision and recombination, significantly decreasing carrier recombination (Figure S4b and S4c). Furthermore, the application of the bias voltage enhances carrier mobility, enabling carriers to traverse the graphene/(Al,Ga)N heterojunction and the (Al,Ga)N/GaN interface more rapidly, further lowering the carrier recombination rate. In contrast, at a bias voltage of 0 V, photogenerated carriers primarily rely on diffusion driven by concentration gradients (Figure S4a). Due to the ineffective separation of electron-hole pairs, recombination is prominent, especially within the GaN region of the device. The trend of current variation under bias voltage provides good evidence for our conclusion. In brief, the bias voltage helps promote the separation of photogenerated carriers within the nanowires, thereby enhancing the photoelectric performance and efficiency of the device.

**Supplementary Note 2.** **Exploring the benefits and collaborative applications of dual-mode device integration.**

**A) The benefit of achieving dual-mode functionality**

The 360° omnidirectional photodetector can achieve full coverage without the need to adjust the detection direction or rely on multiple devices working together, thereby eliminating potential monitoring blind spots associated with traditional photodetectors. In large open spaces or complex environments, it can accomplish comprehensive monitoring in a single pass, enhancing efficiency. Artificial synaptic functionalities mimic the working mechanisms of biological neurons, enabling the system to perform more flexible and efficient signal processing. This functionality holds immense potential especially in areas such as brain-like computing and neural network acceleration. By embedding artificial synapses in photodetectors, immediate processing of optical signals can be effectively achieved.

By integrating self-driven 360° photodetectors with artificial synaptic functionalities within the same device, the complexity and size of the equipment can be effectively reduced. Traditional systems typically require separate implementations for photodetection and information processing functions, whereas the device in this study, by fusing these two functions, can significantly enhance system integration and operational efficiency. Additionally, combining photodetection with intelligent processing enables efficient capture, processing and response to optical signals, providing compact and efficient solutions for future intelligent systems, such as brain-like computing and sensor networks. In brief, the integration of multiple functions within a single device is conducive to enhancing the intelligence level of the device and plays a role in photoelectric sensing and intelligent decision-making in fields such as robotics and autonomous driving.

**B) Combination application scenarios**

Dual-mode devices combining photodetection and artificial synaptic functionalities can play a crucial role in many emerging applications. For instance, in ground-power-grid early warning systems, omnidirectional dual-mode devices can monitor the impact of solar radiation or fire radiation on power grids and communications. By leveraging the threshold response characteristics of synaptic devices, they can swiftly assess environmental changes and issue early warnings. In medical facilities, public places, and other environments, omnidirectional UV detectors monitor the radiation range and intensity of UV sterilization equipment in real-time. Synaptic devices dynamically adjust the radiation direction and duration to ensure both sterilization efficiency and safety. In Figure 5i-l, we demonstrate the application of synaptic functionality in a dual-mode device, utilizing its nonlinear response characteristics to preprocess image noise reduction. In fact, when the device operates at a 0V bias voltage, it exhibits self-driven characteristics, eliminating the need for the external power supply in this state and effectively reducing energy consumption. As shown in Figure 6, upon the humanoid robot sensing UV light, we leverage the photodetector functionality of device for direction recognition. On the other hand, it can be determined whether to switch to the synaptic device based on the signals collected by the device array. By combining both characteristics, we can achieve rapid signal processing and intelligent decision-making.

**C) Technical challenges and innovation**

This combination is quite different from traditional single-function devices. Its innovation lies in performance enhancement and application expansion through multimodal integration. The development of GaN-based dual-mode devices not only requires a deep and comprehensive understanding of the underlying physical mechanisms, but also necessitates the proposal of novel approaches in device fabrication. Therefore, the combination of self-driven 360° photodetectors and artificial synaptic functionalities in dual-mode devices not only exhibits unique innovation but also provides a novel approach for the integration and performance enhancement of intelligent systems.

**Supplementary Note 3. Discussions on the introduction of V_O_ and the adjustment of V_O_ concentration.**

During the molecular beam epitaxy (MBE) process, we ensured a high-vacuum environment in the growth chamber without oxygen. Hence, it is difficult to introduce V_O_ in the MBE process. In fact, although GaN nanowires exhibit a low oxidation rate in a conventional air environment, a noticeable oxide section will gradually be formed over a period of hours to days [31]. Furthermore, as the oxide section thickens, the resistance to oxygen diffusion through the section increases, leading to a gradual decrease in the oxidation rate and a tendency for the thickness to saturate. In this work, on the one hand, the (Al,Ga)N nanowires for device fabrication were stored in a natural environment for an extended period. On the other hand, during the lift-off process of the (Al,Ga)N nanowire samples, nitric acid was used to etch the underlying AIN sacrificial layer. As a strong oxidant, nitric acid can undergo chemical reactions with the oxide section on the surface of (Al,Ga)N leading to the dissolution of the surface oxide section and increasing the probability of V_O_ formation. In brief, the introduction of V_O_ primarily relies on the natural oxidation characteristics of GaN-based materials and the synergistic effect of using nitric acid during the lift-off process.

In addition, the concentration of V_O_ can be controlled. On the one hand, moderate etching of the surface in nitric acid may remove some surface oxygen atoms, thereby introducing V_O_. Our previous work compared gallium nitride nanowire samples with and without nitric acid treatment [20]. In that work, the samples treated with nitric acid exhibited significant changes in both photoconductivity effect and relaxation time, indicating that the concentration of V_O_ can be regulated through treatment with acidic solutions.

On the other hand, annealing under high temperature and oxygen atmosphere may be another effective method for regulating the concentration of oxygen vacancies. Research has shown that gallium oxide frequently includes V_O_, having a notable impact on its electronic and physical characteristics [32, 33]. To demonstrate its feasibility, we adopted a relatively extreme method for verification, where GaN samples were annealed in an oxygen environment at a high temperature of 1000°C for 10 minutes. The annealing results showed that the nitrogen (N) content in the GaN samples was much lower than the oxygen (O) content, indicating that GaN was almost entirely converted to GaOx under extreme high-temperature treatment (Figure S5). Therefore, if we can precisely control the annealing conditions such as temperature, time, and oxygen concentration, the concentration of V_O_ on the surface of GaN nanowires can also be effectively regulated. In future work, it is necessary to conduct more in-depth and systematic research on the regulation of V_O_ concentration and its impact.

**Supplementary Note 4. Discussion on the variation of transmittance within the visible light spectrum.**

**A) Influence of substrate transmittance**

The transmittance of ITO itself in the visible light range is not completely uniform, and its transmittance is affected by its thickness, preparation process, and doping level. Especially in the short-wave (blue light) or near-infrared regions, the transmittance may decrease, thereby affecting the overall measurement results. As shown in Figure S6a, the ITO substrate exhibits significant variations in the visible light range. During the transmittance measurement process, when the wavelength reaches the absorption peak of the substrate, the overall transmittance of the device decreases, and when the substrate absorption weakens, the transmittance increases again.

**B) Interfacial reflection and interference effects**

Due to the refractive index difference between the (AI,Ga)N material and the ITO substrate, light reflects and transmits between them. This multilayer structure may form a thin-film interference effect, where different wavelengths of light photons reflect and interfere differently at the (Al,Ga)N/ITO interface, resulting in enhanced or reduced transmittance at specific wavelengths. For example, the increase in transmittance within the 550-600 nm range may be related to the enhancement of interference, while the decrease within the 500-540 nm and 600-660 nm ranges may be related to the destruction of interference. To further verify this, we transferred thinner (Al,Ga)N nanowires films onto ITO substrates and found that the transmittance also decreased within certain wavelength bands. Since a thinner comparative (Al,Ga)N nanowires film (~500 nm) was used, its transmittance changes were closer to the fluctuation trend of the substrate (Figure S6b and S6c).

In summary, the combined effects of the absorption of the ITO substrate itself and the reflection and interference effects at the (Al,Ga)N/ITO interface lead to transmittance attenuation in certain wavelength bands. Considering that our dual-mode device operates in the UV range, changes in transmittance in the visible light range do not affect the main conclusions of this work.

**Supplementary Note 5.** **Discussion on** **the impact of bias voltage on the number of holes involved in V_O_ ionization.**

Compared with those under a certain external voltage (*e.g.* 2 V and -2 V), the photocurrent generated by the device is much smaller under a bias voltage of 0 V (Figure 3a and 3b), and does not exhibit synaptic response characteristics regardless of the illumination conditions at 310 nm or 365 nm. When a bias voltage of -2 V or 2 V is applied, due to the effective separation of electrons and holes by external electric fields, the photocurrent value increases significantly compared to that at 0 V bias, and exhibits significant synaptic response characteristics, indicating that a large number of holes participate in the ionization of V_O_ during carrier transport. In summary, by comparing the photocurrent levels under different voltages and the presence of synaptic response characteristics, we propose that the number of holes involved in the ionization of V_O_ at 0 V is smaller than those at a certain external voltage.

Furthermore, during double I-V characterization, the hysteresis loop, which is delineated by the forward and reverse scanning paths, conveys essential details regarding the device non-equilibrium charge dynamics [34, 35]. The mismatch between the I-V hysteresis curves typically originates from the hysteresis in charge trapping and release dynamics of internal defect states (such as V_O_) within the material [34, 36]. When an external voltage is scanned, the charging and discharging rates of the defect states do not match the rate of voltage change, resulting in different carrier concentrations and transport pathways during forward and reverse scans, thereby forming hysteresis. In other words, the hysteresis curve in a dual-mode device reflects the differences caused by the ionization (V_O_ + 2h^+^ → V_O_^2+^) and de-ionization (V_O_^2+^ → V_O_ + 2h^+^) of V_O_. Therefore, we can use information from the hysteresis curve (such as the hysteresis area) to demonstrate the degree/number of holes involvement in the ionization processes.

Under illumination at 310 nm, electrons in the nanowires are excited to the conduction band, generating free electrons and holes. When comparing the double-sweep I-V curves within the ranges of -2/2/-2 V and -0.2/0.2/-0.2 V, the former curve exhibits a pronounced hysteresis phenomenon, while the latter curve does not show significant hysteresis even in an enlarged view (Figure S7a). Compared to that at small bias voltages (-0.2/0.2/-0.2 V), the electric field intensity is greater at high bias voltages (-2/2/-2 V), overcoming the ionization barriers of V_O_ and thus ionizing more V_O_. Additionally, in the extreme case under dark conditions (Figure S7b and S7c), no photogenerated holes are involved in the ionization of V_O_.

The size of the hysteresis area (*S*_Area_) reflects the scale of V_O_ participating in charge redistribution, which can be calculated as that in Figure S7c. At the high bias voltages, more V_O_ are ionized, leading to a larger difference in carrier concentration during forward and reverse scans. This difference manifests as a larger hysteresis area (Figure S7d). During the transition from a high bias voltage (-2/2/-2 V) to a low bias voltage (-0.2/0.2/-0.2 V), we observe a gradual reduction in the hysteresis area (Figure S7d), which indicates a decreasing number of holes participating in the ionization of V_O_. As a result, Figure S7 provides another perspective that the bias voltage and photogenerated holes participating in the ionization of oxygen vacancies have the significant effects on the hysteresis area.

**Supplementary Note 6. Discussion on the impact of anisotropy of the material.**

**A) Anisotropy compensation of nanowire structures**

GaN-based nanowires indeed exhibit anisotropic optoelectronic properties due to their wurtzite crystal structure. Self-catalyzed growth results in a randomly distributed nanowire array. However, self-catalyzed growth endows each nanowire with its own distinct anisotropy [37, 38], and the interference effects among the randomly distributed nanowires compensate for the overall anisotropy of the material, resulting in the nanowire array does not exhibiting a significant polarization angle response within certain specific azimuthal sectors.

**B) Experimental validation**

Figure S10a shows a schematic diagram of the test setup for polarized light response. As shown in Figure S10b, starting from 0° on the polarizer, the polarizer is adjusted by 30° for each test, up to 330°. All tests are conducted under the same conditions. Figure S10c summarizes the light response intensities at different polarization angles. The average and standard deviation reference lines indicate that the polarization angle has a certain, yet relatively weak, influence on the light response intensity. Therefore, we calculate the sensitivity of the photodetector to polarized light by measuring the polarization extinction ratio (PER = I_∥_/I_⊥_) in a linear form. I_∥_ and I_⊥_ are the output photocurrents when the polarization direction of the light is parallel and perpendicular, respectively, to the polarization-sensitive direction of the detector. The calculated PER is 1.042, indicating that the device is non-polarization-sensitive.

**Supplementary Note 7.** **Discussion on the** **current-voltage (I-V) curve shift under light illumination.**

**A) Charge transfer and built-in electric field**

Due to the difference in work functions between graphene and (Al,Ga)N, electrons transfer from (Al,Ga)N to graphene at the heterojunction interface, resulting in an increase in electron concentration on the graphene side and a decrease on the (Al,Ga)N side [39]. This charge transfer leads to the formation of a built-in electric field at the interface, directed from (Al,Ga)N towards graphene. The presence of the built-in electric field hinders further diffusion of electrons from graphene to (Al,Ga)N, thereby forming a Schottky barrier (q*V*_Bi_) at the interface[40].

**B) Barrier height and built-in electric field in the dark state**

As shown in Figure S12a, during the device testing process, the top electrode of the device was set as the positive electrode (+), and the bottom as the negative electrode (-). In the dark state, there is a Schottky barrier at the graphene/(Al,Ga)N heterojunction interface, whose height can be defined as q*V*_Bi_ (Figure S12b). Due to the built-in electric field pointing from right to left (*E*_Bi_), which is opposite to the direction from the positive to the negative electrodes.

**C) Photovoltage is opposite to that of the built-in electric field.**

As illustrated in Figure S12c, the photogenerated carriers produced by the nanowires under illumination diffuse towards the graphene/(Al,Ga)N heterojunction interface due to a concentration gradient. This results in the formation of a photovoltage (*V*_ph_), which has a field direction opposite to that of the built-in electric field (*E*_ph_). Consequently, the barrier height *q*(*V*_Bi_-*V*_ph_) is reduced, ultimately causing the entire I-V curve to shift rightward on the coordinate axes.

In conclusion, the rightward shift of the I-V curve in the graphene/(Al,Ga)N heterojunction photodetector under illumination is mainly due to the *V*_ph_. The barrier height at the graphene/(Al,Ga)N heterojunction interface changes from *qV*_Bi_ in the dark state to *q*(*V*_Bi_-*V*_ph_) under illumination, resulting in the observed I-V curve shift.

**Supplementary Note 8. Discussion on the effective operation of dual-mode devices in external circuits.**

For effective application, we can adopt several improvements. First, operational amplifiers can convert picoampere (pA)-level photocurrents into measurable voltage signals, such as low-noise FET-input or dedicated transimpedance amplifiers (TIA) as preamplifiers. Second, since pA-level signals are susceptible to environmental noise, we can reduce electromagnetic interference through the use of metal shielded boxes, shielded cables and proper grounding measures. Third, increasing the intensity of the light source or utilizing high-efficiency light-concentrating devices (such as lenses or reflectors) can focus more light signals onto the detector. Additionally, enhancing the current density can be achieved by increasing the light-receiving area of the detector or using an array-type photodetector.

In fact, under a 0 V bias, the ultra-low dark current of the device < 1 pA, while the photocurrent is over 20 pA (Figure S11a and 4e), with light/dark rejection ratios exceeding 20 times, providing our device with the basic conditions for practical application. Moreover, photo detectors with low current density may be used for specific tasks, such as reducing the risk of overexposure in high-light environments [41]. In the application of photodetectors, converting the output current signal to a voltage signal is a common practice. Here are the reasons:

**A) Convenience of signal amplification and processing**

Many electronic devices and data acquisition systems are more suitable to process voltage signals [42, 43]. Converting the current signal to a voltage signal facilitates subsequent signal amplification, filtering, and digital processing. This is particularly important when designing photodetector circuits. Through a current-to-voltage conversion circuit, weak current signals can be effectively converted into voltage signals that are easier to process.

**B) Improved signal-to-noise ratio**

Directly processing weak current signals may be susceptible to noise, reducing measurement accuracy. By converting the current signal to a voltage signal and using an appropriate amplifier circuit, the signal-to-noise ratio can be improved, enhancing the system's anti-interference capability [42].

**C) Compatibility with subsequent circuits**

Many subsequent processing circuits, such as analog-to-digital converters (ADCs) and digital signal processors (DSPs), are typically designed to accept voltage inputs [44, 45]. Converting the current signal to a voltage signal ensures compatibility with these circuits, simplifying system design.

As shown in Figure 4g, our device exhibits a photovoltaic voltage of 210 mV at light illuminations, such a magnitude of signal that can be easily read by the circuit board, thereby enabling effective signal detection. In brief, on the one hand, we can reduce electromagnetic interference in the environment by adding shielded cables or screens in practical applications. On the other hand, we can choose operational amplifiers with extremely low noise and high gain to measure the photovoltaic voltage signals of the device. As shown in Figure 6, under a bias current of 0 A, we successfully utilized the photovoltaic voltage response of the device to drive the humanoid robot for different actions under optical modulation.

**Supplementary Note 9.** **The application potential of GaN-based nanowire devices.**

**A) The significance and application scenarios of working in the UV band**

UV imaging is generally not disturbed by visible light pollution, making it highly suitable for high-brightness environments or scenarios with low contrast (such as target detection against a high-brightness background). Numerous outstanding works have studied the UV photodetectors. For instance, Zhang *et al.* reported a β-Ga_2_O_3_ UV photodetector and its potential for single-pixel imaging in the UV range [46]. Song *et al.* fabricated a MXene/GaN UV photodiode, which shows high potential for many applications, such as imaging, and sensing networks [47]. Additionally, low-dimensional wide-bandgap semiconductors for UV devices have demonstrated their application prospects in imaging, communication, multispectral and/or weak light detection [48]. In brief, our research in this work aims to promote the application development of dual-mode devices in the UV wavelength band, providing new insights for the field of UV multi-mode imaging technology.

**B) Potential capability for bandwidth expansion in applications**

(Al,Ga)N materials with the high bandgap and low dark current are highly suitable for developing high-performance UV photodetectors and neuromorphic devices. This research focuses on the UV band, but the (Al,Ga)N based material system can achieve tunability in the photoresponse range by adjusting the composition ratio (*e.g.*, the content of Al and In), extending from the UV band to visible light ranges. For (In,Ga)N nanowire materials, which theoretically have a maximum operating wavelength covering up to 1,770 nm, applicable for detection and imaging in the visible and near-infrared regions [49].

As a whole, the significance of UV devices in the fields of detection and imaging lies in their ability to capture information that is imperceptible to the human eye. Whether in the microscopic realm of biomedical science or the macroscopic domains of environmental monitoring and astronomy, UV detection technology plays a crucial role.

**Supplementary Note 10. Discussion on the calculation of light source power in terms of power consumption.**

The calculations in this paper solely focus on the operational power consumption of the device itself, excluding the power of the light source. Since the input optical power in the experiment is closely related to the experimental setup, we have also discussed the impact of the light source on the overall power consumption. The energy consumption can be calculated as follows [25]: *E*_SPt_ = *S* × *P_light_* × *t*, where *S* stands for the active area of device (0.0004 cm^-2^). *P* represents the optical density (9.2 μW cm^-2^). *t* indicates the duration of time (0.05 s). The overall power consumption is calculated 0.182 nJ (Table S3). Here, if the goal is to calculate the energy consumption of the entire system, it is indeed necessary to consider both the energy consumption of the device itself and the energy consumption of the light source simultaneously. In fact, based on the aforementioned calculation logic for optical power consumption, we can further reduce the device area to achieve extremely low energy consumption. It is worth noting that, through data analysis, we have found that the device area is a crucial factor determining the optical power consumption of the device. However, in practical applications, the power consumption of the light source is fixed and does not vary with changes in the effective area of the device. In summary, under the same computational conditions, the power consumption of our device is quite low among GaN-based devices.

**Supplementary Note 11. Exploring the transition from STP to LTP.**

The classification of STP and LTP is based on retention time. For instance, STP was defined as synaptic plasticity that disappears for a few seconds or tens of seconds, while LTP was defined as changes that persist for tens of seconds or longer [19, 50]. Thus, to simplify it, STP is defined as synaptic plasticity that disappears within 150s (Figure S19d and e), while LTP refers to a more stable and long-lasting form of synaptic plasticity that persists for a longer period (> 150s) here.

**Supplementary Note 12.** **Performing noise reduction preprocessing using DMS devices at the hardware level.**

**A) The necessity and advantage of this method**

Although using nonlinear response units in software may suffice for some simple experiments, in practical applications, especially in neuromorphic computing where a large amount of image data needs to be processed, hardware acceleration offers irreplaceable advantages [19, 30]. Image preprocessing and noise reduction often require complex computations for each pixel or data point. When these tasks are handled in software, the computational burden is extremely high, especially for high-definition images or video stream data, which can quickly deplete CPU and GPU resources [51]. In contrast, using synaptic device arrays allows for image preprocessing on the hardware side, enabling parallel computation of vast amounts of pixel information, whereas software typically executes tasks sequentially. This allows the hardware to complete large-scale computational tasks within a limited timeframe.

Synaptic devices possess synaptic plasticity, meaning their weights can adaptively change during the training process, which is highly effective for adaptive filters and feature extraction in image denoising. Hardware implementation allows this plasticity to be efficiently mapped onto image data, enabling rapid adaptation to the environment and thus enhancing denoising performance. Compared to traditional nonlinear response devices, synaptic devices can be more naturally adapted to the nonlinear relationships and complex noise patterns present in image data [30]. Indeed, nonlinear response devices can achieve neuromorphic functions to a certain extent. The dual-mode characteristic offers the possibility for multifunctionality in neuromorphic systems. In addition to achieving nonlinear responses, dual-mode devices can efficiently switch between different signal modes (such as photodetector and synapse device), making them more suitable for complex tasks in future multimodal neural networks. By mimicking the multimodal information processing capabilities of biological neural systems, dual-mode devices can simultaneously extract intensity and spectral information during the image preprocessing stage.

**B) Impact on signal-to-noise ratio (SNR)**

Figure 5k, 5l and S23 demonstrate that the overall performance of the neuromorphic network improves after preprocessing, including improvements in image contrast and recognition accuracy. To avoid misunderstanding, an explanation is provided for the terms "Input" and "Output" in Figure 5k and 5l. We artificially added random noise to the images labeled as "Input", thus "Input" represents the images that have not undergone preprocessing. In contrast, "Output" represents the images after noise reduction through the synaptic devices. It is evident that the preprocessed images exhibit suppressed noise and enhanced contrast.

**Supplementary Note 13.** **Discussion between the DMS and the nonlinear response behavior.**

**A) Fulfillment of the requirements for nonlinear response in image preprocessing**

As shown in the Ref. [19, 30, 52], as the light intensity increases, the enhancement of photoconductivity is more significant within a certain range of light intensity, which is the core of such an ability to achieve denoising preprocessing. To our knowledge, there are a limited number of reports on denoising preprocessing of images at the hardware level based on such nonlinear responses. Specifically, in Ref [30], Zhou et al. present that four light intensities of 0 mW cm^−2^ (pixel A), 0.22 mW cm^−2^ (pixel C) and 0.88 mW cm^−2^ (pixel B), 0.45 mW cm^−2^ (pixel D) are normalized to 0-1. Thus, the input optical signals of the four pixels A, B, C and D are 0, 0.25, 0.5 and 1, respectively. Therefore, after normalization of the currents from 0 to 1, the output currents of pixels A, B, C and D were 0, 0.13, 0.39 and 1, respectively.

As a schematic diagram illustrating the principle, we have conducted a simple classification of synaptic device's nonlinear response characteristics (curves) into three categories, and have depicted the corresponding input and output responses. As shown in Figure S22a, it represents the input signal. Figure S22b demonstrates the three response characteristics of the synaptic device, labeled as (i), (ii) and (iii), respectively. Figure S22c presents the response results after the signal passes through the synaptic device. It is evident that if the noise in Figure S22a is 0.5, after undergoing the three different response types (Figure S22c), the result in (i) may show as 0.8, the result in (ii) is obviously 0.5, and the result in (iii) may be 0.2. It is evident that only the noise in the (iii) type of response characteristics is suppressed. Additionally, in most synaptic devices or other devices with nonlinear responses, noise is introduced at the software end during the process of ANN image recognition for simulation and modeling purposes [53-55]. In brief, the preprocessing of images using the aforementioned principle has specific requirements for the response characteristics of the device. As shown in Figure S21a-c and S23b, our device meets the response characteristics.

**B) Ionization of oxygen vacancies**

In this work, oxygen vacancies (V_O_) serve as the critical defect centers in (Al,Ga)N/GaN nanowires, and their degree of ionization directly impacts the photoresponse characteristics. At low light intensities, the number of photogenerated carriers is relatively small, resulting in partial ionization of Vo. As the light intensity increases, more photogenerated carriers are generated, leading to a higher degree of ionization of Vo. Consequently, as the illumination intensifies, an increasing number of stable positive charge centers (Vo^2+^) is gradually formed in the (Al,Ga)N/GaN nanowires. Carrier recombination is further suppressed under the influence of local electric fields, with this effect being more pronounced under intense light. This results in nonlinear photoresponse characteristics in one hand.

**C) Filling of surface defects**

(Al,Ga)N/GaN nanowires possess a large surface-to-volume ratio, and the incomplete coordination of surface atoms easily leads to the formation of surface or trap states [56]. Under low light conditions, surface defects are less filled. As light intensity increases, more surface defects are filled, reducing the confinement of traps on photogenerated carriers and leading to an increase in the concentration of free carriers. This effect is another important cause of nonlinear enhancement.

In summary, the nonlinearity effect of photoresponse current is closely related to the dynamic balance between carrier generation, surface-defect filling, and Vo ionization within the device.

**Supplementary Note 14. Reliability of simulation results.**

This work conducts simulation analysis of artificial neural networks (ANNs) based on experimental data and device characteristics, with the aim of evaluating the potential application performance of dual-mode devices in ANNs. B) Simulation analysis, as a crucial step in device optimization and functional verification, can effectively screen parameters and design schemes at early stages, reducing the complexity and time costs of experimental development. Some important works related to artificial synapse device have also conducted relatively simple verification experiments in potential application sections [30, 57, 58]. C) Our simulation analysis is based on physical models and incorporates actual measured response data (such as nonlinear photoelectric response characteristics), ensuring the physical significance and rationality of the simulation results (Figure S21).

**Supplementary Note 15. Description of a method for encoding image input into multiple pulse signals.**

Handwritten digit images are typically grayscale, and the grayscale value of each pixel can be mapped to the intensity of light pulses. Here, we intend to use amplitude modulation to directly map grayscale values to light intensity. With grayscale values already encoded, UV Micro-LEDs can be used to display handwritten digits since our synaptic devices operate in the UV range. If we need to display an 8×8 image, only 64 light-emitting units in the Micro-LED display would be active. It is worth noting that since grayscale values are mapped to light intensity, and the driving current can directly control the light-emitting intensity of LEDs, we apply different currents to the light-emitting units on the driving circuit board according to the mapping relationship to control the output image. A multi-channel modulator map pixels from different regions of an image into independent streams of light pulses. Through the control of the driving circuit, we can keep the handwritten digits constantly lit or make them emit light periodically. Ultimately, the image information can be captured by an array of synaptic devices.

**Supplementary Note 16. Cascaded design for current convert to voltage.**

Using a transimpedance amplifier (TIA) as the core circuit, we can convert weak nA-level current signals into voltage signals. First, a precision resistor with a feedback resistance in the range of 10^9^ ohms (GΩ) is used to ensure sufficiently high gain, converting the nA-level current into a voltage in the mV range. An operational amplifier with low bias current and high input impedance, such as the AD549 from Analog Devices Inc. (ADI) or the OPA827 from Texas Instruments (TI), is selected to reduce the impact of thermal noise and 1/f noise. The bandwidth of the TIA is adjusted to match the dynamic characteristics of the current signal while avoiding interference from high-frequency noise. Second, to address the issue of weak signals being susceptible to noise interference, we can use an electromagnetic shielding box to reduce environmental electromagnetic interference. A low-pass filter is added at the output of the TIA to suppress high-frequency noise and improve the signal-to-noise ratio. Single-point grounding and the separation of signal ground from power ground are adopted to reduce noise introduced by grounding loops. In summary, by combining a high-sensitivity TIA circuit with noise suppression techniques, we can ensure accurate voltage signal generated by synaptic device arrays.

**References**

1. S. Fang *et al.*, Tuning the Charge Transfer Dynamics of the Nanostructured GaN Photoelectrodes for Efficient Photoelectrochemical Detection in the Ultraviolet Band. *Adv. Funct. Mater.* **31**, 2103007 (2021).

2. D. Wang *et al.*, Pt/AlGaN Nanoarchitecture: Toward High Responsivity, Self-Powered Ultraviolet-Sensitive Photodetection. *Nano lett.* **21**, 120-129 (2020).

3. M. Jiang *et al.*, Self-Powered Photoelectrochemical (Al,Ga)N Photodetector with an Ultrahigh Ultraviolet/Visible Reject Ratio and a Quasi-Invisible Functionality for 360° Omnidirectional Detection. *ACS Photonics* **8**, 3282−3290 (2021).

4. M. Jiang *et al.*, Flexible Self-Powered Photoelectrochemical Photodetector with Ultrahigh Detectivity, Ultraviolet/Visible Reject Ratio, Stability, and a Quasi-Invisible Functionality Based on Lift-Off Vertical (Al,Ga)N Nanowires. *Adv. Mater. Interfaces* **9**, 2200028 (2022).

5. D. Wang *et al.*, Observation of polarity-switchable photoconductivity in III-nitride/MoSx core-shell nanowires. *Light-Sci. Appl.* **11**, 227 (2022).

6. J. Zhang *et al.*, Self-powered (In,Ga)N-nanowire-based photodetector with fast response speed for under-seawater detection. *Optics Express* **31**, 8128-8138 (2023).

7. M. Jiang *et al.*, Flexible bidirectional self-powered photodetector with significantly reduced volume and accelerated response speed based on hydrogel and lift-off GaN-based nanowires. *Fundamental Res.* **4**, 369-378 (2024).

8. J. Xue *et al.*, Achieving a High-Responsivity and Fast-Response-Speed Solar-Blind Photodetector for Underwater Optical Communication via AlGaN/AlN/GaN Heterojunction Nanowires. *ACS Appl. Electro. Mater.* **6**, 4643-4652 (2024).

9. H. Chen *et al.*, High-Responsivity Natural-Electrolyte Undersea Photoelectrochemical Photodetector with Self-Powered Cu@GaN Nanowires Network. *Adv. Funct. Mater.* **33**, 2302872 (2023).

10. X. Pan, R. Deng, H. Hong, M. Luo, R. Notzel, Sign reversal of visible to UV photocurrent in core-shell n-InGaN/p-GaN nanowire photodetectors. *J. Appl. Phys.* **136**, 034501 (2024).

11. M. Luo *et al.*, Ultraviolet photoelectrochemical photodetector based on GaN/Cu2O core-shell nanowire p-n heterojunctions. *AIP Advances* **12**, 115112 (2022).

12. S. Ding *et al.*, Self-Powered Solar-Blind Photodetectors Based on Vertically Aligned GaN@Ga2O3 Core–Shell Nanowire Arrays. *ACS Appl. Nano Mater.* **5**, 14470-14477 (2022).

13. J. Zhang *et al.*, Enhance the responsivity and response speed of self-powered ultraviolet photodetector by GaN/CsPbBr_3_ core-shell nanowire heterojunction and hydrogel. *Nano Energy* **100**, 107437 (2022).

14. S. Wang *et al.*, Structural designs of AlGaN/GaN nanowire-based photoelectrochemical photodetectors: carrier transport regulation in GaN segment as current flow hub. *Adv. Photonics Nexus* **2**, 036003 (2023).

15. H. Yu *et al.*, One Stone, Three Birds: SnO_2_ Nanosheet Arrays toward Self-Powered Visible-Blind UV Photodetection with High Responsivity and Rejection Ratio. *Adv. Opt. Mater.* **12**, 2301460 (2024).

16. Z. Junhua *et al.*, Solar-blind ultraviolet photodetection of an a-Ga_2_O_3_ nanorod array based on photoelectrochemical self-powered detectors with a simple, newly-designed structure. *J. Mater. Chem. C* **7**, 6867-6871 (2019).

17. M. Zhang *et al.*, Ultrathin In_2_O_3_ Nanosheets toward High Responsivity and Rejection Ratio Visible-Blind UV Photodetection. *Small* **19**, 2205623 (2023).

18. X. Han *et al.*, Amorphous Ga2O3/GaN heterostructure for ultralow-energy-consumption optically stimulated synaptic devices. *Appl. Phys. Lett.* **124**, 1077-3118 (2024).

19. S. Feng *et al.*, Dual‐Mode Conversion of Photodetector and Neuromorphic Vision Sensor via Bias Voltage Regulation on a Single Device. *Adv. Mater.* **35**, 2308090 (2023).

20. M. Zhou *et al.*, Realize low-power artificial photonic synapse based on (Al,Ga)N nanowire/graphene heterojunction for neuromorphic computing. *APL Photonics* **8**, 076107 (2023).

21. X. Gu *et al.*, Realize ultralow-energy-consumption photo-synaptic device based on a single (Al,Ga)N nanowire for neuromorphic computing. *Nano Res.* **17**, 1933-1941 (2023 ).

22. Y. Z. Min Zhou, XiuShuo Gu,QianYi Zhang, Min Jiang, Shulong Lu, Light-stimulated low-power artificial synapse based on a single GaN nanowire for neuromorphic computing. *Photonics Res.* **11**, 1667 (2023).

23. Q. Hua *et al.*, Piezotronic Synapse Based on a Single GaN Microwire for Artificial Sensory Systems. *Nano Lett.* **20**, 3761-3768 (2020).

24. Y. Mo, B. Luo, H. Dong, B. Hou, Light-stimulated artificial synapses based on Si-doped GaN thin films. *J. Mater. Chem. C* **10**, 13099-13106 (2022).

25. C. Kai *et al.*, AlGaN/GaN‐Based Optoelectronic Synaptic Devices for Neuromorphic Computing. *Adv. Opt. Mater.* **11**, 2202105 (2023).

26. C. H. Huang, C. Y. Wu, Y. F. Lin, Y. C. Chou, K. T. Lee, Wet‐Etching‐Boosted Charge Storage in 1D Nitride‐Based Systems for Imitating Biological Synaptic Behaviors. *Adv. Funct. Mater.* **33**, 2306030 (2023).

27. L. Hu *et al.*, Ultrasensitive Freestanding and Mechanically Durable Artificial Synapse with Attojoule Power Based on Na-Salt Doped Polymer for Biocompatible Neuromorphic Interface. *Adv. Funct. Mater.* **31**, 2106015 (2021).

28. L. Jiang *et al.*, Helical Nanofiber Photoelectric Synaptic Devices for an Artificial Vision Nervous System. *Nano Lett.* **23**, 8146-8154 (2023).

29. P. Xie *et al.*, Ferroelectric P(VDF-TrFE) wrapped InGaAs nanowires for ultralow-power artificial synapses. *Nano Energy* **91**, 106654 (2022).

30. F. C. Zhou *et al.*, Optoelectronic resistive random access memory for neuromorphic vision sensors. *Nat. Nanotechnol.* **14**, 776-782 (2019).

31. K. Deng *et al.*, Effective Suppression of Amorphous Ga2O and Related Deep Levels on the GaN Surface by High-Temperature Remote Plasma Pretreatments in GaN-Based Metal–Insulator–Semiconductor Electronic Devices. *ACS Appl. Mater. Interfaces* **15**, 25058-25065 (2023).

32. Z. Zhang *et al.*, GaN as an Interfacial Passivation Layer: Tuning Band Offset and Removing Fermi Level Pinning for III-V MOS Devices. *ACS Appl. Mater. Interfaces* **7**, 5141-5149 (2015).

33. Y. Dong, R. M. Feenstra, J. E. Northrup, Electronic states of oxidized GaN(0001) surfaces. *Appl. Phys. Lett.* **89**, 171920 (2006).

34. J. Bisquert, Current-controlled memristors: Resistive switching systems with negative capacitance and inverted hysteresis. *Phys. Rev. Appl.* **20**, 044022 (2023).

35. Y. Takishita *et al.*, Memristor property of an amorphous Sn-Ga-O thin-film device deposited using mist chemical-vapor-deposition method. *AIP Adv.* **10**, 035112 (2020).

36. D. H. Vieira *et al.*, Ecofriendly Printed Wood-Based Honey-Gated Transistors for Artificial Synapse Emulation. *Adv. Intell. Syst.* **7**, (2025).

37. L. Rigutti *et al.*, Photoluminescence polarization properties of single GaN nanowires containing Al_x_Ga_1-x_N/GaN quantum discs. *Phys. Rev. B* **81**, 045411 (2010).

38. M. Persson, A. Di Carlo, Electronic structure and optical properties of freestanding [0001] oriented GaN nanowires and nanotubes. *J. Appl. Phys.* **104**, 073718 (2008).

39. H. Parimoo, Q. Zhang, M. Vafadar, J. Sivasundarampillai, S. Zhao, AlGaN nanowire deep ultraviolet light emitting diodes with graphene electrode. *Appl. Phys. Lett.* **120**, 171108 (2022).

40. B. Pandit, J. Kim, J. Cho, Determination of Schottky barrier height of graphene electrode on AlGaN/GaN heterostructure. *AIP Advances* **11**, 045314 (2021).

41. M. Chen *et al.*, An Ultrasensitive Bi2O2Se/In2S3 Photodetector with Low Detection Limit and Fast Response toward High-Precision Unmanned Driving. *ACS Nano* **18**, 27579-27589 (2024).

42. Y. Liu, S. Wang, L.-M. Peng, Toward High-Performance Carbon Nanotube Photovoltaic Devices. *Adv. Energy Mater.* **6**, 1600522 (2016).

43. B. Lv *et al.*, Local large temperature difference and ultra-wideband photothermoelectric response of the silver nanostructure film/carbon nanotube film heterostructure. *Nat. Commun.* **13**, 1835- (2022).

44. W. Jiang *et al.*, Real-Time Deployment of Simplified Volterra Nonlinear Equalizer in High-Speed PON. *IEEE Photonics Technol. Lett.* **35**, 1067-1070 (2023).

45. G. Yedukondalu, S. Samanta, Effective phase utilization and efficiency improvement of high power interleaved DC-DC converter using modified rotating phase control. *Comput. Electr. Eng.* **110**, 108837 (2023).

46. N. Zhang *et al.*, Under-Seawater Immersion β-Ga_2_O_3_ Solar-Blind Ultraviolet Imaging Photodetector with High Photo-to-Dark Current Ratio and Fast Response. *ACS Nano* **18**, 652-661 (2023).

47. W. Song, J. Chen, Z. Li, X. Fang, Self-Powered MXene/GaN van der Waals Heterojunction Ultraviolet Photodiodes with Superhigh Efficiency and Stable Current Outputs. *Adv. Mater.* **33**, 2101059 (2021).

48. Z. Li, T. Yan, X. Fang, Low-dimensional wide-bandgap semiconductors for UV photodetectors. *Nat. Rev. Mater.* **8**, 587-603 (2023).

49. J. Min *et al.*, Bottom-Up Formation of III-Nitride Nanowires: Past, Present, and Future for Photonic Devices. *Adv. Mater.* **36**, 2405558 (2024).

50. K. Chen *et al.*, Organic optoelectronic synapse based on photon-modulated electrochemical doping. *Nat. Photonics* **17**, 629-637 (2023).

51. W. Zhang *et al.*, Edge learning using a fully integrated neuro-inspired memristor chip. *Science* **381**, 1205-1211 (2023).

52. J. Jiang *et al.*, Hardware-Level Image Recognition System Based on ZnO Photo-Synapse Array with the Self-Denoising Function. *Adv. Funct. Mater.* **34**, 2313507 (2024).

53. Y. X. Hou *et al.*, Large-Scale and Flexible Optical Synapses for Neuromorphic Computing and Integrated Visible Information Sensing Memory Processing. *ACS Nano* **15**, 1497-1508 (2021).

54. A. Bednarkiewicz *et al.*, All‐Optical Data Processing with Photon‐Avalanching Nanocrystalline Photonic Synapse. *Adv. Mater.* **35**, 2304390 (2023).

55. W. Zhao *et al.*, Perovskite-Doped Modulated Color-Selective Photosynaptic Transistors for Target Object Recognition. *Nano Lett.* **24**, 9937-9945 (2024).

56. W. Chen *et al.*, Surface treatment of GaN nanowires for enhanced photoelectrochemical water-splitting. *Chin. Chem. Lett.* **35**, 109168 (2024).

57. C. Zhu *et al.*, Optical synaptic devices with ultra-low power consumption for neuromorphic computing. *Light-Sci. Appl.* **11**, 337 (2022).

58. H. Jiang *et al.*, Simultaneous achieving negative photoconductivity response and volatile resistive switching in Cs_2_CoCl_4_ single crystals towards artificial optoelectronic synapse. *Light-Sci. Appl.* **13**, 316 (2024).
